# Efficient Syntheses of 1,2,3-Triazoloamide Derivatives Using Solid- and Solution-Phase Synthetic Approaches

**DOI:** 10.3390/molecules201119673

**Published:** 2015-11-05

**Authors:** Doohyun Lee, Daehun Kim, Seungyeon Lee, Taegeum Kim, Joobin Kim, Sohee Kim, Kwang-Hyeon Liu, Sangkyu Lee, Jong-Sup Bae, Kyung-Sik Song, Chang-Woo Cho, Youn Kyung Son, Dong Jae Baek, Taeho Lee

**Affiliations:** 1 College of Pharmacy, Research Institute of Pharmaceutical Sciences, Kyungpook National University, 80 Daehak-ro, Buk-gu, Daegu 702-701, Korea; newkiy@hanmail.net (D.L.); eogns1201@nate.com (D.K.); tmddusj@naver.com (S.L.); eksvnddlv321@naver.com (T.K.); joobin87@naver.com (J.K.); ksh71051@naver.com (S.K.); dstlkh@knu.ac.kr (K.-H.L.); sangkyu@knu.ac.kr (S.L.); baejs@knu.ac.kr (J.-S.B.); kssong@knu.ac.kr (K.-S.S.); 2 Department of Chemistry, Kyungpook National University, 80 Daehak-ro, Buk-gu, Daegu 702-701, Korea; cwcho@knu.ac.kr; 3 National Institute of Biological Resources, Hwangyeong-ro 42, Seo-gu, Incheon 404-708, Korea; sophy004@korea.kr; 4 College of Pharmacy, Natural Medicine Research Institute, Mokpo National University, 1666 Youngsan-ro, Muan-gun, Jeonnam 534-729, Korea

**Keywords:** 1,2,3-triazoloamide, solid-phase synthesis, solution-phase synthesis

## Abstract

Efficient synthetic routes for the preparation of secondary and tertiary 1,2,3-triazoloamide derivatives were developed. A secondary α-1,2,3-triazoloamide library was constructed and expanded by a previously developed solid-phase synthetic route and a tertiary 1,2,3-triazoloamide library was constructed by a parallel solution-phase synthetic route. The synthetic routes rely on amide formation with secondary amines and chloro-acid chlorides; S_N_2 reaction with sodium azide; and the selective [3 + 2] Hüisgen cycloaddition with appropriate terminal alkynes. The target secondary and tertiary 1,2,3-triazoloamide derivatives were obtained with three-diversity points in excellent overall yields and purities using the reported solid- and solution-phase synthetic routes, respectively.

## 1. Introduction

Combinatorial chemistry has emerged as a powerful technique for the synthesis of biologically active small molecules for the purpose of medicinal chemistry programs within the pharmaceutical industry [1,2,3,4,5]. Recently, the 1,2,3-triazole moiety, produced by Cu(I)-catalyzed [3 + 2] cycloaddition reactions, has been used as a scaffold for generating combinatorial libraries [6,7,8,9,10]. 1,2,3-Triazoles can mimic the topological and electronic features of an amide bond, and this be used as bioisosteres of the amide moiety. They are particularly stable to reduction, oxidation, and hydrolysis conditions.

Various α-1,2,3-triazoloamide derivatives have been shown to exhibit a wide range of biological activities [11,12,13,14,15,16,17,18,19]. In recent examples, α-1,2,3-triazoloamide related compounds have been developed and studied as tropomysin receptor kinase A (TrkA) inhibitors [11], as inhibitors of *Mycobacterium tuberculosis* [12], as phosphodiesterase 4B (PDE4B) inhibitor for anticancer agents [13], as quorum sensing modulators [14], as β-haematin inhibitors for antimalarial agents [15], as γ-secretase modulators [16], as protein tyrosine phosphatase (PTPs) inhibitors [17], as lymphoid tyrosin phosphatase (Lyp, PTPN22) inhibitors [18], and as glucokinase (GK) acitvators [19].

**Figure 1:**
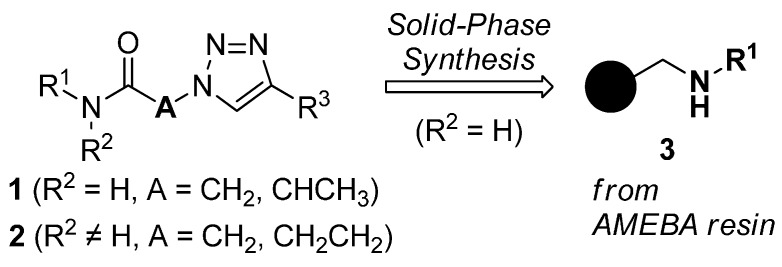
Structure and use of 1,2,3-triazoloamides **1** and **2**.

Previously, we have reported a solid-phase synthetic protocol for the preparation of secondary α-1,2,3-triazoloamides **1** (R^2^ = H, Figure 1) [20]. However, an expanded α-1,2,3-triazoloamide library was needed for our drug discovery project, which includes the secondary and tertiary 1,2,3-triazoloamides. Herein, we describe the construction of expanded libraries of secondary α-1,2,3-triazoloamides **1** on solid-phase and of tertiary 1,2,3-triazoloamides **2** in parallel solution-phase, which is applicable to high-throughput construction of drug-like compound libraries.

## 2. Results and Discussion

**Scheme 1:**
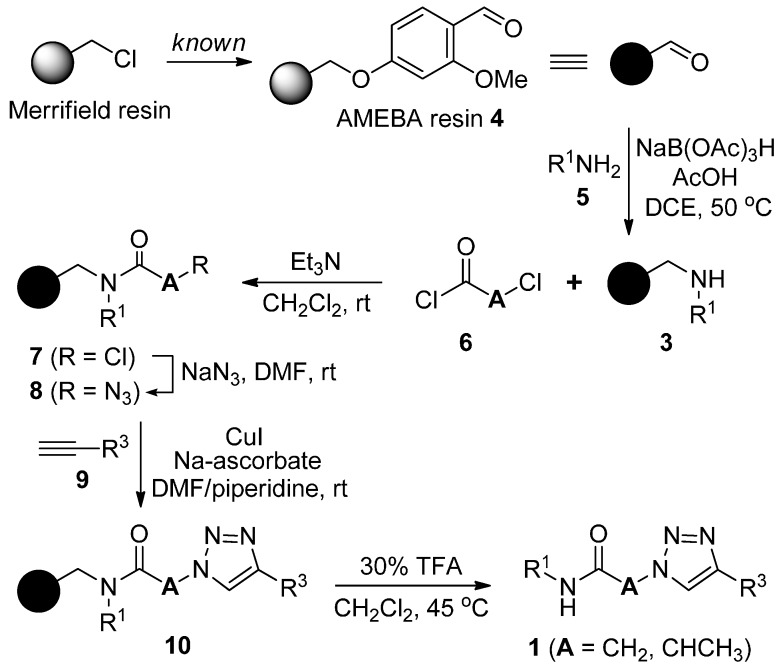
Solid-phase synthesis of secondary α-1,2,3-triazoloamide derivatives **1**.

**Figure 2:**
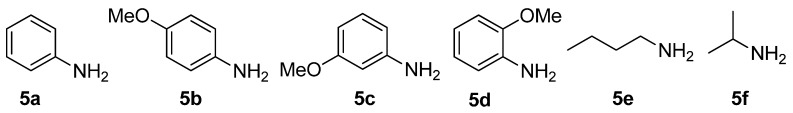
Diversity reagents **5** for secondary α-1,2,3-triazoloamides **1**.

**Figure 3:**
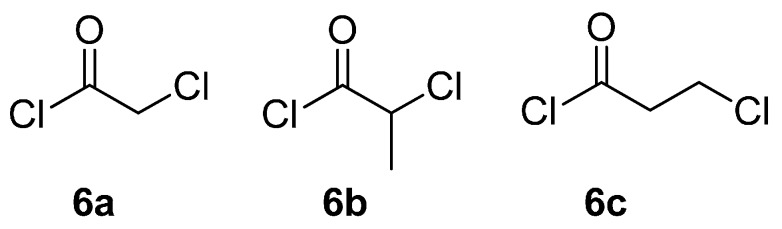
Diversity reagents **6** for 1,2,3-triazoloamides **1** and **2**.

The synthetic sequence for secondary α-1,2,3-triazoloamides **1** (R^2^ = H) is shown in Scheme 1 [20]. According to the solid-phase synthetic approach with the polymer-bound amines **3**, which were prepared by reductive amination reaction from Acid sensitive Methoxy Benzaldehyde (AMEBA) [20,21] resin **4** and primary amines **5** (the first diversity element R^1^; Figure 2), polymer-bound chloroamides **7** can be easily prepared by the reaction of amine resin **3** with chloro-acid chloride **6** (the second diversity element **A**; Figure 3) and triethylamine in CH_2_Cl_2_ at room temperature. Treatment of solid supported chloroamides **7** (R = Cl, **A** = CH_2_ or CHCH_3_) with sodium azide in DMF at room temperature, provides the α-azidoamide resin **8** (R = N_3_).

**Scheme 2:**
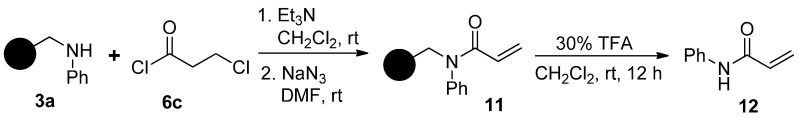
Reaction of amine resin **3a** and 3-chloropropionyl chloride (**6c**).

In the case of β-chloroamide **7ac**, which was prepared by the reaction of amine resin **3a** and 3-chloropropionyl chloride (**6c**), the S_N_2 reaction with sodium azide gave the undesired acrylamide **12** because of an elimination of β-chloroamide (Scheme 2). The reaction was confirmed by ATR-FTIR analysis of resin **11** and the cleavage of the resin **11** under 30% TFA in CH_2_Cl_2_ at room temperature provided an *N*-phenylacrylamide (**12**) [22,23] as a major product.

**Figure 4:**
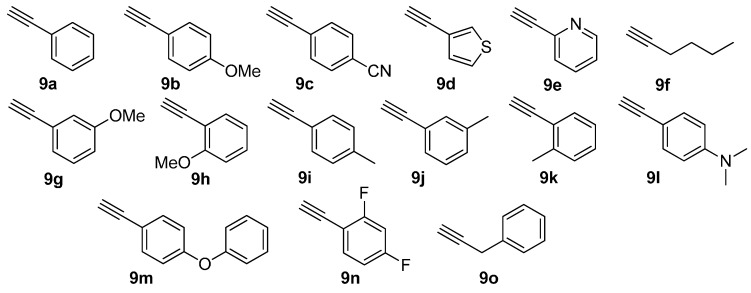
Diversity reagents **9** for 1,2,3-triazoloamides **1** and **2**.

**Scheme 3:**
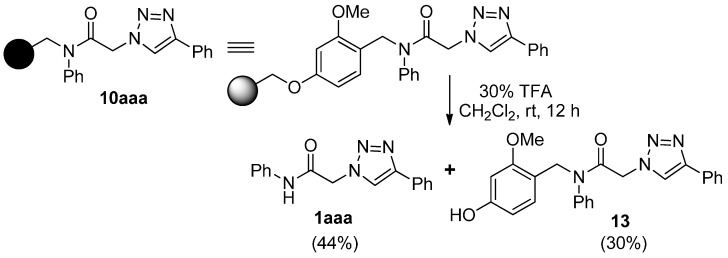
Cleavage of α-1,2,3-triazoloamide resin **10aaa**.

The selective [3 + 2] Hüisgen cycloaddition [24,25,26,27,28,29] was performed with α-azidoamide resin **8** and terminal acetylene **9** (the third diversity element R^3^; Figure 4) according to optimized reaction condition (3 equiv. CuI, 3 equiv. sodium ascorbate, DMF/piperidine (4:1), room temperature) [20]. The well-known methods for the synthesis of 1,2,3-triazoles (catalytic CuSO_4_/sodium ascorbate or CuI/diisopropylethylamine as reagents and H_2_O/*t*-BuOH, EtOH, or THF as solvent) were not very efficient. Under the general cleavage conditions of AMEBA resin (30% TFA, CH_2_Cl_2_, room temperature), the resulting polymer-bound product **10aaa** gave the desired α-1,2,3-triazoloamide **1aaa** (44%) and by-product **13** (30%), while unreacted resin **10aaa** remained as was confirmed by ATR-FTIR analysis (Scheme 3).

Finally, the α-1,2,3-triazoloamide resin **10aaa** was cleaved from the solid support under 30% TFA in CH_2_Cl_2_ at 45 °C to provide the desired α-1,2,3-triazoloamide **1aaa** [24,27,30] (93% over six steps, from Merrifield resin) without formation of by-product **13**.

**Figure 5:**
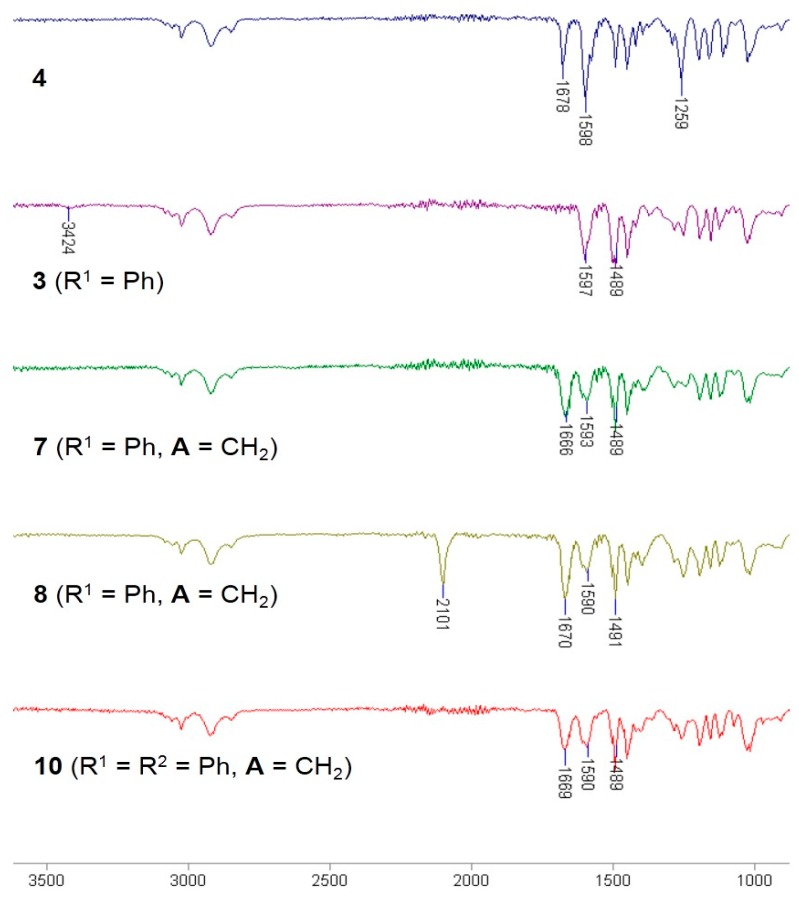
ATR-FTIR spectra of resins **3**, **4**, **7**, **8** and **10** (R^1^ = R^2^ = Ph, **A** = CH_2_).

The reaction progress on solid-phase was monitored by ATR-FTIR (Figure 5). The progress of reductive amination of AMEBA resin **4** and amine **5a** (R^1^ = Ph) was checked by the appearance of the weak NH stretching band at 3424 cm^−1^ and the disappearance of the aldehyde stretching band at 1678 cm^−1^. The progression of amide formation for **7aa** (R^1^ = Ph, **A** = CH_2_) was monitored by ATR-FTIR which displayed the disappearance of the characteristic NH band at 3424 cm^−1^ and appearance of the amide carbonyl stretching band at 1666 cm^−1^. The S_N_2 reaction of **7aa** (R^1^ = Ph, **A** = CH_2_) with sodium azide was monitored by the appearance of the azide stretching band at 2101 cm^−1^. The completion of selective [3 + 2] Hüisgen cycloaddition of **7aa** and **9a** was confirmed by the disappearance of the azide stretching band.

**Table 1:** Prepared secondary α-1,2,3-triazoloamide derivatives **1** ^a^. 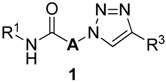

Entry	Products	R^1^	A	R^3^	Yield (%) ^b^	Entry	Products	R^1^	A	R^3^	Yield (%) ^b^
1	**1aaa**	Ph	CH_2_	Ph	93	41	**1dan**	2-MeO-Ph	CH_2_	2,4-di-F-Ph	94
2	**1aab**	Ph	CH_2_	4-MeO-Ph	91	42	**1dao**	2-MeO-Ph	CH_2_	Bn	88
3	**1aac**	Ph	CH_2_	4-CN-Ph	83	43	**1eaa**	*n*-Bu	CH_2_	Ph	81
4	**1aad**	Ph	CH_2_	3-thiophenyl	82	44	**1eab**	*n*-Bu	CH_2_	4-MeO-Ph	88
5	**1aae**	Ph	CH_2_	2-pyridyl	85	45	**1eac**	*n*-Bu	CH_2_	4-CN-Ph	83
6	**1aaf**	Ph	CH_2_	*n*-Bu	92	46	**1ead**	*n*-Bu	CH_2_	3-thiophenyl	80
7	**1baa**	4-MeO-Ph	CH_2_	Ph	92	47	**1eae**	*n*-Bu	CH_2_	2-pyridyl	89
8	**1bab**	4-MeO-Ph	CH_2_	4-MeO-Ph	94	48	**1eaf**	*n*-Bu	CH_2_	*n*-Bu	89
9	**1bac**	4-MeO-Ph	CH_2_	4-CN-Ph	89	49	**1faa**	*i*-Pr	CH_2_	Ph	79
10	**1bad**	4-MeO-Ph	CH_2_	3-thiophenyl	85	50	**1fab**	*i*-Pr	CH_2_	4-MeO-Ph	87
11	**1bag**	4-MeO-Ph	CH_2_	3-MeO-Ph	87	51	**1fac**	*i*-Pr	CH_2_	4-CN-Ph	75
12	**1baj**	4-MeO-Ph	CH_2_	3-Me-Ph	89	52	**1fad**	*i*-Pr	CH_2_	3-thiophenyl	77
13	**1bak**	4-MeO-Ph	CH_2_	2-Me-Ph	84	53	**1fae**	*i*-Pr	CH_2_	2-pyridyl	84
14	**1bal**	4-MeO-Ph	CH_2_	4-NMe_2_-Ph	79	54	**1faf**	*i*-Pr	CH_2_	*n*-Bu	83
15	**1bam**	4-MeO-Ph	CH_2_	4-PhO-Ph	92	55	**1dba**	2-MeO-Ph	CHCH_3_	Ph	88
16	**1ban**	4-MeO-Ph	CH_2_	2,4-di-F-Ph	87	56	**1dbb**	2-MeO-Ph	CHCH_3_	4-MeO-Ph	91
17	**1bao**	4-MeO-Ph	CH_2_	Bn	85	57	**1dbc**	2-MeO-Ph	CHCH_3_	4-CN-Ph	87
18	**1caa**	3-MeO-Ph	CH_2_	Ph	91	58	**1dbd**	2-MeO-Ph	CHCH_3_	3-thiophenyl	80
19	**1cab**	3-MeO-Ph	CH_2_	4-MeO-Ph	90	59	**1dbe**	2-MeO-Ph	CHCH_3_	2-pyridyl	83
20	**1cac**	3-MeO-Ph	CH_2_	4-CN-Ph	87	60	**1dbg**	2-MeO-Ph	CHCH_3_	3-MeO-Ph	94
21	**1cad**	3-MeO-Ph	CH_2_	3-thiophenyl	76	61	**1dbh**	2-MeO-Ph	CHCH_3_	2-MeO-Ph	89
22	**1cae**	3-MeO-Ph	CH_2_	2-pyridyl	81	62	**1dbi**	2-MeO-Ph	CHCH_3_	4-Me-Ph	94
23	**1cah**	3-MeO-Ph	CH_2_	2-MeO-Ph	83	63	**1dbj**	2-MeO-Ph	CHCH_3_	3-Me-Ph	83
24	**1caj**	3-MeO-Ph	CH_2_	3-Me-Ph	93	64	**1dbk**	2-MeO-Ph	CHCH_3_	2-Me-Ph	74
25	**1cak**	3-MeO-Ph	CH_2_	2-Me-Ph	90	65	**1dbl**	2-MeO-Ph	CHCH_3_	4-NMe_2_-Ph	82
26	**1cal**	3-MeO-Ph	CH_2_	4-NMe_2_-Ph	91	66	**1dbm**	2-MeO-Ph	CHCH_3_	4-PhO-Ph	87
27	**1cam**	3-MeO-Ph	CH_2_	4-PhO-Ph	91	67	**1dbn**	2-MeO-Ph	CHCH_3_	2,4-di-F-Ph	92
28	**1can**	3-MeO-Ph	CH_2_	2,4-di-F-Ph	92	68	**1dbo**	2-MeO-Ph	CHCH_3_	Bn	86
29	**1cao**	3-MeO-Ph	CH_2_	Bn	88	69	**1eba**	*n*-Bu	CHCH_3_	Ph	83
30	**1daa**	2-MeO-Ph	CH_2_	Ph	94	70	**1ebb**	*n*-Bu	CHCH_3_	4-MeO-Ph	81
31	**1dab**	2-MeO-Ph	CH_2_	4-MeO-Ph	90	71	**1ebc**	*n*-Bu	CHCH_3_	4-CN-Ph	79
32	**1dac**	2-MeO-Ph	CH_2_	4-CN-Ph	86	72	**1ebd**	*n*-Bu	CHCH_3_	3-thiophenyl	78
33	**1dad**	2-MeO-Ph	CH_2_	3-thiophenyl	91	73	**1ebe**	*n*-Bu	CHCH_3_	2-pyridyl	84
34	**1dae**	2-MeO-Ph	CH_2_	2-pyridyl	84	74	**1ebf**	*n*-Bu	CHCH_3_	*n*-Bu	81
35	**1dag**	2-MeO-Ph	CH_2_	3-MeO-Ph	86	75	**1fba**	*i*-Pr	CHCH_3_	Ph	88
36	**1dah**	2-MeO-Ph	CH_2_	2-MeO-Ph	80	76	**1fbb**	*i*-Pr	CHCH_3_	4-MeO-Ph	83
37	**1dai**	2-MeO-Ph	CH_2_	4-Me-Ph	78	77	**1fbc**	*i*-Pr	CHCH_3_	4-CN-Ph	78
38	**1daj**	2-MeO-Ph	CH_2_	3-Me-Ph	85	78	**1fbd**	*i*-Pr	CHCH_3_	3-thiophenyl	75
39	**1dal**	2-MeO-Ph	CH_2_	4-NMe_2_-Ph	88	79	**1fbe**	*i*-Pr	CHCH_3_	2-pyridyl	81
40	**1dam**	2-MeO-Ph	CH_2_	4-PhO-Ph	92	80	**1fbf**	*i*-Pr	CHCH_3_	*n*-Bu	82

^a^ All reactions were performed on 150–200 mg scale of resin **10** and the purities of compounds **1** were over 95% as judged from LC-MS traces (integration of diode array 200–400 nm traces); ^b^ Six-step overall yield from Merrifield resin (loading capacity = 0.94 mmol/g).

Following the optimized solid-phase synthetic route, the secondary α-1,2,3-triazoloamide derivatives **1** were prepared starting from Merrifield resin and appropriate primary amines **5** (R^1^NH_2_; Figure 2), α-chloroacetyl chlorides **6a** and **6b** (Cl-**A**-COCl; Figure 3), and terminal acetylenes **9** (R^3^C≡CH; Figure 4) and the products displayed in Table 1. In most cases, secondary α-1,2,3-triazoloamide derivatives **1** (80 examples) were obtained with high yields (94%–75%) and high purities, >95% as judged from LC-MS traces (integration of 200–400 nm diode array traces).

**Scheme 4:**
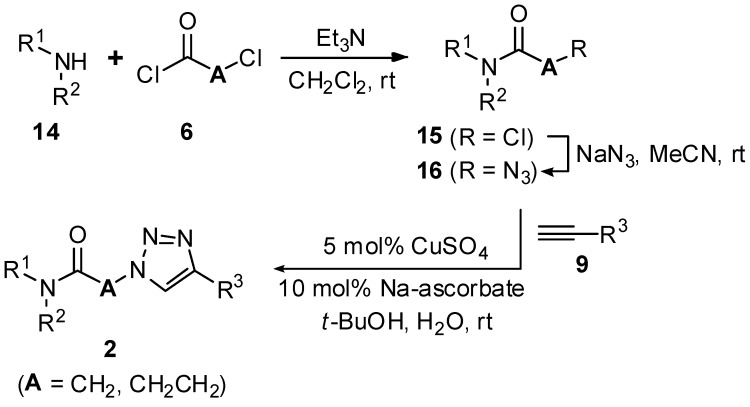
Solution-phase synthesis of tertiary 1,2,3-triazoloamide derivatives **2**.

**Figure 6:**
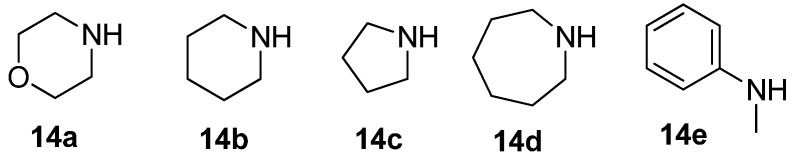
Diversity reagents **14** for tertiary 1,2,3-triazoloamides **2**.

**Figure 7:**
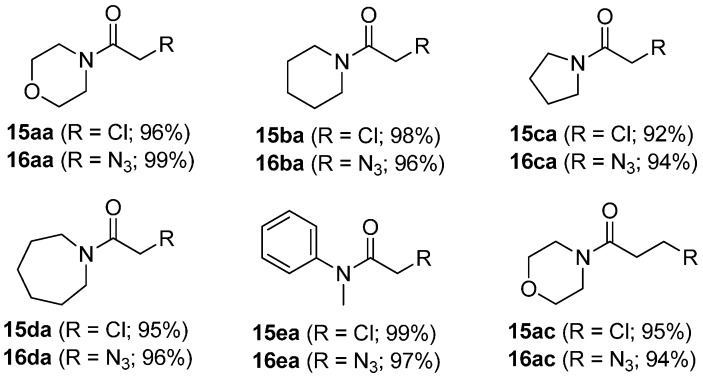
Prepared tertiary amides **15** and sodium azidoamide **16**.

With a successful synthetic route for secondary α-1,2,3-triazoloamides **1**, the stage progressed to the tertiary 1,2,3-triazoloamides **2** (R^2^ ≠ H) (Scheme 4). The chloroamides **15** [31,32,33,34,35,36,37] were prepared from the reaction of secondary amines **14** (the first diversity elements R^1^ and R^2^; Figure 6) and chloro-acid chlorides **6a** and **6c** (the second diversity element **A**; see Figure 3) with triethylamine in CH_2_Cl_2_ at room temperature (99%–92% yields). Followed by S_N_2 reaction of tertiary amides **15** with sodium azide to generated the corresponding azidoamides **16** [15,17,38,39,40,41] in high yields (99%–94% yields) (Figure 7). In contrast to the solid-phase synthesis of secondary 1,2,3-triazoloamides **1**, treatment of tertiary β-chloroamide **15ac** with sodium azide in DMF at room temperature, provided the corresponding β-azidoamide **16ac** in high yield (94% yield) without formation of the undesired acrylamide. Under the general conditions [6,7,8,9,10,24,25,26,27,28] of the Cu-catalyzed 1,3-dipolar cycloaddition (catalytic CuSO_4_/sodium ascorbate) of azidoamide **16aa** and terminal acetylene **9a** in H_2_O/*t*-BuOH, the desired tertiary 1,2,3-triazoloamide **2aaa** is generated in high yield (96%).

By using the parallel solution-phase synthetic route, we were able to prepare a number of tertiary 1,2,3-triazoloamide derivatives **2** displayed in Table 2 starting from appropriate secondary amines **14** (R^1^R^2^NH; Figure 6), chloro-acid chlorides **6a** and **6c** (Cl-**A**-COCl; Figure 3), and terminal acetylenes **9** (R^3^C≡CH; Figure 4). In most cases, tertiary 1,2,3-triazoloamide derivatives **1T** (80 examples) were obtained with high yields (99%–84%) from azidoamide **16** and in high purities, >95% as judged from LC-MS traces (integration of 200–400 nm diode array traces).

**Table 2:** Prepared tertiary 1,2,3-triazoloamide derivatives **2**. ^a^ 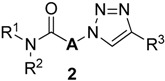

Entry	Products	NR^1^R^2^	A	R^3^	Yield (%) ^b^	Entry	Products	NR^1^R^2^	A	R^3^	Yield (%) ^b^
1	**2aaa**	morpholine	CH_2_	Ph	91	41	**2can**	pyrrolidine	CH_2_	2,4-di-F-Ph	86
2	**2aab**	morpholine	CH_2_	4-MeO-Ph	91	42	**2cao**	pyrrolidine	CH_2_	Bn	81
3	**2aac**	morpholine	CH_2_	4-CN-Ph	88	43	**2daa**	azepine	CH_2_	Ph	87
4	**2aad**	morpholine	CH_2_	3-thiophenyl	87	44	**2dab**	azepine	CH_2_	4-MeO-Ph	78
5	**2aae**	morpholine	CH_2_	2-pyridyl	80	45	**2dac**	azepine	CH_2_	4-CN-Ph	88
6	**2aag**	morpholine	CH_2_	3-MeO-Ph	89	46	**2dad**	azepine	CH_2_	3-thiophenyl	89
7	**2aah**	morpholine	CH_2_	2-MeO-Ph	88	47	**2dae**	azepine	CH_2_	2-pyridyl	78
8	**2aai**	morpholine	CH_2_	4-Me-Ph	86	48	**2dag**	azepine	CH_2_	3-MeO-Ph	84
9	**2aaj**	morpholine	CH_2_	3-Me-Ph	89	49	**2dah**	azepine	CH_2_	2-MeO-Ph	80
10	**2aak**	morpholine	CH_2_	2-Me-Ph	88	50	**2dai**	azepine	CH_2_	4-Me-Ph	88
11	**2aal**	morpholine	CH_2_	4-NMe_2_-Ph	81	51	**2daj**	azepine	CH_2_	3-Me-Ph	83
12	**2aam**	morpholine	CH_2_	4-PhO-Ph	94	52	**2dak**	azepine	CH_2_	2-Me-Ph	78
13	**2aan**	morpholine	CH_2_	2,4-di-F-Ph	87	53	**2dal**	azepine	CH_2_	4-NMe_2_-Ph	82
14	**2aao**	morpholine	CH_2_	Bn	89	54	**2dam**	azepine	CH_2_	4-PhO-Ph	90
15	**2baa**	piperidine	CH_2_	Ph	93	55	**2dan**	azepine	CH_2_	2,4-di-F-Ph	89
16	**2bab**	piperidine	CH_2_	4-MeO-Ph	80	56	**2dao**	azepine	CH_2_	Bn	86
17	**2bac**	piperidine	CH_2_	4-CN-Ph	92	57	**2eaa**	NPhMe	CH_2_	Ph	95
18	**2bad**	piperidine	CH_2_	3-thiophenyl	93	58	**2eab**	NPhMe	CH_2_	4-MeO-Ph	95
19	**2bae**	piperidine	CH_2_	2-pyridyl	95	59	**2eac**	NPhMe	CH_2_	4-CN-Ph	95
20	**2bag**	piperidine	CH_2_	3-MeO-Ph	97	60	**2ead**	NPhMe	CH_2_	3-thiophenyl	85
21	**2bah**	piperidine	CH_2_	2-MeO-Ph	88	61	**2eae**	NPhMe	CH_2_	2-pyridyl	95
22	**2bai**	piperidine	CH_2_	4-Me-Ph	97	62	**2eag**	NPhMe	CH_2_	3-MeO-Ph	89
23	**2baj**	piperidine	CH_2_	3-Me-Ph	93	63	**2eah**	NPhMe	CH_2_	2-MeO-Ph	90
24	**2bak**	piperidine	CH_2_	2-Me-Ph	86	64	**2eai**	NPhMe	CH_2_	4-Me-Ph	95
25	**2bal**	piperidine	CH_2_	4-NMe_2_-Ph	84	65	**2eaj**	NPhMe	CH_2_	3-Me-Ph	93
26	**2bam**	piperidine	CH_2_	4-PhO-Ph	93	66	**2eak**	NPhMe	CH_2_	2-Me-Ph	94
27	**2ban**	piperidine	CH_2_	2,4-di-F-Ph	93	67	**2aca**	morpholine	CH_2_CH_2_	Ph	84
28	**2bao**	piperidine	CH_2_	Bn	89	68	**2acb**	morpholine	CH_2_CH_2_	4-MeO-Ph	80
29	**2caa**	pyrrolidine	CH_2_	Ph	81	69	**2acc**	morpholine	CH_2_CH_2_	4-CN-Ph	80
30	**2cab**	pyrrolidine	CH_2_	4-MeO-Ph	77	70	**2acd**	morpholine	CH_2_CH_2_	3-thiophenyl	88
31	**2cac**	pyrrolidine	CH_2_	4-CN-Ph	81	71	**2ace**	morpholine	CH_2_CH_2_	2-pyridyl	79
32	**2cad**	pyrrolidine	CH_2_	3-thiophenyl	76	72	**2acg**	morpholine	CH_2_CH_2_	3-MeO-Ph	88
33	**2cae**	pyrrolidine	CH_2_	2-pyridyl	75	73	**2ach**	morpholine	CH_2_CH_2_	2-MeO-Ph	88
34	**2cag**	pyrrolidine	CH_2_	3-MeO-Ph	80	74	**2aci**	morpholine	CH_2_CH_2_	4-Me-Ph	83
35	**2cah**	pyrrolidine	CH_2_	2-MeO-Ph	77	75	**2acj**	morpholine	CH_2_CH_2_	3-Me-Ph	88
36	**2cai**	pyrrolidine	CH_2_	4-Me-Ph	83	76	**2ack**	morpholine	CH_2_CH_2_	2-Me-Ph	80
37	**2caj**	pyrrolidine	CH_2_	3-Me-Ph	83	77	**2acl**	morpholine	CH_2_CH_2_	4-NMe_2_-Ph	88
38	**2cak**	pyrrolidine	CH_2_	2-Me-Ph	79	78	**2acm**	morpholine	CH_2_CH_2_	4-PhO-Ph	88
39	**2cal**	pyrrolidine	CH_2_	4-NMe_2_-Ph	81	79	**2acn**	morpholine	CH_2_CH_2_	2,4-di-F-Ph	82
40	**2cam**	pyrrolidine	CH_2_	4-PhO-Ph	86	80	**2aco**	morpholine	CH_2_CH_2_	Bn	88

^a^ All reactions were performed on 0.1 mmol scale of **16** and the purities of compounds **2** were over 95% as judged from LC-MS traces (integration of diode array 200–400 nm traces); ^b^ Three-step overall yield from secondary amine **14**.

## 3. Experimental Section

### 3.1. General

All chemicals were reagent grade and used as purchased. The Merrifield resin (loading capacity 1.29 mmol/g, 100–200 mesh) was purchased from BeadTech (Seoul, Korea). Reactions were monitored by TLC analysis using silica gel 60 F-254 thin layer plates (Merck, Darmstadt, Germany) or ATR-FRIR analysis using a Cary 630 instrument (Agilent Technologies, Santa Clara, CA, USA). Flash column chromatography was carried out on Merck silica gel 60 (230–400 mesh). The crude products were purified by parallel chromatography using CombiFlash (Isco, Lincoln, NE, USA). ^1^H-NMR (500 MHz) and ^13^C-NMR (125 MHz) spectra were recorded in δ units relative to deuterated solvent (CDCl_3_, DMSO-*d*_6_, *etc.*) as internal reference on a 500 MHz NMR instrument (Bruker, Billerca, MA, USA). LC-MS analysis was performed on ESI mass spectrometer with PDA detection. LC-MS area% purities of all products were determined by LC peak area analysis (XBD C18 column, 4.6 mm × 100 mm; PDA detector at 200–400 nm; isocratic, 5 mM ammonium formate/CH_3_CN (30:70)).

### 3.2. General Procedure for the Preparation of Secondary α-1,2,3-Triazoloamides ***1*** on Solid-Phase

A typical procedure for the desired secondary α-1,2,3-triazoloamides **1**, as exemplified for *N*-phenyl-2-(4-phenyl-1*H*-1,2,3-triazol-1-yl)acetamide (**1aaa**; R^1^ = R^3^ = Ph, **A** = CH_2_) follows.

#### 3.2.1. Preparation of AMEBA Resin **4**

Merrifield resin (53.2 g, 50.0 mmol, 0.94 mmol/g) was treated with 4-formyl-3-methoxyphenol (22.8 g, 150.0 mmol), potassium iodide (83.0 mg, 0.5 mmol), and potassium carbonate (20.7 g, 150.0 mmol) in DMF (300 mL). The mixture was shaken at room temperature for 10 h, and then filtered, washed several times with H_2_O, DMF, MeOH, and CH_2_Cl_2_, and dried in a vacuo to give AMEBA resin **4** (59.0 g): On-bead ATR-FTIR (neat) υ_max_ 1678, 1598, 1259 (cm^−1^).

#### 3.2.2. Preparation of Secondary Amine Resin **3**

A mixture of AMEBA resin **4** (10 g, theoretically 8.5 mmol), aniline (**5a**; (2.3 mL, 25.5 mmol), sodium triacetoxyborohydride (5.4 g, 25.5 mmol), and acetic acid (0.49 mL, 8.5 mmol) in 1,2-dichloroethane was heated at 50 °C for 12 h. The reaction mixture was cooled to room temperature, and then filtered, washed several times with H_2_O, DMF, MeOH, and CH_2_Cl_2_, and dried in a vacuum oven to give secondary amine resin **3a** (10.6 g): On-bead ATR-FTIR (neat) υ_max_ 3424, 1597, 1489 (cm^−1^).

#### 3.2.3. Preparation of α-Chloroamide Resin **7**

The amine resin **3a** (3.0 g, theoretically 2.4 mmol) was treated with 2-chloroacetyl chloride (**6a**; 0.57 mL, 7.2 mmol) and triethylamine (1.0 mL, 7.2 mmol) in CH_2_Cl_2_ at 0 °C. The reaction mixture was shaken at room temperature for 5 h, and then filtered, washed several times with H_2_O, DMF, MeOH, and CH_2_Cl_2_, and dried in a vacuum oven to give α-chloroamide resin **7aa** (3.16 g): On-bead ATR-FTIR (neat) υ_max_ 1666, 1593, 1489 (cm^−1^).

#### 3.2.4. Preparation of α-Azidoamide Resin **8**

The α-chloroamide resin **7aa** (2.8 g, theoretically 2.1 mmol) was treated with sodium azide (0.47 g, 7.2 mmol) in DMF. The reaction mixture was shaken at room temperature for 12 h, and then filtered, washed several times with H_2_O, DMF, MeOH, and CH_2_Cl_2_, and dried in a vacuum oven to give α-azidoamide resin **8aa** (2.8 g): On-bead ATR-FTIR (neat) υ_max_ 2101, 1670, 1590, 1491 (cm^−1^).

#### 3.2.5. Preparation of α-1,2,3-Triazoloamide Resin **10**

To a mixture of α-azidoamide resin **8aa** (570 mg, theoretically 0.42 mmol) and phenylacetylene (**9a**, 0.07 mL, 0.6 mmol) in DMF/piperidine (4:1) was added copper(I) iodide (229 mg, 1.27 mmol) and sodium ascorbate (57 mg, 1.27 mmol) at room temperature. The reaction mixture was shaken at room temperature for 12 h, and then filtered, washed several times with H_2_O, DMF, MeOH, and CH_2_Cl_2_, and dried in a vacuum oven to give α-1,2,3-triazoloamide resin **10aaa** (606 mg): On-bead ATR-FTIR (neat) υ_max_ 1669, 1590, 1489 (cm^−1^).

#### 3.2.6. Preparation of α-1,2,3-Triazoloamide **1**

The α-1,2,3-triazoloamide resin **10aaa** (157 mg, theoretically 0.10 mmol) was added 30% TFA in CH_2_Cl_2_ (3 mL). The reaction mixture was stirred at 45 °C for 1day and the mixture was filtered and washed with CH_2_Cl_2_ and MeOH. The filtrate was evaporated in vacuo and the residue was dissolved in CH_2_Cl_2_ and extracted with saturated NaHCO_3_. The aqueous layer was extracted with CH_2_Cl_2_ twice and the combined organic extracts were washed with brine, dried over MgSO_4_, filtered and concentrated in vacuo to give the target *N*-phenyl-2-(4-phenyl-1*H*-1,2,3-triazol-1-yl)acetamide (**1aaa**) was obtained as a light yellow solid (23 mg, 93% from Merrifield resin). Mp 247–249 °C; ^1^H-NMR (DMSO-*d*_6_) δ 5.39 (s, 2H), 7.09 (m, 1H), 7.33–7.36 (m, 3H), 7.46 (m, 2H), 7.60 (m, 2H), 7.88 (m, 2H), 8.60 (s, 1H), 10.51 (s, 1H); ^13^C-NMR (DMSO-*d*_6_) δ 52.4, 119.3, 123.1, 123.8, 125.1, 127.9, 128.9, 130.7, 138.4, 146.2, 164.2; IR (ATR) υ_max_ 3273, 3061, 1677, 1601, 1545, 1442, 1365, 1253, 1202, 1080, 753 (cm^−1^); LC-MS (ESI) *m/z* 279 ([M + 1]^+^); HRMS (FAB) calcd for C_16_H_15_N_4_O ([M + H]^+^) 279.1240, found 279.1239.

### 3.3. Characterization Data of Secondary α-1,2,3-Triazoloamides ***1***

*2-[4-(4-Methoxyphenyl)-1H-1,2,3-triazol-1-yl]-N-phenylacetamide* (**1aab**). White solid; Yield: 91%. ^1^H-NMR (DMSO-*d*_6_) δ 3.79 (s, 3H), 5.36 (s, 2H), 7.03 (d, *J* = 8.9 Hz, 2H), 7.09 (m, 1H), 7.35 (m, 2H), 7.60 (m, 2H), 7.80 (d, *J* = 8.8 Hz, 2H), 8.48 (s, 1H), 10.50 (s, 1H); ^13^C-NMR (DMSO-*d*_6_) δ 52.3, 55.2, 114.4, 119.3, 122.1, 123.3, 123.8, 126.5, 128.9, 138.4, 146.2, 159.0, 164.2; LC-MS (ESI) *m/z* 309 ([M + 1]^+^).

*2-[4-(4-Cyanophenyl)-1H-1,2,3-triazol-1-yl]-N-phenylacetamide* (**1aac**). Light brown solid; Yield: 83%. ^1^H-NMR (DMSO-*d*_6_) δ 5.43 (s, 2H), 7.09 (m, 1H), 7.34 (m, 2H), 7.59 (m, 2H), 7.93 (d, *J* = 8.6 Hz, 2H), 8.09 (d, *J* = 8.5 Hz, 2H), 8.82 (s, 1H), 10.53 (s, 1H); ^13^C-NMR (DMSO-*d*_6_) δ 52.5, 110.1, 118.8, 119.2, 123.8, 124.8, 125.7, 128.9, 133.0, 135.2, 138.4, 144.7, 164.0; LC-MS (ESI) *m/z* 304 ([M + 1]^+^).

*N-Phenyl-2-[4-(thiophen-3-yl)-1H-1,2,3-triazol-1-yl]acetamide* (**1aad**). White solid; Yield: 82%. ^1^H-NMR (DMSO-*d*_6_) δ 5.37 (s, 2H), 7.09 (m, 1H), 7.34 (m, 2H), 7.55 (dd, *J* = 1.2, 5.0 Hz, 1H), 7.59 (m, 2H), 7.66 (dd, *J* = 3.0, 5.0 Hz, 1H), 7.88 (dd, *J* = 1.2, 2.9 Hz, 1H), 8.46 (s, 1H), 10.50 (s, 1H); ^13^C-NMR (DMSO-*d*_6_) δ 52.3, 119.2, 120.8, 122.8, 123.8, 125.8, 127.1, 128.9, 132.0, 138.4, 142.8, 164.2; LC-MS (ESI) *m/z* 285 ([M + 1]^+^).

*N-Phenyl-2-[4-(pyridin-2-yl)-1H-1,2,3-triazol-1-yl]acetamide* (**1aae**). White solid; Yield: 85%. ^1^H-NMR (DMSO-*d*_6_) δ 5.42 (s, 2H), 7.09 (m, 1H), 7.33–7.36 (m, 3H), 7.59 (m, 2H), 7.91 (t, *J* = 7.7 Hz, 1H), 8.06 (d, *J* = 7.6 Hz, 1H), 8.62 (s, 2H), 10.51 (s, 1H); ^13^C-NMR (DMSO-*d*_6_) δ 52.3, 119.2, 119.4, 123.0, 123.8, 125.0, 128.9, 137.2, 138.4, 147.1, 149.7, 150.0, 164.1; LC-MS (ESI) *m/z* 280 ([M + 1]^+^).

*2-(4-Butyl-1H-1,2,3-triazol-1-yl)-N-phenylacetamide* (**1aaf**). White solid; Yield: 92%. ^1^H-NMR (DMSO-*d*_6_) δ 0.90 (t, *J* = 7.3 Hz, 3H), 1.34 (m, 2H), 1.59 (m, 2H), 2.64 (m, 2H), 5.26 (s, 2H), 7.08 (m, 1H), 7.33 (m, 2H), 7.58 (m, 2H), 7.86 (s, 1H), 10.43 (s, 1H); ^13^C-NMR (DMSO-*d*_6_) δ 13.7, 21.7, 24.6, 31.1, 52.1, 119.2, 123.4, 123.7, 128.9, 138.4, 146.7, 164.4; LC-MS (ESI) *m/z* 259 ([M + 1]^+^).

*N-(4-Methoxyphenyl)-2-(4-phenyl-1H-1,2,3-triazol-1-yl)acetamide* (**1baa**). White solid; Yield: 92%. ^1^H-NMR (DMSO-*d*_6_) δ 3.72 (s, 3H), 5.34 (s, 2H), 6.91 (d, *J* = 9.1 Hz, 2H), 7.34 (m, 1H), 7.44–7.47 (m, 2H), 7.51 (d, *J* = 9.1 Hz, 2H), 7.87-7.88 (m, 2H), 8.59 (s, 1H), 10.37 (s, 1H); ^13^C-NMR (DMSO-*d*_6_) δ 52.3, 55.2, 114.1, 120.9, 123.1, 125.2, 127.9, 129.0, 130.7, 131.5, 146.2, 155.6, 163.7; LC-MS (ESI) *m/z* 309 ([M + 1]^+^).

*N-(4-Methoxyphenyl)-2-[4-(4-methoxyphenyl)-1H-1,2,3-triazol-1-yl]acetamide* (**1bab**). Yellow solid; Yield: 94%. ^1^H-NMR (DMSO-*d*_6_) δ 3.72 (s, 3H), 3.79 (s, 3H), 5.31 (s, 2H), 6.91 (d, *J* = 9.1 Hz, 2H), 7.02 (d, *J* = 8.9 Hz, 2H), 7.51 (d, *J* = 9.1 Hz, 2H), 7.79 (d, *J* = 8.9 Hz, 2H), 8.47 (s, 1H), 10.36 (s, 1H); ^13^C-NMR (DMSO-*d*_6_) δ 52.3, 55.18, 55.21, 114.1, 114.4, 120.9, 122.1, 123.4, 126.5, 131.5, 155.6, 158.0, 159.0, 163.7; LC-MS (ESI) *m/z* 339 ([M + 1]^+^).

*2-[4-(4-Cyanophenyl)-1H-1,2,3-triazol-1-yl]-N-(4-methoxyphenyl)acetamide* (**1bac**). Yellow solid; Yield: 89%. ^1^H-NMR (DMSO-*d*_6_) δ 3.72 (s, 3H), 5.38 (s, 2H), 6.91 (d, *J* = 9.1 Hz, 2H), 7.50 (d, *J* = 9.1 Hz, 2H), 7.93 (d, *J* = 8.6 Hz, 2H), 8.08 (d, *J* = 8.6 Hz, 2H), 8.81 (s, 1H), 10.39 (s, 1H); ^13^C-NMR (DMSO-*d*_6_) δ 52.9, 55.7, 110.6, 114.5, 119.3, 121.3, 125.2, 126.2, 131.9, 133.5, 135.6, 145.1, 156.1, 163.9; LC-MS (ESI) *m/z* 334 ([M + 1]^+^).

*N-(4-Methoxyphenyl)-2-[4-(thiophen-3-yl)-1H-1,2,3-triazol-1-yl]acetamide* (**1bad**). Light brown solid; Yield: 85%. ^1^H-NMR (DMSO-*d*_6_) δ 3.72 (s, 3H), 5.33 (s, 2H), 6.91 (d, *J* = 9.1 Hz, 2H), 7.51 (d, *J* = 9.1 Hz, 2H) 7.55 (dd, *J* = 1.3, 5.0 Hz, 1H), 7.65 (dd, *J* = 3.0, 5.0 Hz, 1H), 7.88 (dd, *J* = 1.2, 2.9 Hz, 1H), 8.45 (s, 1H), 10.36 (s, 1H); ^13^C-NMR (DMSO-*d*_6_) δ 52.2, 55.2, 114.0, 120.8, 122.7, 125.8, 127.1, 131.5, 132.0, 142.8, 155.6, 163.6; LC-MS (ESI) *m/z* 315 ([M + 1]^+^).

*N-(4-Methoxyphenyl)-2-[4-(3-methoxyphenyl)-1H-1,2,3-triazol-1-yl]acetamide* (**1bag**). White solid; Yield: 87%. ^1^H-NMR (CDCl_3_) δ 3.78 (s, 3H), 3.88 (s, 3H), 5.23 (s, 2H), 6.85 (d, *J* = 9.1 Hz, 2H), 6.93 (ddd, *J* = 1.3, 2.5, 7.9 Hz, 1H), 7.34–7.40 (m, 4H), 7.46 (m, 1H), 7.78 (br s, 1H), 7.97 (s, 1H); ^13^C-NMR (DMSO-*d*_6_) δ 52.3, 55.1, 55.2, 110.3, 113.6, 114.0, 117.5, 120.8, 123.2, 130.0, 131.5, 132.1, 146.1, 155.6, 159.7, 163.6; LC-MS (ESI) *m/z* 339 ([M + 1]^+^).

*N-(4-Methoxyphenyl)-2-(4-m-tolyl-1H-1,2,3-triazol-1-yl)acetamide* (**1baj**). White solid; Yield: 89%. ^1^H-NMR (CDCl_3_) δ 2.42 (s, 3H), 3.78 (s, 3H), 5.22 (s, 2H), 6.85 (d, *J* = 9.1 Hz, 2H), 7.19 (d, *J* = 7.6 Hz, 1H), 7.34 (t, *J* = 7.7 Hz, 1H), 7.36 (d, *J* = 9.1 Hz, 2H), 7.63 (d, *J* = 7.7 Hz, 1H), 7.71 (s, 1H), 7.83 (br s, 1H), 7.96 (s, 1H); ^13^C-NMR (DMSO-*d*_6_) δ 21.0, 52.3, 55.2, 114.0, 120.8, 122.3, 122.9, 125.7, 128.5, 128.8, 130.6, 131.5, 138.0, 146.3, 155.6, 163.6;LC-MS (ESI) *m/z* 323 ([M + 1]^+^).

*N-(4-Methoxyphenyl)-2-(4-o-tolyl-1H-1,2,3-triazol-1-yl)acetamide* (**1bak**). Light yellow solid; Yield: 84%. ^1^H-NMR (CDCl_3_) δ 2.50 (s, 3H), 3.79 (s, 3H), 5.25 (s, 2H), 6.86 (d, *J* = 9.1 Hz, 2H), 7.30–7.32 (m, 3H), 7.38 (d, *J* = 9.0 Hz, 2H), 7.80 (m, 1H), 7.86 (br s, 1H), 7.87 (s, 1H); ^13^C-NMR (DMSO-*d*_6_) δ 21.1, 52.2, 55.2, 114.0, 120.8, 124.9, 126.0, 127.8, 128.1, 130.0, 130.9, 131.5, 134.8, 145.3, 155.6, 163.7; LC-MS (ESI) *m/z* 323 ([M + 1]^+^).

*2-{4-[4-(Dimethylamino)phenyl]-1H-1,2,3-triazol-1-yl}-N-(4-methoxyphenyl)acetamide* (**1bal**). Brown solid; Yield: 79%. ^1^H-NMR (DMSO-*d*_6_) δ 2.94 (s, 6H), 3.72 (s, 3H), 5.32 (s, 2H), 6.68 (dd, *J* = 1.9, 8.3 Hz, 1H), 6.79 (d, *J* = 9.0 Hz, 2H), 7.11 (dd, *J* = 1.0, 8.1 Hz, 1H), 7.24 (t, *J* = 8.2 Hz, 1H), 7.30 (t, *J* = 2.1 Hz, 1H), 7.67 (d, *J* = 8.9 Hz, 2H), 8.36 (s, 1H), 10.48 (s, 1H); ^13^C-NMR (DMSO-*d*_6_) δ 40.1, 52.2, 55.2, 112.4, 114.1, 118.7, 121.2, 126.1, 131.5, 146.9, 150.1, 155.6, 163.8; LC-MS (ESI) *m/z* 352 ([M + 1]^+^).

*N-(4-Methoxyphenyl)-2-[4-(4-phenoxyphenyl)-1H-1,2,3-triazol-1-yl]acetamide* (**1bam**). Light yellow solid; Yield: 92%. ^1^H-NMR (DMSO-*d*_6_) δ 3.72 (s, 3H), 5.34 (s, 2H), 6.91 (d, *J* = 9.1 Hz, 2H), 7.06–7.10 (m, 4H), 7.17 (m, 1H), 7.40–7.44 (m, 2H), 7.51 (d, *J* = 9.1 Hz, 2H), 7.88 (d, *J* = 8.8 Hz, 2H), 8.55 (s, 1H), 10.37 (s, 1H); ^13^C-NMR (DMSO-*d*_6_) δ 52.3, 55.2, 114.1, 118.9, 119.0, 120.9, 122.7, 123.7,126.1,127.0, 130.2, 131.5, 145.8, 155.6, 156.5, 163.7; LC-MS (ESI) *m/z* 401 ([M + 1]^+^).

*2-[4-(2,4-Difluorophenyl)-1H-1,2,3-triazol-1-yl]-N-(4-methoxyphenyl)acetamide* (**1ban**). Light yellow solid; Yield: 87%. ^1^H-NMR (CDCl_3_) δ 3.78 (s, 3H), 5.24 (s, 2H), 6.85 (d, *J* = 9.1 Hz, 2H), 6.93 (ddd, *J* = 2.4, 8.6, 11.0 Hz, 1H), 7.03 (m, 1H), 7.36 (d, *J* = 9.1 Hz, 2H), 7.70 (br s, 1H), 8.10 (d, *J* = 3.6 Hz, 1H), 8.30 (dt, *J* = 6.5, 8.6 Hz, 1H); ^13^C-NMR (DMSO-*d*_6_) δ 52.2, 55.2, 104.6 (t, *J*_CF_ = 26.0 Hz), 112.3 (dd, *J*_CF_ = 21.2, 3.5 Hz), 115.2 (dd, *J*_CF_ = 13.3, 3.6 Hz), 114.0, 125.2 (d, *J*_CF_ = 10.8 Hz), 120.8, 128.5 (dd, *J*_CF_ = 9.8, 5.1 Hz), 131.5, 138.8 (d, *J*_CF_ = 2.6 Hz), 155.5, 158.5 (dd, *J*_CF_ = 250.3, 12.7 Hz), 161.7 (dd, *J*_CF_ = 247.4, 12.7 Hz), 163.6; LC-MS (ESI) *m/z* 345 ([M + 1]^+^).

*2-(4-Benzyl-1H-1,2,3-triazol-1-yl)-N-(4-methoxyphenyl)acetamide* (**1bao**). White solid; Yield: 85%. ^1^H-NMR (CDCl_3_) δ 3.78 (s, 3H), 4.13 (s, 2H), 5.09 (s, 2H), 6.84 (d, *J* = 9.0 Hz, 2H), 7.22–7.34 (m, 7H), 7.37 (s, 1H), 7.81 (br s, 1H); ^13^C-NMR (DMSO-*d*_6_) δ 31.3, 52.1, 55.2, 114.0, 120.8, 124.2, 126.2, 128.4, 128.6, 131.5, 139.6, 145.9, 155.6, 163.8; LC-MS (ESI) *m/z* 323 ([M + 1]^+^).

*N-(3-Methoxyphenyl)-2-(4-phenyl-1H-1,2,3-triazol-1-yl)acetamide* (**1caa**). Light yellow solid; Yield: 91%. ^1^H-NMR (CDCl_3_) δ 3.79 (s, 3H), 5.24 (s, 2H), 6.70 (dd, *J* = 2.2, 8.1 Hz, 1H), 6.97 (dd, *J* = 1.5, 7.9 Hz, 1H), 7.19–7.23 (m, 2H), 7.37 (m, 1H), 7.44–7.47 (m, 2H), 7.85–7.87 (m, 2H), 7.95 (s, 1H), 7.98 (s, 1H); ^13^C-NMR (DMSO-*d*_6_) δ 52.4, 55.0, 105.0, 109.3, 111.5, 123.0, 125.1, 127.8, 128.9, 129.7, 130.7,139.5, 146.2, 159.6, 164.2; LC-MS (ESI) *m/z* 309 ([M + 1]^+^).

*N-(3-Methoxyphenyl)-2-[4-(4-methoxyphenyl)-1H-1,2,3-triazol-1-yl]acetamide* (**1cab**). White solid; Yield: 90%. ^1^H-NMR (CDCl_3_) δ 3.79 (s, 3H), 3.86 (s, 3H), 5.22 (s, 2H), 6.70 (dd, *J* = 2.3, 7.9 Hz, 1H), 6.96–6.99 (m, 3H), 7.18 (t, *J* = 2.2 Hz, 1H), 7.21 (t, *J* = 8.2 Hz, 1H), 7.78 (d, *J* = 8.9 Hz, 2H), 7.88 (s, 1H), 7.91 (s, 1H); ^13^C-NMR (DMSO-*d*_6_) δ 52.3, 55.0, 55.1, 105.0, 109.3, 111.5, 114.3, 122.1, 123.3, 126.5, 129.7, 139.6, 146.1, 159.0, 159.6, 164.3; LC-MS (ESI) *m/z* 339 ([M + 1]^+^).

*2-[4-(4-Cyanophenyl)-1H-1,2,3-triazol-1-yl]-N-(3-methoxyphenyl)acetamide* (**1cac**). White solid; Yield: 87%. ^1^H-NMR (DMSO-*d*_6_) δ 3.72 (s, 3H), 5.41 (s, 2H), 6.68 (dd, *J* = 1.9, 8.3 Hz, 1H), 7.11 (d, *J* = 8.1 Hz, 1H), 7.25 (t, *J* = 8.2 Hz, 1H), 7.29 (t, *J* = 2.1 Hz, 1H), 7.93 (d, *J* = 8.6 Hz, 2H), 8.08 (d, *J* = 8.6 Hz, 2H), 8.81 (s, 1H), 10.52 (s, 1H); ^13^C-NMR (DMSO-*d*_6_) δ 52.5, 55.0, 105.0, 109.3, 110.1, 111.5, 124.7, 125.7, 129.7, 133.0, 135.2, 139.5, 144.6, 159.6, 164.0; LC-MS (ESI) *m/z* 334 ([M + 1]^+^).

*N-(3-Methoxyphenyl)-2-[4-(thiophen-3-yl)-1H-1,2,3-triazol-1-yl]acetamide* (**1cad**). Light brown solid; Yield: 76%. ^1^H-NMR (DMSO-*d*_6_) δ 3.72 (s, 3H), 5.36 (s, 2H), 6.68 (dd, *J* = 2.2, 7.9 Hz, 1H), 7.11 (dd, *J* = 0.9, 8.1 Hz, 1H), 7.24 (t, *J* = 8.2 Hz, 1H), 7.30 (t, *J* = 2.1 Hz, 1H), 7.55 (dd, *J* = 1.3, 5.0 Hz, 1H), 7.66 (dd, *J* = 3.0, 5.0 Hz, 1H), 7.88 (dd, *J* = 1.2, 3.0 Hz, 1H), 8.46 (s, 1H), 10.50 (s, 1H); ^13^C-NMR (DMSO-*d*_6_) δ 52.3, 55.0, 105.0, 109.3, 111.5, 120.8, 122.8, 125.8, 127.1, 129.7, 132.0, 139.5, 142.8, 159.6, 164.2; LC-MS (ESI) *m/z* 315 ([M + 1]^+^).

*N-(3-Methoxyphenyl)-2-[4-(pyridin-2-yl)-1H-1,2,3-triazol-1-yl]acetamide* (**1cae**). Brown solid; Yield: 81%. ^1^H-NMR (CDCl_3_) δ 3.79 (s, 3H), 5.26 (s, 2H), 6.70 (dd, *J* = 2.1, 8.2 Hz, 1H), 6.95 (d, *J* = 8.0 Hz, 1H), 7.18–7.22 (m, 2H), 7.28 (m, 1H), 7.82 (t, *J* = 7.6 Hz, 1H), 7.87 (s, 1H), 8.19 (d, *J* = 7.4 Hz, 1H), 8.33 (s, 1H), 8.61 (br s, 1H); ^13^C-NMR (DMSO-*d*_6_) δ 52.4, 55.1, 105.1, 109.4, 115.6, 119.5, 123.1, 125.1, 129.9, 137.4, 139.6, 147.1, 149.8, 150.0, 159.7, 164.3; LC-MS (ESI) *m/z* 310 ([M + 1]^+^).

*N-(3-Methoxyphenyl)-2-[4-(2-methoxyphenyl)-1H-1,2,3-triazol-1-yl]acetamide* (**1cah**). Light yellow solid; Yield: 83%. ^1^H-NMR (CDCl_3_) δ 3.78 (s, 3H), 3.96 (s, 3H), 5.24 (s, 2H), 6.96 (dd, *J* = 1.3, 8.1 Hz, 1H), 7.01 (d, *J* = 8.3 Hz, 1H), 7.11 (dt, *J* = 0.9, 7.6 Hz, 1H), 7.17 (t, *J* = 2.2 Hz, 1H), 7.20 (t, *J* = 8.2 Hz, 1H), 7.36 (m, 1H), 7.92 (br s, 1H), 8.24 (s, 1H), 8.36 (dd, *J* = 1.7, 7.8 Hz, 1H); ^13^C-NMR (DMSO-*d*_6_) δ 52.2, 55.0, 55.5, 105.0, 109.2, 111.4, 111.5, 119.1, 120.6, 125.6, 126.5, 128.8, 129.7, 139.6, 141.6, 155.3, 159.5, 164.4; LC-MS (ESI) *m/z* 339 ([M + 1]^+^).

*N-(3-Methoxyphenyl)-2-(4-m-tolyl-1H-1,2,3-triazol-1-yl)acetamide* (**1caj**). White solid; Yield: 93%. ^1^H-NMR (CDCl_3_) δ 2.42 (s, 3H), 3.79 (s, 3H), 5.23 (s, 2H), 6.70 (dd, *J* = 2.2, 8.1 Hz, 1H), 6.97 (dd, *J* = 1.3, 8.0 Hz, 1H), 7.19–7.23 (m, 3H), 7.34 (t, *J* = 7.7 Hz, 1H), 7.63 (d, *J* = 7.6 Hz, 1H), 7.71 (s, 1H), 7.94 (br s, 1H), 7.96 (s, 1H); ^13^C-NMR (DMSO-*d*_6_) δ 52.4, 55.0, 55.1, 105.0, 109.3, 110.3, 111.5, 113.6, 117.5, 123.7, 129.7, 130.0, 132.0, 139.5, 146.1, 159.6, 159.7, 164.2; LC-MS (ESI) *m/z* 323 ([M + 1]^+^).

*N-(3-Methoxyphenyl)-2-(4-o-tolyl-1H-1,2,3-triazol-1-yl)acetamide* (**1cak**). Light yellow solid; Yield: 90%. ^1^H-NMR (CDCl_3_) δ 2.50 (s, 3H), 3.79 (s, 3H), 5.26 (s, 2H), 6.71 (dd, *J* = 2.1, 8.3 Hz, 1H), 6.98 (dd, *J* = 1.3, 8.0 Hz, 1H), 7.19–7.24 (m, 2H), 7.28-7.32 (m, 3H), 7.79 (m, 1H), 7.87 (s, 1H), 8.00 (br s, 1H); ^13^C-NMR (DMSO-*d*_6_) δ 21.1, 52.3, 55.0, 105.0, 109.3, 111.5, 124.9, 126.1, 127.8, 128.1, 129.7, 123.0, 130.9, 134.8, 139.6, 145.4, 159.6, 164.3; LC-MS (ESI) *m/z* 323 ([M + 1]^+^).

*2-{4-[4-(Dimethylamino)phenyl]-1H-1,2,3-triazol-1-yl}-N-(3-methoxyphenyl)acetamide* (**1cal**). Brown solid; Yield: 91%. ^1^H-NMR (DMSO-*d*_6_) δ 2.94 (s, 6H), 3.72 (s, 3H), 5.32 (s, 2H), 6.68 (dd, *J* = 1.9, 8.3 Hz, 1H), 6.79 (d, *J* = 9.0 Hz, 2H), 7.11 (dd, *J* = 1.0, 8.1 Hz, 1H), 7.24 (t, *J* = 8.2 Hz, 1H), 7.30 (t, *J* = 2.1 Hz, 1H), 7.67 (d, *J* = 8.9 Hz, 2H), 8.36 (s, 1H), 10.48 (s, 1H); ^13^C-NMR (DMSO-*d*_6_) δ 40.0, 52.3, 55.0, 105.0, 109.3, 111.5, 112.4, 118.6, 121.2, 126.1, 129.7, 139.6, 146.8, 150.1, 159.6, 164.3; LC-MS (ESI) *m/z* 352 ([M + 1]^+^).

*N-(3-Methoxyphenyl)-2-[4-(4-phenoxyphenyl)-1H-1,2,3-triazol-1-yl]acetamide* (**1cam**). Yellow solid; Yield: 91%. ^1^H-NMR (DMSO-*d*_6_) δ 3.72 (s, 3H), 5.37 (s, 2H), 6.68 (dd, *J* = 1.8, 8.3 Hz, 1H), 7.06–7.10 (m, 4H), 7.12 (dd, *J* = 1.0, 8.1 Hz, 1H), 7.17 (m, 1H), 7.25 (t, *J* = 8.2 Hz, 1H), 7.30 (t, *J* = 2.2 Hz, 1H), 7.40–7.44 (m, 2H), 7.88 (d, *J* = 8.8 Hz, 2H), 8.55 (s, 1H), 10.50 (s, 1H); ^13^C-NMR (DMSO-*d*_6_) δ 52.4, 55.0, 105.0, 109.3, 111.5, 118.8, 118.9, 122.7, 123.7, 126.0, 126.9, 129.7, 130.1, 139.5, 145.7, 156.4, 159.6, 164.2; LC-MS (ESI) *m/z* 401 ([M + 1]^+^).

*2-[4-(2,4-Difluorophenyl)-1H-1,2,3-triazol-1-yl]-N-(3-methoxyphenyl)acetamide* (**1can**). Light yellow solid; Yield: 92%. ^1^H-NMR (CDCl_3_) δ 3.79 (s, 3H), 5.25 (s, 2H), 6.70 (dd, *J* = 2.0, 8.4 Hz, 1H), 6.93 (ddd, *J* = 2.5, 8.7, 11.1 Hz, 1H), 6.95 (dd, *J* = 1.7, 7.7 Hz, 1H), 7.03 (m, 1H), 7.19 (t, *J* = 2.2 Hz, 1H), 7.22 (t, *J* = 8.2 Hz, 1H), 7.81 (br s, 1H), 8.10 (d, *J* = 3.5 Hz, 1H), 8.30 (dt, *J* = 6.5, 8.6 Hz, 1H); ^13^C-NMR (DMSO-*d*_6_) δ 52.3, 55.0, 104.6 (t, *J*_CF_ = 26.1 Hz), 105.0, 109.27, 111.4, 112.3 (dd, *J*_CF_ = 21.4, 3.5 Hz), 115.2 (dd, *J*_CF_ = 13.4, 3.7 Hz), 125.2 (d, *J*_CF_ = 10.9 Hz), 128.5 (dd, *J*_CF_ = 9.8, 5.3 Hz), 129.7, 138.9 (d, *J*_CF_ = 2.6 Hz), 139.6, 158.5 (dd, *J*_CF_ = 250.7, 12.8), 159.6, 161.7 (dd, *J*_CF_ = 247.5, 12.5 Hz), 164.2; LC-MS (ESI) *m/z* 345 ([M + 1]^+^).

*2-(4-Benzyl-1H-1,2,3-triazol-1-yl)-N-(3-methoxyphenyl)acetamide* (**1cao**). White solid; Yield: 88%. ^1^H-NMR (CDCl_3_) δ 3.77 (s, 3H), 4.12 (s, 2H), 5.10 (s, 2H), 6.68 (dd, *J* = 2.1, 8.2 Hz, 1H), 6.94 (dd, *J* = 1.1, 7.9 Hz, 1H), 7.17–7.32 (m, 7H), 7.39 (s, 1H), 8.15 (br s, 1H); ^13^C-NMR (DMSO-*d*_6_) δ 31.2, 52.1, 55.0, 105.0, 109.2, 111.4, 124.1, 126.1, 128.4, 128.5, 129.7, 139.56, 139.59, 145.9, 159.5, 164.3; LC-MS (ESI) *m/z* 323 ([M + 1]^+^).

*N-(2-Methoxyphenyl)-2-(4-phenyl-1H-1,2,3-triazol-1-yl)acetamide* (**1daa**). Light yellow solid; Yield: 94%. ^1^H-NMR (CDCl_3_) δ 3.80 (s, 3H), 5.27 (s, 2H), 6.85 (d, *J* = 8.2 Hz, 1H), 6.96 (dt, *J* = 1.1, 7.8 Hz, 1H), 7.08 (dt, *J* = 1.4, 7.9 Hz, 1H), 7.37 (m, 1H), 7.44–7.47 (m, 2H), 7.86–7.88 (m, 2H), 7.99 (s, 1H), 8.24 (s, 1H), 8.27 (dd, *J* = 1.4, 8.1 Hz, 1H); ^13^C-NMR (DMSO-*d*_6_) δ 52.3, 55.7, 111.3, 120.3, 121.7, 123.0, 124.9, 125.1, 126.6, 127.8, 128.9, 130.7, 146.2, 149.6, 164.4; LC-MS (ESI) *m/z* 309 ([M + 1]^+^).

*N-(2-Methoxyphenyl)-2-[4-(4-methoxyphenyl)-1H-1,2,3-triazol-1-yl]acetamide* (**1dab**). White solid; Yield: 90%. ^1^H-NMR (CDCl_3_) δ 3.79 (s, 3H), 3.85 (s, 3H), 5.25 (s, 2H), 6.84 (dd, *J* = 1.0, 8.2 Hz, 1H), 6.95 (dt, *J* = 1.1, 7.7 Hz, 1H), 6.98 (d, *J* = 8.9 Hz, 2H), 7.07 (dt, *J* = 1.5, 7.9 Hz, 1H), 7.79 (d, *J* = 8.9 Hz, 2H), 7.90 (s, 1H), 8.24 (s, 1H), 8.27 (dd, *J* = 1.5, 8.1 Hz, 1H); ^13^C-NMR (DMSO-*d*_6_) δ 52.3, 55.1, 55.7, 111.3, 114.3, 120.3, 121.7, 122.1, 123.3, 124.9, 126.5, 126.6, 146.1, 149.6, 159.0, 164.5; LC-MS (ESI) *m/z* 339 ([M + 1]^+^).

*2-[4-(4-Cyanophenyl)-1H-1,2,3-triazol-1-yl]-N-(2-methoxyphenyl)acetamide* (**1dac**). White solid; Yield: 86%. ^1^H-NMR (DMSO-*d*_6_) δ 3.88 (s, 3H), 5.52 (s, 2H), 6.91 (m, 1H), 7.08–7.13 (m, 2H), 7.92–7.94 (m, 3H), 8.08 (d, *J* = 8.4 Hz, 2H), 8.80 (s, 1H), 9.81 (s, 1H); ^13^C-NMR (DMSO-*d_6_*) δ 52.4, 55.7, 110.1, 111.3, 120.3, 121.8, 124.8, 125.0, 125.7, 126.5, 132.6, 132.6, 133.0, 135.2, 144.6, 149.6, 164.3; LC-MS (ESI) *m/z* 334 ([M + 1]^+^).

*N-(2-Methoxyphenyl)-2-[4-(thiophen-3-yl)-1H-1,2,3-triazol-1-yl]acetamide* (**1dad**). Light brown solid; Yield: 91%. ^1^H-NMR (CDCl_3_) δ 3.81 (s, 3H), 5.25 (s, 2H), 6.85 (dd, *J* = 1.0, 8.2 Hz, 1H), 6.96 (dt, *J* = 1.1, 7.9 Hz, 1H), 7.08 (dt, *J* = 1.4, 7.9 Hz, 1H), 7.41 (dd, *J* = 3.0, 5.0 Hz, 1H), 7.49 (dd, *J* = 1.2, 5.1 Hz, 1H), 7.74 (dd, *J* = 1.2, 3.0 Hz, 1H), 7.89 (s, 1H), 8.22 (br s, 1H), 8.27 (dd, *J* = 1.5, 8.1 Hz, 1H); ^13^C-NMR (DMSO-*d*_6_) δ 52.3, 55.7, 111.3, 120.3, 120.8, 121.7, 122.8, 124.9, 125.8, 126.6, 127.1, 132.0, 142.7, 149.6, 164.4; LC-MS (ESI) *m/z* 315 ([M + 1]^+^).

*N-(2-Methoxyphenyl)-2-[4-(pyridin-2-yl)-1H-1,2,3-triazol-1-yl]acetamide* (**1dae**). Light brown solid; Yield: 84%. ^1^H-NMR (CDCl_3_) δ 3.80 (s, 3H), 5.28 (s, 2H), 6.85 (d, *J* = 8.2 Hz, 1H), 6.95 (m, 1H), 7.07 (dt, *J* = 1.4, 7.9 Hz, 1H), 7.27 (m, 1H), 7.80 (dt, *J* = 1.7, 7.8 Hz, 1H), 8.20 (d, *J* = 7.9 Hz, 1H), 8.25 (br s, 1H), 8.27 (dd, *J* = 1.4, 8.1 Hz, 1H), 8.34 (s, 1H), 8.61 (d, *J* = 4.2 Hz, 1H); ^13^C-NMR (DMSO-*d*_6_) δ 52.4, 55.8, 111.4, 119.5, 120.4, 121.9, 123.1, 125.06., 125.10, 126.6, 137.3, 147.1, 149.7, 150.1, 164.5; LC-MS (ESI) *m/z* 310 ([M + 1]^+^).

*N-(2-Methoxyphenyl)-2-[4-(3-methoxyphenyl)-1H-1,2,3-triazol-1-yl]acetamide* (**1dag**). White solid; Yield: 86%. ^1^H-NMR (CDCl_3_) δ 3.81 (s, 3H), 3.88 (s, 3H), 5.26 (s, 2H), 6.85 (d, *J* = 8.2 Hz, 1H), 6.92 (ddd, *J* = 1.0, 2.6, 8.1 Hz, 1H), 6.96 (dt, *J* = 1.0, 7.8 Hz, 1H), 7.08 (dt, *J* = 1.4, 7.9 Hz, 1H), 7.35 (t, *J* = 7.9 Hz, 1H), 7.40 (td, *J* = 1.2, 7.6 Hz, 1H), 7.48 (dd, *J* = 1.5, 2.4 Hz, 1H), 7.98 (s, 1H), 8.23 (br s, 1H), 8.27 (dd, *J* = 1.3, 8.1 Hz, 1H); ^13^C-NMR (DMSO-*d*_6_) δ 52.3, 55.1, 55.7, 110.3, 111.3, 113.6, 117.5, 120.3, 121.8, 123.3, 124.9, 126.6, 130.1, 132.1, 146.1, 149.6, 159.7, 164.4; LC-MS (ESI) *m/z* 339 ([M + 1]^+^).

*N-(2-Methoxyphenyl)-2-[4-(2-methoxyphenyl)-1H-1,2,3-triazol-1-yl]acetamide* (**1dah**). Light yellow solid; Yield: 80%. ^1^H-NMR (CDCl_3_) δ 3.77 (s, 3H), 3.95 (s, 3H), 5.26 (s, 2H), 6.83 (dd, *J* = 1.1, 8.2 Hz, 1H), 6.95 (dt, *J* = 1.2, 7.8 Hz, 1H), 7.00 (dd, *J* = 0.6, 8.3 Hz, 1H), 7.06 (dt, *J* = 1.5, 7.9 Hz, 1H), 7.11 (dt, *J* = 1.0, 7.6 Hz, 1H), 7.35 (ddd, *J* = 1.7, 7.4, 8.3 Hz, 1H), 8.25 (s, 1H), 8.27 (br s, 1H), 8.28 (dd, *J* = 1.5, 8.1 Hz, 1H), 8.39 (dd, *J* = 1.7, 7.7 Hz, 1H); ^13^C-NMR (DMSO-*d*_6_) δ 52.2, 55.5, 55.7, 111.3, 111.5, 119.1, 120.3, 120.7, 121.7, 124.9, 125.6, 126.5, 126.6, 128.9, 141.6, 149.6, 155.3, 164.6; LC-MS (ESI) *m/z* 339 ([M + 1]^+^).

*N-(2-Methoxyphenyl)-2-(4-p-tolyl-1H-1,2,3-triazol-1-yl)acetamide* (**1dai**). Light yellow solid; Yield: 78%. ^1^H-NMR (CDCl_3_) δ 2.39 (s, 3H), 3.80 (s, 3H), 5.25 (s, 2H), 6.85 (d, *J* = 8.2 Hz, 1H), 6.96 (t, *J* = 7.8 Hz, 1H), 7.07 (dt, *J* = 1.4, 7.9 Hz, 1H), 7.26 (d, *J* = 7.8 Hz, 2H), 7.75 (d, *J* = 8.2 Hz, 2H), 7.94 (s, 1H), 8.23 (br s, 1H), 8.27 (dd, *J* = 1.5, 8.0 Hz, 1H); ^13^C-NMR (DMSO-*d*_6_) δ 20.8, 52.3, 55.7, 111.3, 120.3, 121.7, 122.6, 124.9, 125.1, 126.6, 128.0, 129.5, 137.1, 146.2, 149.6, 164.5; LC-MS (ESI) *m/z* 323 ([M + 1]^+^).

*N-(2-Methoxyphenyl)-2-(4-m-tolyl-1H-1,2,3-triazol-1-yl)acetamide* (**1daj**). Light yellow solid; Yield: 85%. ^1^H-NMR (CDCl_3_) δ 2.42 (s, 3H), 3.80 (s, 3H), 5.26 (s, 2H), 6.85 (dd, *J* = 0.9, 8.2 Hz, 1H), 6.95 (dt, *J* = 1.0, 7.8 Hz, 1H), 7.08 (dt, *J* = 1.4, 7.8 Hz, 1H), 7.18 (d, *J* = 7.6 Hz, 1H), 7.33 (t, *J* = 7.6 Hz, 1H), 7.64 (d, *J* = 7.7 Hz, 1H), 7.72 (s, 1H), 7.97 (s, 1H), 8.25 (br s, 1H), 8.27 (dd, *J* = 1.4, 8.1 Hz, 1H); ^13^C-NMR (DMSO-*d*_6_) δ 21.1, 52.3, 55.7, 111.3, 120.3, 121.7, 122.3, 123.0, 124.9, 125.7, 126.6, 128.5, 128.8, 130.6, 138.1, 146.3, 149.6, 164.4; LC-MS (ESI) *m/z* 323 ([M + 1]^+^).

*2-{4-[4-(Dimethylamino)phenyl]-1H-1,2,3-triazol-1-yl}-N-(2-methoxyphenyl)acetamide* (**1dal**). Brown solid; Yield: 88%. ^1^H-NMR (CDCl_3_) δ 3.00 (s, 6H), 3.78 (s, 3H), 5.23 (s, 2H), 6.78 (d, *J* = 8.9 Hz, 2H), 6.84 (dd, *J* = 1.0, 8.1 Hz, 1H), 6.95 (dt, *J* = 1.1, 7.8 Hz, 1H), 7.07 (dt, *J* = 1.4, 7.8 Hz, 1H), 7.73 (d, *J* = 9.0 Hz, 2H), 7.83 (s, 1H), 8.24 (br s, 1H), 8.27 (dd, *J* = 1.5, 8.1 Hz, 1H); ^13^C-NMR (DMSO-*d*_6_) δ 40.0, 52.3, 55.7, 111.3, 112.4, 118.6, 120.3, 121.1, 121.7, 124.9, 126.0, 146.8, 149.6, 150.0, 164.5; LC-MS (ESI) *m/z* 352 ([M + 1]^+^).

*N-(2-Methoxyphenyl)-2-[4-(4-phenoxyphenyl)-1H-1,2,3-triazol-1-yl]acetamide* (**1dam**). Yellow solid; Yield: 92%. ^1^H-NMR (CDCl_3_) δ 3.81 (s, 3H), 5.26 (s, 2H), 6.86 (dd, *J* = 1.0, 8.2 Hz, 1H), 6.96 (dt, *J* = 1.1, 7.8 Hz, 1H), 7.02–7.09 (m, 5H), 7.14 (m, 1H), 7.35–7.38 (m, 2H), 7.83 (d, *J* = 8.8 Hz, 2H), 7.94 (s, 1H), 8.24 (br s, 1H), 8.27 (dd, *J* = 1.5, 8.1 Hz, 1H); ^13^C-NMR (DMSO-*d*_6_) δ 52.3, 55.7, 111.3, 118.8, 120.3, 121.7, 122.7, 123.6, 124.9, 126.1, 126.6, 126.9, 130.1, 145.7, 149.6, 156.40, 156.41, 166.4; LC-MS (ESI) *m/z* 401 ([M + 1]^+^).

*2-[4-(2,4-Difluorophenyl)-1H-1,2,3-triazol-1-yl]-N-(2-methoxyphenyl)acetamide* (**1dan**). Light yellow solid; Yield: 94%. ^1^H-NMR (CDCl_3_) δ 3.82 (s, 3H), 5.28 (s, 2H), 6.86 (dd, *J* = 1.0, 8.2 Hz, 1H), 6.93 (ddd, *J* = 2.5, 8.6, 11.0 Hz, 1H), 6.96 (dt, *J* = 1.1, 7.8 Hz, 1H), 7.03 (m, 1H), 7.08 (dt, *J* = 1.4, 7.9 Hz, 1H), 8.12 (d, *J* = 3.6 Hz, 1H), 8.21 (br s, 1H), 8.27 (dd, *J* = 1.4, 8.1 Hz, 1H), 8.31 (dt, *J* = 6.5, 8.6 Hz, 1H); ^13^C-NMR (DMSO-*d*_6_) δ 52.3, 55.7, 104.6 (t, *J* = 26.0 Hz), 111.3, 112.3 (dd, *J* = 21.4, 3.5 Hz), 115.2 (dd, *J* = 13.5, 3.7 Hz), 120.3, 121.8, 124.9, 125.2 (d, *J* = 11.1 Hz), 126.6, 128.5 (dd, *J* = 9.7, 5.3 Hz), 138.8 (d, *J* = 2.9 Hz), 149.6, 158.5 (dd, *J*_CF_ = 250.5, 12.5 Hz), 161.7 (dd, *J*_CF_ = 247.4, 12.5 Hz), 164.4; LC-MS (ESI) *m/z* 345 ([M + 1]^+^).

*2-(4-Benzyl-1H-1,2,3-triazol-1-yl)-N-(2-methoxyphenyl)acetamide* (**1dao**). White solid; Yield: 88%. ^1^H-NMR (CDCl_3_) δ 3.77 (s, 3H), 4.14 (s, 2H), 5.14 (s, 2H), 6.84 (dd, *J* = 0.9, 8.2 Hz, 1H), 6.94 (dt, *J* = 1.1, 7.8 Hz, 1H), 7.07 (dt, *J* = 1.4, 7.9 Hz, 1H), 7.20–7.32 (m, 5H), 7.40 (s, 1H), 8.20 (br s, 1H), 8.25 (dd, *J* = 1.4, 8.0 Hz, 1H); ^13^C-NMR (DMSO-*d*_6_) δ 31.2, 52.1, 55.7, 111.3, 120.3, 121.6, 124.1, 124.8, 126.1, 126.5, 128.4, 128.5, 139.6, 145.8, 149.5, 164.5; LC-MS (ESI) *m/z* 323 ([M + 1]^+^).

*N-Butyl-2-(4-phenyl-1H-1,2,3-triazol-1-yl)acetamide* (**1eaa**). White solid; Yield: 81%. ^1^H-NMR (DMSO-*d*_6_) δ 0.88 (t, *J* = 7.3 Hz, 3H), 1.31 (m, 2H), 1.43 (m, 2H), 3.12 (m, 2H), 5.11 (s, 2H), 7.33 (t, *J* = 7.4 Hz, 1H), 7.45 (t, *J* = 7.7 Hz, 2H), 7.86 (d, *J* = 7.5 Hz, 2H), 8.32 (br s, 1H), 8.51 (s, 1H); ^13^C-NMR (DMSO-*d*_6_) δ 13.6, 19.5, 31.0, 38.4, 51.8, 122.9, 125.1, 127.8, 128.9, 130.7, 146.1, 165.1; LC-MS (ESI) *m/z* 259 ([M + 1]^+^).

*N-Butyl-2-[4-(4-methoxyphenyl)-1H-1,2,3-triazol-1-yl]acetamide* (**1eab**). White solid; Yield: 88%. ^1^H-NMR (DMSO-*d*_6_) δ 0.88 (t, *J* = 7.3 Hz, 3H), 1.31 (m, 2H), 1.43 (m, 2H), 3.11 (m, 2H), 3.79 (s, 3H), 5.09 (s, 2H), 7.01 (d, *J* = 8.8 Hz, 2H), 7.78 (d, *J* = 8.8 Hz, 2H), 8.31 (br s, 1H), 8.39 (s, 1H); ^13^C-NMR (DMSO-*d*_6_) δ 13.6, 19.5, 31.0, 38.4, 51.7, 55.1, 114.3, 121.9, 123.4, 126.4, 146.0, 158.9, 165.2; LC-MS (ESI) *m/z* 289 ([M + 1]^+^).

*N-Butyl-2-[4-(4-cyanophenyl)-1H-1,2,3-triazol-1-yl]acetamide* (**1eac**). White solid; Yield: 83%. ^1^H-NMR (DMSO-*d*_6_) δ 0.88 (t, *J* = 7.3 Hz, 3H), 1.31 (m, 2H), 1.43 (m, 2H), 3.09 (m, 2H), 5.15 (s, 2H), 7.92 (d, *J* = 8.5 Hz, 2H), 8.07 (d, *J* = 8.5 Hz, 2H), 8.36 (br s, 1H), 8.74 (s, 1H); ^13^C-NMR (DMSO-*d*_6_) δ 13.6, 19.5, 31.0, 38.5, 51.9, 110.0, 118.8, 124.6, 125.6, 133.0, 135.2, 144.5, 164.9; LC-MS (ESI) *m/z* 284 ([M + 1]^+^).

*N-Butyl-2-[4-(thiophen-3-yl)-1H-1,2,3-triazol-1-yl]acetamide* (**1ead**). White solid; Yield: 80%. ^1^H-NMR (DMSO-*d*_6_) δ 0.88 (t, *J* = 7.3 Hz, 3H), 1.31 (m, 2H), 1.43 (m, 2H), 3.11 (m, 2H), 5.10 (s, 2H), 7.53 (dd, *J* = 1.3, 5.0 Hz, 1H), 7.64 (dd, *J* = 2.9, 4.9 Hz, 1H), 7.86 (dd, *J* = 1.2, 2.9 Hz, 1H), 8.32 (br s, 1H), 8.38 (s, 1H); ^13^C-NMR (DMSO-*d*_6_) δ 13.6, 13.7, 19.5, 21.6, 24.6, 31.0, 31.1, 38.4, 51.5, 123.2, 146.5, 165.3; LC-MS (ESI) *m/z* 265 ([M + 1]^+^).

*N-Butyl-2-[4-(pyridin-2-yl)-1H-1,2,3-triazol-1-yl]acetamide* (**1eae**). Light yellow solid; Yield: 89%. ^1^H-NMR (DMSO-*d*_6_) δ 0.88 (t, *J* = 7.3 Hz, 3H), 1.31 (m, 2H), 1.43 (m, 2H), 3.11 (m, 2H), 5.15 (s, 2H), 7.34 (dd, *J* = 5.1, 6.6 Hz, 1H), 7.89 (dt, *J* = 1.7, 7.7 Hz, 1H), 8.03 (d, *J* = 7.8 Hz, 1H),8.31 (br s, 1H), 8.51 (s, 1H), 8.60 (d, *J* = 4.4 Hz, 1H); ^13^C-NMR (DMSO-*d*_6_) δ 13.6,19.5, 31.0, 38.4, 51.8, 119.3, 122.9, 124.8, 137.1, 146.9, 149.6, 150.0, 165.0; LC-MS (ESI) *m/z* 260 ([M + 1]^+^).

*N-Butyl-2-(4-butyl-1H-1,2,3-triazol-1-yl)acetamide* (**1eaf**). White solid; Yield: 89%. ^1^H-NMR (DMSO-*d*_6_) δ 0.87 (t, *J* = 7.3 Hz, 3H), 0.89 (t, *J* = 7.4 Hz, 3H), 1.31 (m, 4H), 1.40 (m, 2H), 1.57 (m, 2H), 2.61 (t, *J* = 7.6 Hz, 2H), 3.09 (m, 2H), 4.99 (s, 2H), 7.76 (s, 1H), 8.23 (br s, 1H); ^13^C-NMR (DMSO-*d*_6_) δ 13.6, 19.5, 31.0, 38.5, 51.7, 120.7, 122.7, 125.8, 127.1, 132.1, 142.7, 165.1; LC-MS (ESI) *m/z* 239 ([M + 1]^+^).

*N-Isopropyl-2-(4-phenyl-1H-1,2,3-triazol-1-yl)acetamide* (**1faa**). White solid; Yield: 79%. ^1^H-NMR (DMSO-*d*_6_) δ 1.10 (d, *J* = 6.6 Hz, 6H), 3.86 (m, 1H), 5.08 (s, 2H), 7.33 (t, *J* = 7.4 Hz, 1H), 7.45 (t, *J* = 7.7 Hz, 2H), 7.86 (d, *J* = 7.1 Hz, 1H), 8.29 (d, *J* = 7.4 Hz, 1H), 8.51 (s, 1H); ^13^C-NMR (DMSO-*d*_6_) δ 22.3, 40.9, 51.8, 122.9, 125.1, 127.8, 128.9, 130.8, 146.1, 164.2; LC-MS (ESI) *m/z* 245 ([M + 1]^+^).

*N-Isopropyl-2-[4-(4-methoxyphenyl)-1H-1,2,3-triazol-1-yl]acetamide* (**1fab**). Light yellow solid; Yield: 87%. ^1^H-NMR (DMSO-*d*_6_) δ 1.10 (d, *J* = 6.6 Hz, 6H), 3.79 (s, 3H), 3.85 (m, 1H), 5.05 (s, 2H), 7.01 (d, *J* = 8.8 Hz, 2H), 7.78 (d, *J* = 8.8 Hz, 2H), 8.28 (d, *J* = 7.6 Hz, 1H), 8.39 (s, 1H); ^13^C-NMR (DMSO-*d*_6_) δ 22.3, 40.9, 51.8, 55.1, 114.3, 122.0, 123.4, 126.5, 146.0, 159.0, 164.3; LC-MS (ESI) *m/z* 275 ([M + 1]^+^).

*2-[4-(4-Cyanophenyl)-1H-1,2,3-triazol-1-yl]-N-isopropyl-acetamide* (**1fac**). Light brown solid; Yield: 75%. ^1^H-NMR (DMSO-*d*_6_) δ 1.10 (d, *J* = 6.6 Hz, 6H), 3.85 (m, 1H), 5.12 (s, 2H), 7.92 (d, *J* = 8.6 Hz, 2H), 8.07 (d, *J* = 8.6 Hz, 2H), 8.32 (d, J = 7.4 Hz, 1H), 8.73 (s, 1H); ^13^C-NMR (DMSO-*d*_6_) δ 22.2, 41.0, 51.9, 110.1, 118.8, 124.7, 125.7, 133.0, 135.2, 144.5, 164.0; LC-MS (ESI) *m/z* 270 ([M + 1]^+^).

*N-Isopropyl-2-[4-(thiophen-3-yl)-1H-1,2,3-triazol-1-yl]acetamide* (**1fad**). White solid; Yield: 77%. ^1^H-NMR (DMSO-*d*_6_) δ 1.10 (d, *J* = 6.6 Hz, 6H), 3.85 (m, 1H), 5.07 (s, 2H), 7.53 (dd, *J* = 1.2, 5.0 Hz, 1H), 7.64 (dd, *J* = 2.9, 5.0 Hz, 1H), 7.86 (dd, *J* = 1.2, 2.9 Hz, 1H), 8.28 (d, *J* = 7.0 Hz, 1H), 8.37 (s, 1H); ^13^C-NMR (DMSO-*d*_6_) δ 22.3, 40.9, 51.7, 120.7, 122.7, 125.8, 127.1, 132.1, 142.7, 164.2; LC-MS (ESI) *m/z* 251 ([M + 1]^+^).

*N-Isopropyl-2-[4-(pyridin-2-yl)-1H-1,2,3-triazol-1-yl]acetamide* (**1fae**). White solid; Yield: 84%. ^1^H-NMR (DMSO-*d*_6_) δ 1.10 (d, *J* = 6.6 Hz, 6H), 3.85 (m, 1H), 5.11 (s, 2H), 7.34 (ddd, *J* = 1.2, 4.8, 7.5 Hz, 1H), 7.89 (dt, *J* = 1.8, 7.7 Hz, 1H), 8.03 (td, *J* = 1.0, 7.9 Hz, 1H),8.28 (d, *J* = 7.5 Hz, 1H), 8.51 (s, 1H), 8.60 (m, 1H); ^13^C-NMR (DMSO-*d*_6_) δ 22.3, 41.0, 51.8, 119.4, 122.9, 124.8, 137.2, 146.9, 149.6, 150.0, 164.2; LC-MS (ESI) *m/z* 246 ([M + 1]^+^).

*2-(4-Butyl-1H-1,2,3-triazol-1-yl)-N-isopropylacetamide* (**1faf**). White solid; Yield: 83%. ^1^H-NMR (DMSO-*d*_6_) δ 0.89 (t, *J* = 7.3 Hz, 3H), 1.08 (d, *J* = 6.6 Hz, 3H), 1.32 (m, 2H), 1.57 (m, 2H), 2.61 (d, *J* = 7.6 Hz, 2H), 3.82 (m, 1H), 4.96 (s, 2H), 7.76 (s, 1H), 8.21 (d, *J* = 7.4 Hz, 1H); ^13^C-NMR (DMSO-*d*_6_) δ 13.7, 21.7, 22.2, 24.6, 31.1, 40.8, 51.5, 123.2, 146.5, 164.4; LC-MS (ESI) *m/z* 251 ([M + 1]^+^).

*N-(2-Methoxyphenyl)-2-(4-phenyl-1H-1,2,3-triazol-1-yl)propanamide* (**1dba**). Yellow solid; Yield: 88%. ^1^H-NMR (CDCl_3_) δ 1.98 (d, *J* = 7.3 Hz, 3H), 3.81 (s, 3H), 5.50 (q, *J* = 7.2 Hz, 1H), 6.84 (dd, *J* = 1.2, 8.2 Hz, 1H), 6.95 (dt, *J* = 1.1, 7.8 Hz, 1H), 7.06 (dt, *J* = 1.5, 7.9 Hz, 1H), 7.36 (m, 1H), 7.43–7.46 (m, 2H), 7.85–7.87 (m, 2H), 8.01 (s, 1H), 8.28 (dd, *J* = 1.5, 8.1 Hz, 1H), 8.32 (s, 1H); ^13^C-NMR (DMSO-*d_6_*) δ 18.0, 55.8, 58.5, 111.3, 120.2, 120.8, 122.1, 125.1, 127.8, 128.9, 146.1, 149.9, 167.6; LC-MS (ESI) *m/z* 323 ([M + 1]^+^).

*N-(2-Methoxyphenyl)-2-[4-(4-methoxyphenyl)-1H-1,2,3-triazol-1-yl]propanamide* (**1dbb**). White solid; Yield: 91%. ^1^H-NMR (CDCl_3_) δ 3.79 (s, 3H), 3.85 (s, 3H), 5.25 (s, 2H), 6.84 (dd, *J* = 1.0, 8.2 Hz, 1H), 6.95 (dt, *J* = 1.1, 7.7 Hz, 1H), 6.98 (d, *J* = 8.9 Hz, 2H), 7.07 (dt, *J* = 1.5, 7.9 Hz, 1H), 7.79 (d, *J* = 8.9 Hz, 2H), 7.90 (s, 1H), 8.24 (s, 1H), 8.27 (dd, *J* = 1.5, 8.1 Hz, 1H); ^13^C-NMR (DMSO-*d*_6_) δ 18.0, 55.1, 55.8, 58.5, 111.3, 114.3, 119.9, 120.2, 122.1, 123.4, 125.2, 126.38, 126.44, 146.0, 149.9, 158.9, 167.6; LC-MS (ESI) *m/z* 353 ([M + 1]^+^).

*2-[4-(4-Cyanophenyl)-1H-1,2,3-triazol-1-yl]-N-(2-methoxyphenyl)propanamide* (**1dbc**). Light yellow solid; Yield: 87%. ^1^H-NMR (CDCl_3_) δ 1.98 (d, *J* = 7.2 Hz, 3H),5.53 (q, *J* = 7.2 Hz, 1H), 6.87 (d, *J* = 8.1 Hz, 1H), 6.96 (t, *J* = 7.8 Hz, 1H), 7.08 (dt, *J* = 1.4, 7.9 Hz, 1H), 7.73 (d, *J* = 8.5 Hz, 2H), 7.98 (d, *J* = 8.5 Hz, 2H), 8.15 (s, 1H), 8.27 (dd, *J* = 1.4, 8.1 Hz, 1H), 8.30 (s, 1H); ^13^C-NMR (DMSO-*d*_6_) δ 18.0, 55.8, 58.7, 110.1, 111.4, 118.8, 120.2, 122.2, 122.7, 125.3, 125.6, 126.3, 133.0, 135.2, 144.6, 150.0, 167.5; LC-MS (ESI) *m/z* 348 ([M + 1]^+^).

*N-(2-Methoxyphenyl)-2-[4-(thiophen-3-yl)-1H-1,2,3-triazol-1-yl]propanamide* (**1dbd**). Light brown oil; Yield: 80%. ^1^H-NMR (CDCl_3_) δ 1.94 (d, *J* = 7.3 Hz, 3H), 3.78 (s, 3H), 5.52 (q, *J* = 7.2 Hz, 2H), 6.83 (dd, *J* = 1.1, 8.2 Hz, 1H), 6.90 (dt, *J* = 1.2, 8.4 Hz, 1H), 7.05 (m, 1H), 7.38 (dd, *J* = 3.0, 5.0 Hz, 1H), 7.47 (dd, *J* = 1.2, 5.0 Hz, 1H), 7.71 (dd, *J* = 1.2, 3.0 Hz, 1H), 7.95 (s, 1H), 8.26 (dd, *J* = 1.5, 8.1 Hz, 1H), 8.38 (br s, 1H); ^13^C-NMR (DMSO-*d*_6_) δ 18.1, 55.8, 58.4, 111.4, 120.3, 120.6, 120.7, 122.1, 125.2, 125.8, 127.1, 132.1, 142.7, 149.9, 167.6; LC-MS (ESI) *m/z* 329 ([M + 1]^+^).

*N-(2-Methoxyphenyl)-2-[4-(pyridin-2-yl)-1H-1,2,3-triazol-1-yl]propanamide* (**1dbe**). Colorless oil; Yield: 83%. ^1^H-NMR (CDCl_3_) δ 1.97 (d, *J* = 7.2 Hz, 3H), 3.78 (s, 3H), 5.53 (q, *J* = 7.2 Hz, 1H), 6.82 (dd, *J* = 1.1, 8.2 Hz, 1H), 6.92 (dt, *J* = 1.1, 7.8 Hz, 1H), 7.04 (dt, *J* = 1.5, 7.9 Hz, 1H), 7.24 (ddd, *J* = 1.2, 4.9, 7.6 Hz, 1H), 7.78 (dt, *J* = 1.8, 7.8 Hz, 1H), 8.18 (dd, *J* = 0.9, 8.8 Hz, 1H), 8.26 (dd, *J* = 1.6, 8.1 Hz, 1H), 8.36 (br s, 1H), 8.39 (s, 1H), 8.59 (ddd, *J* = 0.9, 1.7, 4.8 Hz, 1H); ^13^C-NMR (DMSO-*d*_6_) δ 18.0, 55.8, 58.7, 111.4, 119.4, 120.3, 122.2, 122.7, 123.0, 125.2, 126.4, 137.2, 147.0, 149.6, 149.9, 150.0, 167.5; LC-MS (ESI) *m/z* 324 ([M + 1]^+^).

*N-(2-Methoxyphenyl)-2-[4-(3-methoxyphenyl)-1H-1,2,3-triazol-1-yl]propanamide* (**1dbg**). Light yellow oil; Yield: 94%. ^1^H-NMR (CDCl_3_) δ 1.95 (d, *J* = 7.2 Hz, 3H), 3.77 (s, 3H), 3.85 (s, 3H), 5.53 (q, *J* = 7.2 Hz, 1H), 6.82 (dd, J = 1.2, 8.2 Hz, 1H), 6.89 (ddd, J = 1.0, 2.6, 8.2 Hz, 1H), 6.92 (dt, *J* = 1.2, .8 Hz, 1H), 7.04 (dt, *J* = 1.6, 7.9 Hz, 1H), 7.32 (t, *J* = 7.9 Hz, 1H), 7.39 (td, *J* = 1.3, 7.7 Hz, 1H), 7.46 (dd, *J* = 1.5, 2.5 Hz, 1H), 8.04 (s, 1H), 8.26 (dd, *J* = 1.6, 8.1 Hz, 1H), 8.39 (br s, 1H); ^13^C-NMR (DMSO-*d*_6_) δ 18.0, 55.1, 55.8, 58.5, 110.3, 111.4, 113.7, 117.4, 120.3, 121.2, 122.1, 125.2, 126.4, 130.0, 132.1, 146.0, 149.9, 159.7, 167.6; LC-MS (ESI) *m/z* 353 ([M + 1]^+^).

*N-(2-Methoxyphenyl)-2-[4-(2-methoxyphenyl)-1H-1,2,3-triazol-1-yl]propanamide* (**1dbh**). Light yellow oil; Yield: 89%. ^1^H-NMR (CDCl_3_) δ 1.98 (d, *J* = 7.3 Hz, 3H), 3.75 (s, 3H), 3.92 (s, 3H), 5.51 (q, *J* = 7.3 Hz, 1H), 6.80 (dd, *J* = 1.2, 8.2 Hz, 1H), 6.92 (dt, *J* = 1.2, 7.8 Hz, 1H), 6.97 (dd, *J* = 0.6, 8.3 Hz, 1H), 7.03 (dt, *J* = 1.5, 7.9 Hz, 1H), 7.08 (dt, *J* = 1.0, 7.5 Hz, 1H), 7.32 (ddd, *J* = 1.7, 7.4, 8.3 Hz, 1H), 8.25 (s, 1H), 8.27 (dd, *J* = 1.6, 8.1 Hz, 1H), 8.37 (dd, *J* = 1.7, 7.7 Hz, 1H), 8.41 (br s, 1H); ^13^C-NMR (DMSO-*d*_6_) δ 18.1. 55.5. 55.8. 58.5. 111.4. 111.5. 119.0. 120.3. 120.6. 122.1. 123.0. 125.2. 126.4. 126.6. 128.9. 141.7. 149.9. 155.4. 167.6; LC-MS (ESI) *m/z* 353 ([M + 1]^+^).

*N-(2-Methoxyphenyl)-2-(4-p-tolyl-1H-1,2,3-triazol-1-yl)propanamide* (**1dbi**). Light yellow solid; Yield: 94%. ^1^H-NMR (CDCl_3_) δ 1.97 (d, *J* = 7.3 Hz, 3H), 2.38 (s, 3H), 3.80 (s, 3H), 5.49 (q, *J* = 7.2 Hz, 1H), 6.84 (dd, *J* = 1.0, 8.2 Hz, 1H), 6.94 (dt, *J* = 1.1, 7.8 Hz, 1H), 7.06 (dt, *J* = 1.5, 7.9 Hz, 1H), 7.25 (d, *J* = 7.9 Hz, 2H), 7.74 (d, *J* = 8.2 Hz, 2H), 7.97 (s, 1H), 8.28 (dd, *J* = 1.5, 8.1 Hz, 1H), 8.33 (br s, 1H); ^13^C-NMR (DMSO-*d*_6_) δ 18.0, 20.8, 55.8, 58.5, 111.3, 120.2, 120.4, 122.1, 125.0, 125.2, 126.4, 128.0, 129.4, 137.1, 146.2, 149.9, 167.6; LC-MS (ESI) *m/z* 337 ([M + 1]^+^).

*N-(2-Methoxyphenyl)-2-(4-m-tolyl-1H-1,2,3-triazol-1-yl)propanamide* (**1dbj**). Light yellow solid; Yield: 83%. ^1^H-NMR (CDCl_3_) δ 1.97 (d, *J* = 7.3 Hz, 3H), 2.41 (s, 3H), 3.80 (s, 3H), 5.51 (q, *J* = 7.2 Hz, 1H), 6.84 (dd, *J* = 1.1, 8.2 Hz, 1H), 6.94 (dt, *J* = 1.2, 7.8 Hz, 1H), 7.06 (dt, *J* = 1.5, 7.9 Hz, 1H), 7.17 (d, *J* = 7.6 Hz, 1H), 7.32 (t, *J* = 7.7 Hz, 1H), 7.63 (d, *J* = 7.7 Hz, 1H), 7.71 (s, 1H), 8.00 (s, 1H), 8.28 (dd, *J* = 1.5, 8.1 Hz, 1H), 8.34 (br s, 1H); ^13^C-NMR (DMSO-*d_6_*) δ 18.0, 20.8, 55.8, 58.5, 111.3, 120.2, 120.4, 122.1, 125.0, 125.1, 126.4, 128.0, 129.4, 137.1, 146.2, 149.9, 167.6; LC-MS (ESI) *m/z* 337 ([M + 1]^+^).

*N-(2-Methoxyphenyl)-2-(4-o-tolyl-1H-1,2,3-triazol-1-yl)propanamide* (**1dbk**). Light yellow solid; Yield: 74%. ^1^H-NMR (acetone-*d*_6_) δ 1.97 (d, *J* = 7.2 Hz, 3H), 2.52 (s, 3H), 3.84 (s, 3H), 5.85 (q, *J* = 7.3 Hz, 1H), 6.93 (m, 1H), 7.02 (d, *J* = 7.1 Hz, 1H), 7.25–7.32 (m, 3H), 7.82 (m, 1H), 8.26 (m, 1H), 8.41 (s, 1H); ^13^C-NMR (DMSO-*d*_6_) δ 18.5, 21.5, 56.3, 59.1, 111.8, 120.8, 122.6, 123.2, 125.7, 126.5, 127.0, 128.3, 128.8, 130.5, 131.3, 135.5, 146.0, 150.4, 166.3; LC-MS (ESI) *m/z* 337 ([M + 1]^+^).

*2-{4-[4-(Dimethylamino)phenyl]-1H-1,2,3-triazol-1-yl}-N-(2-methoxyphenyl)propanamide* (**1dbl**). Brown solid; Yield: 82%. ^1^H-NMR (CDCl_3_) δ 1.96 (d, *J* = 7.3 Hz, 3H), 3.00 (s, 6H), 3.79 (s, 3H), 5.47 (q, *J* = 7.2 Hz, 2H), 6.77 (d, *J* = 9.0 Hz, 2H), 6.83 (dd, *J* = 1.1, 8.2 Hz, 1H), 6.94 (dt, *J* = 1.2, 7.8 Hz, 1H), 7.05 (dt, *J* = 1.5, 7.9 Hz, 1H), 7.72 (d, *J* = 8.9 Hz, 2H), 7.85 (s, 1H), 8.28 (dd, *J* = 1.6, 8.1 Hz, 1H), 8.32 (br s, 1H); ^13^C-NMR (DMSO-*d*_6_) δ 18.0, 40.0, 55.8, 58.4, 111.3, 112.3, 118.7, 118.9, 120.3, 122.0, 125.1, 126.0, 126.4, 146.7, 149.9, 150.0, 167.7; LC-MS (ESI) *m/z* 366 ([M + 1]^+^).

*N-(2-Methoxyphenyl)-2-[4-(4-phenoxyphenyl)-1H-1,2,3-triazol-1-yl]propanamide* (**1dbm**). Light yellow oil; Yield: 87%. ^1^H-NMR (CDCl_3_) δ 1.97 (d, *J* = 7.3 Hz, 3H), 3.80 (s, 3H), 5.52 (q, *J* = 7.2 Hz, 1H), 6.84 (dd, *J* = 1.2, 8.2 Hz, 1H), 6.94 (m, 1H), 7.02–7.08 (m, 5H), 7.15 (m, 1H), 7.34–7.37 (m, 2H), 7.82 (d, *J* = 8.8 Hz, 2H), 7.99 (s, 1H), 8.28 (dd, *J* = 1.5, 8.1 Hz, 1H), 8.37 (br s, 1H); ^13^C-NMR (DMSO-*d*_6_) δ 18.0, 55.8, 80.0, 111.3, 118.2, 118.81, 118.86, 119.4, 120.3, 120.5, 123.6, 126.9, 130.1, 130.2, 133.6, 145.7, 149.9, 156.39, 156.42, 167.6; LC-MS (ESI) *m/z* 415 ([M + 1]^+^).

*2-[4-(2,4-Difluorophenyl)-1H-1,2,3-triazol-1-yl]-N-(2-methoxyphenyl)propanamide* (**1dbn**). Light yellow solid; Yield: 92%. ^1^H-NMR (CDCl_3_) δ 1.99 (d, *J* = 7.3 Hz, 3H), 3.82 (s, 3H), 5.51 (q, *J* = 7.2 Hz, 2H), 6.85 (dd, *J* = 1.2, 8.2 Hz, 1H), 6.92 (ddd, *J* = 2.4, 8.7, 11.0 Hz, 1H), 6.95 (dt, *J* = 1.1, 7.8 Hz, 1H), 7.01 (m, 1H), 7.07 (dt, *J* = 1.5, 7.9 Hz, 1H), 8.12 (d, *J* = 3.5 Hz, 1H), 8.28 (dd, *J* = 1.6, 8.1 Hz, 1H), 8.29 (dt, *J* = 6.5, 8.6 Hz, 1H), 8.31 (br s, 1H); ^13^C-NMR (DMSO-*d*_6_) δ 18.0, 55.8, 58.7, 104.6 (t, *J*_CF_ = 25.9 Hz), 111.4, 112.30 (dd, *J*_CF_ = 21.6, 3.6 Hz), 115.20 (dd, *J*_CF_ = 13.4, 3.8 Hz), 120.3, 122.2, 122.8 (d, *J*_CF_ = 10.3 Hz), 125.3, 126.3, 128.7 (dd, *J*_CF_ = 9.8, 5.2 Hz), 139.1 (d, *J*_CF_ = 2.6 Hz), 150.0, 158.5 (dd, *J*_CF_ = 250.5, 12.8 Hz), 161.86 (dd, *J*_CF_ = 247.8, 12.6 Hz), 167.5; LC-MS (ESI) *m/z* 359 ([M + 1]^+^).

*2-(4-Benzyl-1H-1,2,3-triazol-1-yl)-N-(2-methoxyphenyl)propanamide* (**1dbo**). Light yellow oil; Yield: 86%. ^1^H-NMR (CDCl_3_) δ 1.88 (d, *J* = 7.3 Hz, 3H), 3.77 (s, 3H), 4.12 (s, 2H), 5.39 (q, *J* = 7.3 Hz, 1H), 6.83 (dd, *J* = 1.1, 8.2 Hz, 1H), 6.93 (dt, *J* = 1.2, 7.8 Hz, 1H), 7.05 (dt, *J* = 1.5, 7.9 Hz, 1H), 7.23 (m, 1H), 7.26–7.31 (m, 4H), 7.42 (s, 1H), 8.25 (dd, *J* = 1.5, 8.1 Hz, 1H), 8.33 (br s, 1H); ^13^C-NMR (DMSO-*d*_6_) δ 18.0, 31.2, 55.7, 58.3, 111.3, 120.2, 121.8, 122.0, 125.1, 126.1, 126.4, 128.4, 128.5, 139.6, 145.8, 149.9, 167.6; LC-MS (ESI) *m/z* 337 ([M + 1]^+^).

*N-Butyl-2-(4-phenyl-1H-1,2,3-triazol-1-yl)propanamide* (**1eba**). White solid; Yield: 83%. ^1^H-NMR (DMSO-*d*_6_) δ 0.86 (t, *J* = 7.3 Hz, 3H), 1.27 (m, 2H), 1.41 (m, 2H), 1.71 (d, *J* = 7.2 Hz, 3H), 3.10 (m, 2H), 5.38 (q, *J* = 7.2 Hz, 1H),, 7.33(t, *J* = 7.4 Hz, 1H), 7.31 (t, *J* = 7.7 Hz, 2H), 7.88 (d, *J* = 7.1 Hz, 2H), 8.38 (br s, 1H), 8.68 (s, 1H); ^13^C-NMR (DMSO-*d*_6_) δ 13.6, 18.0, 19.4, 30.9, 38.4, 58.4, 120.6, 125.1, 127.8, 128.9, 130.9, 146.0, 168.3; LC-MS (ESI) *m/z* 273 ([M + 1]^+^).

*N-Butyl-2-[4-(4-methoxyphenyl)-1H-1,2,3-triazol-1-yl]propanamide* (**1ebb**). White solid; Yield: 81%. ^1^H-NMR (DMSO-*d*_6_) δ 0.86 (t, *J* = 7.3 Hz, 3H), 1.27 (m, 2H), 1.40 (m, 2H), 1.70 (d, *J* = 7.2 Hz, 3H), 3.79 (s, 3H), 5.36 (q, *J* = 7.2 Hz, 1H), 7.01(d, *J* = 8.8 Hz, 2H), 7.80 (d, *J* = 8.8 Hz, 2H), 8.36 (br s, 1H), 8.56 (s, 1H); ^13^C-NMR (DMSO-*d*_6_) δ 13.6, 18.0, 19.4, 30.9, 38.4, 55.1, 58.3, 114.3, 119.6, 123.5, 126.4, 146.0, 158.9, 168.4; LC-MS (ESI) *m/z* 303 ([M + 1]^+^).

*N-Butyl-2-[4-(4-cyanophenyl)-1H-1,2,3-triazol-1-yl]propanamide* (**1ebc**). White solid; Yield: 79%. ^1^H-NMR (DMSO-*d*_6_) δ 0.86 (t, *J* = 7.3 Hz, 3H), 1.27 (m, 2H), 1.40 (m, 2H), 1.72 (d, *J* = 7.2 Hz, 3H), 3.09 (m, 2H), 5.42 (q, *J* = 7.2 Hz, 1H), 7.92 (d, *J* = 8.6 Hz, 2H), 8.09 (d, *J* = 8.6 Hz, 2H), 8.40 (br s, 1H), 8.92 (s, 1H); ^13^C-NMR (DMSO-*d*_6_) δ 13.6, 18.0, 19.4, 30.9, 38.5, 58.5, 110.0, 118.8, 122.5, 125.6, 133.0, 135.3, 144.5, 168.2; LC-MS (ESI) *m/z* 298 ([M + 1]^+^).

*N-Butyl-2-[4-(thiophen-3-yl)-1H-1,2,3-triazol-1-yl]propanamide* (**1ebd**). Light yellow solid; Yield: 78%. ^1^H-NMR (DMSO-*d*_6_) δ 0.86 (t, *J* = 7.3 Hz, 3H), 1.27 (m, 2H), 1.40 (m, 2H), 1.69 (d, *J* = 7.2 Hz, 3H), 3.08 (m, 2H), 5.37 (q, *J* = 7.1 Hz, 1H), 7.55 (dd, *J* = 1.2, 5.0 Hz, 1H), 7.64 (dd, *J* = 2.9, 5.0 Hz, 1H), 7.86 (dd, *J* = 1.2, 2.9 Hz, 1H), 8.38 (br s, 1H), 8.55 (s, 1H); ^13^C-NMR (DMSO-*d*_6_) δ 13.6, 18.0, 19.4, 30.9, 38.4, 58.3, 120.3, 120.6, 125.8, 127.0, 132.2, 142.7, 168.3; LC-MS (ESI) *m/z* 279 ([M + 1]^+^).

*N-Butyl-2-[4-(pyridin-2-yl)-1H-1,2,3-triazol-1-yl]propanamide* (**1ebe**). White solid; Yield: 84%. ^1^H-NMR (DMSO-*d*_6_) δ 0.86 (t, *J* = 7.3 Hz, 3H), 1.27 (m, 2H), 1.40 (m, 2H), 1.73 (d, *J* = 7.2 Hz, 3H), 3.09 (m, 2H), 5.42 (q, *J* = 7.2 Hz, 1H), 7.35 (m, 1H), 7.89 (dt, *J* = 1.7, 7.7 Hz, 1H), 8.03 (d, *J* = 7.9 Hz, 1H), 8.37 (br s, 1H), 8.61 (m, 1H), 8.62 (s, 1H); ^13^C-NMR (DMSO-*d*_6_) δ 13.6, 18.0, 19.4, 30.9, 38.5, 58.5, 119.4, 122.4, 123.0, 137.2, 147.0, 149.6, 150.0, 168.2; LC-MS (ESI) *m/z* 274 ([M + 1]^+^).

*N-Butyl-2-(4-butyl-1H-1,2,3-triazol-1-yl)propanamide* (**1ebf**). White solid; Yield: 81%. ^1^H-NMR (DMSO-*d*_6_) δ 0.85(t, *J* = 7.3 Hz, 3H), 0.89(t, *J* = 7.4 Hz, 3H), 1.26 (m, 2H), 1.32 (m, 2H), 1.37 (m, 2H), 1.59 (m, 2H), 1.61 (d, *J* = 7.2 Hz, 3H), 2.60 (d, *J* = 7.6 Hz, 2H), 3.06 (m, 2H), 5.27 (d, *J* = 7.2 Hz, 1H), 7.88 (s, 1H), 8.29 (br s, 1H); ^13^C-NMR (DMSO-*d*_6_) δ 13.6, 13.7, 18.0, 19.4, 21.7, 24.7, 30.9, 31.1, 38.4, 58.0, 120.7, 146.6, 168.4; LC-MS (ESI) *m/z* 253 ([M + 1]^+^).

*N-Isopropyl-2-(4-phenyl-1H-1,2,3-triazol-1-yl)propanamide* (**1fba**). White solid; Yield: 88%. ^1^H-NMR (DMSO-*d*_6_) δ 1.07 (d, *J* = 6.6 Hz, 3H), 1.09 (d, *J* = 6.6 Hz, 3H), 1.70 (d, *J* = 7.2 Hz, 3H), 3.81 (m, 1H), 5.35 (q, *J* = 7.1 Hz, 1H), 7.33 (m, 1H), 7.45 (t, *J* = 7.7 Hz, 2H), 7.88 (d, *J* = 7.1 Hz, 1H), 8.33 (d, *J* = 7.5 Hz, 1H), 8.68 (s, 1H); ^13^C-NMR (DMSO-*d*_6_) δ 18.0, 22.1, 40.9, 58.3, 120.6, 125.1, 127.8, 128.9, 130.9, 146.0, 167.5; LC-MS (ESI) *m/z* 259 ([M + 1]^+^).

*N-Isopropyl-2-[4-(4-methoxyphenyl)-1H-1,2,3-triazol-1-yl]propanamide* (**1fbb**). Light brown solid; Yield: 83%. ^1^H-NMR (DMSO-*d*_6_) δ 1.06 (d, *J* = 6.6 Hz, 3H), 1.09 (d, *J* = 6.6 Hz, 3H), 1.69 (d, *J* = 7.2 Hz, 3H), 3.79 (s, 3H), 3.82 (m, 1H), 5.33 (q, *J* = 7.2 Hz, 1H), 7.01 (d, *J* = 8.8 Hz, 2H), 7.80 (d, *J* = 8.8 Hz, 2H), 8.32 (d, *J* = 7.5 Hz, 1H), 8.56 (s, 1H); ^13^C-NMR (DMSO-*d*_6_) δ 18.0, 22.1, 40.9, 55.1, 58.3, 114.3, 119.6, 123.5, 126.4, 145.9, 158.9, 167.5; LC-MS (ESI) *m/z* 289 ([M + 1]^+^).

*2-[4-(4-Cyanophenyl)-1H-1,2,3-triazol-1-yl]-N-isopropyl-propanamide* (**1fbc**). White solid; Yield: 78%. ^1^H-NMR (DMSO-*d*_6_) δ 1.07 (d, *J* = 6.6 Hz, 3H), 1.09 (d, *J* = 6.6 Hz, 3H), 1.71 (d, *J* = 7.3 Hz, 3H), 3.80 (m, 1H), 5.38 (q, *J* = 7.2 Hz, 1H), 7.92 (d, *J* = 8.4 Hz, 2H), 8.09 (d, *J* = 8.4 Hz, 2H), 8.36 (d, *J* = 7.3 Hz, 1H), 8.93 (s, 1H); ^13^C-NMR (DMSO-*d*_6_) δ 18.0, 22.1, 40.9, 58.4, 110.0, 118.8, 122.5, 125.6, 133.0, 135.3, 144.5, 167.3; LC-MS (ESI) *m/z* 284 ([M + 1]^+^).

*N-Isopropyl-2-[4-(thiophen-3-yl)-1H-1,2,3-triazol-1-yl]propanamide* (**1fbd**). White solid; Yield: 75%. ^1^H-NMR (DMSO-*d*_6_) δ 1.06 (d, *J* = 6.6 Hz, 3H), 1.09 (d, *J* = 6.6 Hz, 3H), 1.68 (d, *J* = 7.2 Hz, 3H), 3.81 (m, 1H), 5.34 (q, *J* = 7.1 Hz, 1H), 7.55 (dd, *J* = 1.2, 5.0 Hz, 1H), 7.64 (dd, *J* = 2.9, 5.0 Hz, 1H), 7.86 (dd, *J* = 1.2, 3.0 Hz, 1H), 8.33 (d, *J* = 7.4 Hz, 1H), 8.55 (s, 1H); ^13^C-NMR (DMSO-*d*_6_) δ 18.1, 22.1, 40.9, 58.2, 120.3, 120.6, 125.8, 127.0, 132.2, 142.6, 167.5; LC-MS (ESI) *m/z* 265 ([M + 1]^+^).

*N-Isopropyl-2-[4-(pyridin-2-yl)-1H-1,2,3-triazol-1-yl]propanamide* (**1fbe**). White solid; Yield: 81%. ^1^H-NMR (DMSO-*d*_6_) δ 1.06 (d, *J* = 6.6 Hz, 3H), 1.09 (d, *J* = 6.6 Hz, 3H), 1.72 (d, *J* = 7.2 Hz, 3H), 3.82 (m, 1H), 5.38 (q, *J* = 7.2 Hz, 1H), 7.35 (ddd, *J* = 1.2, 4.8, 7.5 Hz, 1H), 7.89 (dt, *J* = 1.8, 7.7 Hz, 1H), 8.31 (d, *J* = 7.5 Hz, 1H), 8.60 (m, 1H), 8.61 (s, 1H); ^13^C-NMR (DMSO-*d*_6_) δ 18.0, 22.1, 40.9, 58.4, 119.4, 122.3, 122.9, 137.2, 146.9, 149.6, 150.0, 167.3; LC-MS (ESI) *m/z* 260 ([M + 1]^+^).

*2-(4-Butyl-1H-1,2,3-triazol-1-yl)- N-isopropyl-propanamide* (**1fbf**). White solid; Yield: 82%. ^1^H-NMR (DMSO-*d*_6_) δ 0.89 (t, *J* = 7.43 Hz, 3H), 1.04 (d, *J* = 6.6 Hz, 3H), 1.07 (d, *J* = 6.6 Hz, 3H), 1.32 (m, 2H), 1.57 (m, 2H), 1.61 (d, *J* = 7.2 Hz, 3H), 2.60 (t, *J* = 7.6 Hz, 2H), 3.79 (m, 1H), 5.24 (q, *J* = 7.2 Hz, 1H), 7.88 (s, 1H), 8.24 (d, *J* = 7.4 Hz, 1H); ^13^C-NMR (DMSO-*d*_6_) δ 13.7, 18.1, 21.7, 22.1, 24.7, 31.1, 40.8, 58.0, 120.7, 146.5, 167.6; LC-MS (ESI) *m/z* 239 ([M + 1]^+^).

### 3.4. General Procedure for the Preparation of Tertiary 1,2,3-Triazoloamides ***2*** in Solution-Phase

A typical procedure for the desired tertiary 1,2,3-triazoloamide **2**, as exemplified for 1-morpholino-2-(4-phenyl-1*H*-1,2,3-triazol-1-yl)ethanone (**2aaa**; R^1^R^2^N = morpholine, **A** = CH_2_, R^3^ = Ph).

#### 3.4.1. Preparation of Chloro-Amide **15**

To a solution of morpholine (**14a**; 4.50 mL, 51.45 mmol) and triethylamine (7.90 mL, 56.68 mmol) in CH_2_Cl_2_ (80 mL) was slowly added 2-chloroacetyl chloride (**6a**; 3.70 mL, 46.45 mmol) at 0 °C. The reaction mixture was stirred at room temperature for 6 h, and then diluted with CH_2_Cl_2_, washed with saturated NaHCO_3_ and brine, dried over MgSO_4_ and filtered. The residue was concentrated under reduced pressure to afford α-chloroamide **15aa** (7.55 g, 99%) as a light yellow liquid: ^1^H-NMR (CDCl_3_) δ 3.54 (t, *J* = 4.5 Hz, 2H), 3.64 (t, *J* = 4.4 Hz, 2H), 3.70–3.74 (m, 4H), 4.07 (s, 2H); ^13^C-NMR (CDCl_3_) δ 40.7, 42.5, 46.8, 66.6, 66.7, 165.3; LC-MS (ESI) *m*/*z* 164 ([M + 1]^+^).

#### 3.4.2. Preparation of Azidoamide **16**

To a solution of α-chloroamide **15aa** (1.41 g, 8.62 mmol) in acetonitrile (20 mL) and H_2_O (1 mL) was added sodium azide (700 mg, 10.77 mmol). The reaction mixture was stirred at room temperature for 1 day, and then diluted with EtOAc, washed with brine, dried over MgSO_4_ and filtered. The solvent was removed, and the residue was passed through a short plug of silica to give α-azidoamide **16aa** (1.40 g, 99%) as a colorless oil: ^1^H-NMR (CDCl_3_) δ 3.38 (t, *J* = 4.8 Hz, 2H), 3.64 (t, *J* = 4.4 Hz, 2H), 3.67–3.71 (m, 4H), 3.92 (s, 2H); ^13^C-NMR (CDCl_3_) δ 42.3, 45.6, 50.6, 66.5, 66.8, 165.9; LC-MS (ESI) *m*/*z* 171 ([M + 1]^+^).

#### 3.4.3. Preparation of Tertiary 1,2,3-Triazoloamide **2**

To a mixture of α-azidoamide **16aa** (34 mg, 0.20 mmol) and phenylacetylene (**9a**; 24 μL, 0.20 mmol) in *t-*BuOH/H_2_O (2 mL, 1:1) were added 0.5 M CuSO_4_ (0.020 mL, 0.010 mmol) and 1.0 M sodium ascorbate (0.020 mL, 0.020 mmol). The reaction mixture was stirred at room temperature for 1 day, and then the resulting reaction mixture was filtered. The separated solid was washed with H_2_O and hexanes, and triturated with hexane/EtOAc (10:1) to give the 1-morpholino-2-(4-phenyl-1*H*-1,2,3-triazol-1-yl)ethanone (**2aaa**; 52 mg, 96%) as a white solid: Mp 229–231 °C; ^1^H-NMR (CDCl_3_) δ 3.62 (t, *J* = 4.7 Hz, 2H), 3.66 (t, *J* = 4.6 Hz, 2H), 3.71 (m, 4H), 5.28 (s, 2H), 7.34 (m, 1H), 7.41–7.45 (m, 2H), 7.84–7.86 (m, 2H), 7.99 (s, 1H); ^13^C-NMR (CDCl_3_) δ 42.7, 46.0, 51.0, 66.5, 66.7, 121.3, 125.9, 128.4, 129.0, 130.5, 148.3, 163.7; IR (ATR) υ_max_ 3134, 2984, 2858, 1661, 1643, 1471, 1426, 1236, 1112, 1036, 768 (cm^−1^); LC-MS (ESI) *m/z* 273 ([M + 1]^+^); HRMS (FAB) calcd for C_14_H_17_N_4_O_2_ ([M + H]^+^) 273.1346, found 273.1342.

### 3.5. Characterization Data of Tertiary 1,2,3-Triazoloamides ***2***

*2-[4-(4-Methoxyphenyl)-1H-1,2,3-triazol-1-yl]-1-morpholinoethanone* (**2aab**). White solid; Yield: 96%. ^1^H-NMR (CDCl_3_) δ 3.62 (t, *J* = 4.7 Hz, 2H), 3.66 (t, *J* = 4.6 Hz, 2H), 3.70 (m, 4H), 3.85 (s, 3H), 5.26 (s, 2H), 6.97 (d, *J* = 8.7 Hz, 2H), 7.77 (d, *J* = 8.7 Hz, 2H), 7.89 (s, 1H); ^13^C-NMR (DMSO-*d*_6_) δ 41.9, 44.7, 50.9, 65.87, 65.94, 114.3, 122.1, 123.4, 126.4, 146.0, 158.9, 164.5; LC-MS (ESI) *m/z* 303 ([M + 1]^+^).

*4-[1-(2-Morpholino-2-oxoethyl)-1H-1,2,3-triazol-4-yl]benzonitrile* (**2aac**). Yellow solid; Yield: 93%. ^1^H-NMR (CDCl_3_) δ 3.62 (t, *J* = 4.8 Hz, 2H), 3.67 (t, *J* = 4.8 Hz, 2H), 3.72–3.76 (m, 4H), 5.31 (s, 2H), 7.72 (d, *J* = 8.3 Hz, 2H), 7.96 (d, *J* = 8.3 Hz, 2H), 8.10 (s, 1H); ^13^C-NMR (DMSO-*d*_6_) δ 41.9. 44.7. 50.9. 65.86. 65.92. 110.2. 118.8. 124.8. 125.7. 133.0. 135.3. 144.6. 164.3; LC-MS (ESI) *m/z* 298 ([M + 1]^+^).

*1-Morpholino-2-[4-(thiophen-3-yl)-1H-1,2,3-triazol-1-yl]ethanone* (**2aad**). Light brown solid; Yield: 92%. ^1^H-NMR (CDCl_3_) δ 3.60–3.62 (m, 2H), 3.65–3.66 (m, 2H), 3.69–3.71 (m, 4H), 5.26 (s, 2H), 7.39 (dd, *J* = 3.0, 5.0 Hz, 1H), 7.46 (dd, *J* = 1.2, 5.0 Hz, 1H), 7.70 (dd, *J* = 1.3, 3.0 Hz, 1H), 7.88 (s, 1H); ^13^C-NMR (DMSO-*d*_6_) δ 41.9, 44.7, 50.6, 65.86, 65.93, 120.6, 122.8, 125.8, 127.1, 132.1, 142.7, 164.4; LC-MS (ESI) *m/z* 279 ([M + 1]^+^).

*1-Morpholino-2-[4-(pyridin-2-yl)-1H-1,2,3-triazol-1-yl]ethanone* (**2aae**). Light brown solid; Yield: 84%. ^1^H-NMR (CDCl_3_) δ 3.55 (t, *J* = 4.8 Hz, 2H), 3.61 (t, *J* = 4.7 Hz, 2H), 3.65–3.68 (m, 4H), 4.11 (s, 2H), 5.15 (s, 2H), 7.22 (m, 1H), 7.25–7.31 (m, 4H), 7.37 (s, 1H); ^13^C-NMR (DMSO-*d*_6_) δ 41.9, 44.7, 50.8, 65.8, 65.9, 119.3, 122.9, 125.0, 137.2, 147.0, 149.6, 150.0, 164.4; LC-MS (ESI) *m/z* 274 ([M + 1]^+^).

*2-[4-(3-Methoxyphenyl)-1H-1,2,3-triazol-1-yl]-1-morpholinoethanone* (**2aag**). White solid; Yield: 94%. ^1^H-NMR (CDCl_3_) δ 3.62 (m, 2H), 3.66 (m, 2H), 3.69–3.71 (m, 4H), 3.87 (s, 3H), 5.27 (s, 2H), 6.89 (ddd, *J* = 1.1, 2.6, 8.1 Hz, 1H), 7.33 (t, *J* = 7.9 Hz, 1H), 7.38 (td, *J* = 1.3, 7.6 Hz, 1H), 7.45 (dd, *J* = 1.5, 2.5 Hz, 1H), 7.97 (s, 1H); ^13^C-NMR (DMSO-*d*_6_) δ 41.9, 44.8, 50.7, 55.1, 65.87, 65.94, 110.3, 113.5, 117.4, 123.3, 130.0, 132.1, 146.0, 159.7, 164.4; LC-MS (ESI) *m/z* 303 ([M + 1]^+^).

*2-[4-(2-Methoxyphenyl)-1H-1,2,3-triazol-1-yl]-1-morpholinoethanone* (**2aah**). White solid; Yield: 93%. ^1^H-NMR (CDCl_3_) δ 3.62 (m, 2H), 3.66-3.69 (m, 6H), 3.94 (s, 3H), 5.27 (s, 2H), 6.98 (dd, *J* = 0.8, 8.3 Hz, 1H), 7.08 (dt, *J* = 1.0, 7.6 Hz, 1H), 7.32 (ddd, *J* = 1.7, 7.4, 8.3 Hz, 1H), 8.23 (s, 1H), 8.34 (dd, *J* = 1.7, 7.7 Hz, 1H); ^13^C-NMR (DMSO-*d*_6_) δ 41.9, 44.7, 50.6, 55.4, 65.8, 65.9, 111.5, 119.1, 120.6, 125.6, 126.4, 128.8, 141.5, 155.3, 164.6; LC-MS (ESI) *m/z* 303 ([M + 1]^+^).

*1-Morpholino-2-(4-p-tolyl-1H-1,2,3-triazol-1-yl)ethanone* (**2aai**). White solid; Yield: 91. ^1^H-NMR (CDCl_3_) δ 2.38 (s, 3H), 3.62 (m, 2H), 3.66 (m, 2H), 3.70 (m, 4H), 5.26 (s, 2H), 7.24 (d, *J* = 7.9 Hz, 2H), 7.73 (d, *J* = 8.2 Hz, 2H), 7.94 (s, 1H); ^13^C-NMR (DMSO-*d*_6_) δ 20.8, 41.9, 44.7, 50.7, 65.86, 65.93, 122.6, 125.0, 128.0, 129.4, 137.1, 146.1, 164.5; LC-MS (ESI) *m/z* 287 ([M + 1]^+^).

*1-Morpholino-2-(4-m-tolyl-1H-1,2,3-triazol-1-yl)ethanone* (**2aaj**). Light yellow solid; Yield: 94%. ^1^H-NMR (CDCl_3_) δ 2.40 (s, 3H), 3.62 (m, 2H), 3.66 (m, 2H), 3.69 (m, 4H), 5.27 (s, 2H), 7.16 (d, *J* = 7.6 Hz, 1H), 7.31 (t, *J* = 7.7 Hz, 1H), 7.61 (d, *J* = 7.7 Hz, 1H), 7.70 (s, 1H), 7.97 (s, 1H); ^13^C-NMR (DMSO-*d*_6_) δ 21.1, 41.9, 44.8, 50.7, 65.88, 65.94, 122.3, 123.0, 125.7, 128.4, 128.8, 130.7, 138.0, 146.2, 164.4; LC-MS (ESI) *m/z* 287 ([M + 1]^+^).

*1-Morpholino-2-(4-o-tolyl-1H-1,2,3-triazol-1-yl)ethanone* (**2aak**). White solid; Yield: 93%. ^1^H-NMR (CDCl_3_) δ 2.49 (s, 3H), 3.62 (m, 2H), 3.66 (m, 2H), 3.69–3.71 (m, 4H), 5.30 (s, 2H), 7.27–7.29 (m, 3H), 7.79 (m, 1H), 7.87 (s, 1H); ^13^C-NMR (DMSO-*d*_6_) δ 21.1, 41.9, 44.7, 50.6, 65.86, 65.94, 125.0, 126.0, 127.7, 128.1, 130.1, 130.9, 134.8, 145.2, 164.5; LC-MS (ESI) *m/z* 287 ([M + 1]^+^).

*2-{4-[4-(Dimethylamino)phenyl]-1H-1,2,3-triazol-1-yl}-1-morpholinoethanone* (**2aal**). Light green solid; Yield: 86%. ^1^H-NMR (CDCl_3_) δ 2.99 (s, 6H), 3.60–3.62 (m, 2H), 3.66–3.70 (m, 6H), 5.24 (s, 2H), 6.77 (d, *J* = 8.9 Hz, 2H), 7.71 (d, *J* = 8.9 Hz, 2H), 7.84 (s, 1H); ^13^C-NMR (DMSO-*d*_6_) δ 40.5, 41.9, 44.8, 50.6, 65.87, 65.94, 112.4, 118.7, 121.2, 126.0, 146.7, 150.0, 164.6; LC-MS (ESI) *m/z* 316 ([M + 1]^+^).

*1-Morpholino-2-[4-(4-phenoxyphenyl)-1H-1,2,3-triazol-1-yl]ethanone* (**2aam**). Yellow solid; Yield: 99%. ^1^H-NMR (CDCl_3_) δ 3.62 (t, *J* = 4.7 Hz, 2H), 3.66 (t, *J* = 4.6 Hz, 2H), 3.70–3.72 (m, 4H), 5.27 (s, 2H), 7.04–7.08 (m, 4H), 7.15 (m, 1H), 7.34-7.38 (m, 2H), 7.80 (d, *J* = 8.8 Hz, 2H), 7.93 (s, 1H); ^13^C-NMR (DMSO-*d*_6_) δ 41.9, 44.8, 50.7, 65.87, 65.94, 118.8, 118.9, 122.7, 123.7, 126.1, 126.9, 130.1, 145.6, 156.36, 156.44, 164.4; LC-MS (ESI) *m/z* 365 ([M + 1]^+^).

*2-[4-(2,4-Difluorophenyl)-1H-1,2,3-triazol-1-yl]-1-morpholinoethanone* (**2aan**). White solid; Yield: 92%. ^1^H-NMR (CDCl_3_) δ 3.60–3.62 (m, 2H), 3.65–3.67 (m, 2H), 3.70–3.73 (m, 4H), 5.29 (s, 2H), 6.90 (ddd, *J* = 2.4, 8.7, 11.0 Hz, 1H), 7.00 (m, 1H), 8.09 (d, J = 3.7 Hz, 1H), 8.27 (dt, *J* = 6.5, 8.6 Hz, 1H); ^13^C-NMR (DMSO-*d*_6_) δ 41.9, 44.7, 50.8, 65.8, 65.9, 104.6 (t, *J*_CF_ = 26.0 Hz), 112.3 (dd, *J*_CF_ = 21.2, 3.6 Hz), 115.3 (dd, *J*_CF_ = 13.3, 3.7 Hz), 125.2 (d, *J*_CF_ = 11.1 Hz), 128.4 (dd, *J*_CF_ = 9.7, 5.3 Hz), 138.8 (d, *J*_CF_ = 2.6 Hz), 158.5 (dd, *J*_CF_ = 250.1, 12.6 Hz), 161.7 (dd, *J*_CF_ = 247.5, 12.6 Hz), 164.4; LC-MS (ESI) *m/z* 309 ([M + 1]^+^).

*2-(4-Benzyl-1H-1,2,3-triazol-1-yl)-1-morpholinoethanone* (**2aao**). White solid; Yield: 94%. ^1^H-NMR (CDCl_3_) δ 3.60 (t, *J* = 4.7 Hz, 2H), 3.66 (t, *J* = 4.6 Hz, 2H), 3.70–3.72 (m, 4H), 5.28 (s, 2H), 7.24 (m, 1H), 7.78 (m, 1H), 8.16 (d, *J* = 7.9 Hz, 1H), 8.32 (s, 1H), 8.59 (d, *J* = 4.2 Hz, 1H); ^13^C-NMR (DMSO-*d*_6_) δ 31.2, 41.9, 44.7, 50.4, 65.8, 65.9, 124.2, 126.1, 128.4, 128.5, 139.7, 145.7, 164.6; LC-MS (ESI) *m/z* 287 ([M + 1]^+^).

*2-(4-Phenyl-1H-1,2,3-triazol-1-yl)-1-(piperidin-1-yl)ethanone* (**2baa**). Light yellow solid; Yield: 99%. ^1^H-NMR (CDCl_3_) δ 1.56–1.62 (m, 4H), 1.68 (m, 2H), 3.52 (t, *J* = 5.5 Hz, 2H), 3.60 (t, *J* = 5.6 Hz, 2H), 5.28 (s, 2H), 7.33 (m, 1H), 7.41–7.44 (m, 2H), 7.84–7.86 (m, 2H), 8.00 (s, 1H); ^13^C-NMR (DMSO-*d*_6_) δ 23.8, 25.1, 25.8, 42.5, 45.2, 50.9, 123.1, 125.1, 127.8, 128.9, 130.9, 146.0, 163.7; LC-MS (ESI) *m/z* 271 ([M + 1]^+^).

*2-[4-(4-Methoxyphenyl)-1H-1,2,3-triazol-1-yl]-1-(piperidin-1-yl)ethanone* (**2bab**). White solid; Yield: 85%. ^1^H-NMR (CDCl_3_) δ 1.56–1.62 (m, 4H), 1.67 (m, 2H), 3.51 (t, *J* = 5.5 Hz, 2H), 3.59 (t, *J* = 5.5 Hz, 2H), 3.84 (s, 3H), 5.28 (s, 2H), 6.96 (d, *J* = 8.7 Hz, 2H), 7.77 (d, *J* = 8.7 Hz, 2H), 7.90 (s, 1H); ^13^C-NMR (DMSO-*d_6_*) δ 23.8, 25.1, 25.8, 42.5, 45.2, 50.8, 55.1, 114.3, 122.1, 123.5, 126.4, 146.0, 158.9, 163.8; LC-MS (ESI) *m/z* 301 ([M + 1]^+^).

*4-{1-[2-Oxo-2-(piperidin-1-yl)ethyl]-1H-1,2,3-triazol-4-yl}benzonitrile (**2bac**). * Yellow solid; Yield: 98%. ^1^H-NMR (CDCl_3_) δ 1.59–1.67 (m, 4H), 1.70 (m, 2H), 3.52 (t, *J* = 5.5 Hz, 2H), 3.60 (t, *J* = 5.6 Hz, 2H), 5.30 (s, 2H), 7.72 (d, *J* = 8.3 Hz, 2H), 7.96 (d, *J* = 8.3 Hz, 2H), 8.10 (s, 1H); ^13^C-NMR (DMSO-*d*_6_) δ 23.8, 25.1, 25.8, 42.5, 45.2, 51.0, 110.0, 118.8, 124.8, 125.6, 133.0, 135.3, 144.5, 163.6; LC-MS (ESI) *m/z* 296 ([M + 1]^+^).

*1-(Piperidin-1-yl)-2-[4-(thiophen-3-yl)-1H-1,2,3-triazol-1-yl]ethanone* (**2bad**). Light brown solid; Yield: 99%. ^1^H-NMR (CDCl_3_) δ 1.57–1.62 (m, 4H), 1.65–1.69 (m, 2H), 3.51 (t, *J* = 5.5 Hz, 2H), 3.59 (t, *J* = 5.6 Hz, 2H), 5.26 (s, 2H), 7.38 (dd, *J* = 3.0, 5.0 Hz, 1H), 7.46 (dd, *J* = 0.9, 5.0 Hz, 1H), 7.69 (dd, *J* = 1.0, 2.9 Hz, 1H), 7.89 (s, 1H); ^13^C-NMR (DMSO-*d*_6_) δ 23.8, 25.1, 25.8, 42.5, 45.2, 50.8, 120.6, 122.9, 125.8, 127.1, 132.2, 142.6, 163.4; LC-MS (ESI) *m/z* 277 ([M + 1]^+^).

*1-(Piperidin-1-yl)-2-[4-(pyridin-2-yl)-1H-1,2,3-triazol-1-yl]ethanone* (**2bae**). White solid; Yield: 90%. ^1^H-NMR (CDCl_3_) δ 1.52–1.58 (m, 4H), 1.61–1.66 (m, 2H), 3.45 (t, *J* = 5.5 Hz, 2H), 3.54 (t, *J* = 5.6 Hz, 2H), 5.26 (s, 2H), 7.19 (ddd, *J* = 1.2, 4.9, 7.5 Hz, 1H), 7.74 (dt, *J* = 1.8, 7.8 Hz, 1H), 8.11 (td, *J* = 1.0, 7.9 Hz, 1H), 8.29 (s, 1H), 8.56 (ddd, *J* = 0.9, 1.7, 4.9 Hz, 1H); ^13^C-NMR (DMSO-*d*_6_) δ 23.8, 25.1, 25.8, 42.5, 45.2, 50.9, 119.3, 122.8, 125.0, 137.2, 146.9, 149.6, 150.1, 163.7; LC-MS (ESI) *m/z* 272 ([M + 1]^+^).

*2-[4-(3-Methoxyphenyl)-1H-1,2,3-triazol-1-yl]-1-(piperidin-1-yl)ethanone* (**2bag**). White solid; Yield: 93%. ^1^H-NMR (CDCl_3_) δ 1.57–1.62 (m, 4H), 1.67 (m, 2H), 3.52 (t, *J* = 5.5 Hz, 2H), 3.60 (t, *J* = 5.6 Hz, 2H), 3.87 (s, 3H), 5.27 (s, 2H), 6.89 (ddd, *J* = 1.0, 2.6, 8.1 Hz, 1H), 7.32 (t, *J* = 7.9 Hz, 1H), 7.38 (td, *J* = 1.3, 7.6 Hz, 1H), 7.46 (dd, *J* = 1.5, 2.5 Hz, 1H), 7.99 (s, 1H); ^13^C-NMR (DMSO-*d*_6_) δ 23.8, 25.1, 25.8, 42.5, 45.2, 50.9, 55.1, 110.2, 113.5, 117.4, 123.4, 130.0, 132.2, 145.9, 159.7, 163.7; LC-MS (ESI) *m/z* 301 ([M + 1]^+^).

*2-[4-(2-Methoxyphenyl)-1H-1,2,3-triazol-1-yl]-1-(piperidin-1-yl)ethanone* (**2bah**). White solid; Yield: 95%. ^1^H-NMR (CDCl_3_) δ 1.52–1.58 (m, 4H), 1.65 (m, 2H), 3.50 (t, *J* = 5.9 Hz, 2H), 3.58 (t, *J* = 5.5 Hz, 2H), 3.92 (s, 3H), 5.26 (s, 2H), 6.97 (dd, *J* = 0.6, 8.3 Hz, 1H), 7.07 (dt, *J* = 1.0, 7.6 Hz, 1H), 7.30 (ddd, *J* = 1.7, 7.4, 8.3 Hz, 1H), 8.23 (s, 1H), 8.34 (dd, *J* = 1.7, 7.7 Hz, 1H); ^13^C-NMR (DMSO-*d*_6_) δ 23.8, 25.1, 25.8, 42.5, 45.2, 50.7, 22.4, 111.5, 119.2, 120.6, 125.6, 126.4, 128.7, 141.4, 155.3, 163.9; LC-MS (ESI) *m/z* 301 ([M + 1]^+^).

*1-(Piperidin-1-yl)-2-(4-p-tolyl-1H-1,2,3-triazol-1-yl)ethanone* (**2bai**). Light yellow solid; Yield: 91%. ^1^H-NMR (CDCl_3_) δ 1.57–1.60 (m, 4H), 1.67 (m, 2H), 2.38 (s, 3H), 3.52 (t, *J* = 5.5 Hz, 2H), 3.59 (t, *J* = 5.6 Hz, 2H), 5.26 (s, 2H), 7.23 (d, *J* = 7.9 Hz, 2H), 7.73 (d, *J* = 8.2 Hz, 2H), 7.95 (s, 1H); ^13^C-NMR (DMSO-*d*_6_) δ 20.8, 23.8, 25.1, 25.8, 42.5, 45.2, 50.8, 122.7, 125.0, 128.1, 129.4, 137.0, 146.1, 163.8; LC-MS (ESI) *m/z* 285 ([M + 1]^+^).

*1-(Piperidin-1-yl)-2-(4-m-tolyl-1H-1,2,3-triazol-1-yl)ethanone* (**2baj**). Light yellow solid; Yield: 99%. ^1^H-NMR (CDCl_3_) δ 1.56–1.62 (m, 4H), 1.67 (m, 2H), 2.40 (s, 3H), 3.51 (t, *J* = 5.5 Hz, 2H), 3.59 (t, *J* = 5.6 Hz, 2H), 5.26 (s, 2H), 7.15 (d, *J* = 7.6 Hz, 1H), 7.31 (t, *J* = 7.6 Hz, 1H), 7.61 (d, *J* = 7.7 Hz, 1H), 7.70 (s, 1H), 7.97 (s, 1H); ^13^C-NMR (DMSO-*d*_6_) δ 21.1, 23.8, 25.1, 25.8, 42.5, 45.2, 50.8, 122.3, 123.0, 125.6, 128.4, 128.8, 130.8, 138.0, 146.1, 163.7; LC-MS (ESI) *m/z* 285 ([M + 1]^+^).

*1-(Piperidin-1-yl)-2-(4-o-tolyl-1H-1,2,3-triazol-1-yl)ethanone* (**2bak**). Light yellow solid; Yield: 91%. ^1^H-NMR (CDCl_3_) δ 1.57–1.61 (m, 4H), 1.68 (m, 2H), 2.49 (s, 3H), 3.53 (t, *J* = 5.5 Hz, 2H), 3.60 (t, *J* = 5.6 Hz, 2H), 5.29 (s, 2H), 7.27–7.28 (m, 3H), 7.79 (m, 1H), 7.88 (s, 1H); ^13^C-NMR (DMSO-*d*_6_) δ 21.1, 23.8, 25.1, 25.8, 42.5, 45.2, 50.8, 125.0, 126.0, 127.7, 128.1, 130.1, 130.9, 134.8, 145.2, 163.8; LC-MS (ESI) *m/z* 285 ([M + 1]^+^).

*2-{4-[4-(Dimethylamino)phenyl]-1H-1,2,3-triazol-1-yl}-1-(piperidin-1-yl)ethanone* (**2bal**). Light green solid; Yield: 89%. ^1^H-NMR (CDCl_3_) δ 1.53-1.57 (m, 4H), 1.64–1.68 (m, 2H), 2.99 (s, 6H), 3.51 (t, *J* = 5.5 Hz, 2H), 3.58 (t, *J* = 5.5 Hz, 2H), 5.24 (s, 2H), 6.77 (d, *J* = 8.9 Hz, 2H), 7.71 (d, *J* = 8.9 Hz, 2H), 7.84 (s, 1H); ^13^C-NMR (DMSO-*d*_6_) δ 23.8, 25.1, 25.8, 40.0, 42.5, 45.2, 50.7, 112.4, 118.8, 121.2, 126.0, 146.6, 149.9, 163.9; LC-MS (ESI) *m/z* 314 ([M + 1]^+^).

*2-[4-(4-Phenoxyphenyl)-1H-1,2,3-triazol-1-yl]-1-(piperidin-1-yl)ethanone* (**2bam**). Light yellow solid; Yield: 99%.^1^H-NMR (CDCl_3_) δ 1.59–1.62 (m, 4H), 1.66–1.71 (m, 2H), 3.52 (t, *J* = 5.5 Hz, 2H), 3.59 (t, *J* = 5.5 Hz, 2H), 5.27 (s, 2H), 7.04–7.07 (m, 4H), 7.13 (m, 1H), 7.34–7.37 (m, 2H), 7.81 (d, *J* = 8.8 Hz, 2H), 7.94 (s, 1H); ^13^C-NMR (DMSO-*d*_6_) δ 23.8, 25.1, 25.8, 42.5, 45.2, 50.8, 118.8, 118.9, 122.7, 123.6, 126.2, 126.8, 130.1, 145.6, 156.3, 156.4, 163.7; LC-MS (ESI) *m/z* 363 ([M + 1]^+^).

*2-[4-(2,4-Difluorophenyl)-1H-1,2,3-triazol-1-yl]-1-(piperidin-1-yl)ethanone* (**2ban**). Yellow solid; Yield: 99%. ^1^H-NMR (CDCl_3_) δ 1.58–1.64 (m, 4H), 1.66–1.71 (m, 2H), 3.51 (t, *J* = 5.5 Hz, 2H), 3.60 (t, *J* = 5.6 Hz, 2H), 5.29 (s, 2H), 6.90 (ddd, *J* = 2.4, 8.7, 11.0 Hz, 1H), 6.99 (m, 1H), 8.09 (d, *J* = 3.7 Hz, 1H), 8.27 (dt, *J* = 6.5, 8.6 Hz, 1H); ^13^C-NMR (DMSO-*d*_6_) δ 23.8, 25.1, 25.7, 42.5, 45.2, 50.9, 104.6 (t, *J*_CF_ = 26.0 Hz), 112.3 (d, *J*_CF_ = 24.6 Hz), 115.3 (dd, *J*_CF_ = 13.1, 4.0 Hz), 125.2 (d, *J*_CF_ = 11.9 Hz), 128.4 (dd, *J*_CF_ = 9.4, 5.7 Hz), 138.7 (d, *J*_CF_ = 2.4 Hz), 158.5 (dd, *J*_CF_ = 250.0, 12.6 Hz), 161.6 (dd, *J*_CF_ = 247.5, 12.6 Hz), 163.7; LC-MS (ESI) *m/z* 307 ([M + 1]^+^).

*2-(4-Benzyl-1H-1,2,3-triazol-1-yl)-1-(piperidin-1-yl)ethanone* (**2bao**). White solid; Yield: 95%. ^1^H-NMR (CDCl_3_) δ 1.52–1.57 (m, 4H), 1.63–1.67 (m, 2H), 3.45 (t, *J* = 5.5 Hz, 2H), 3.56 (t, *J* = 5.6 Hz, 2H), 4.10 (s, 2H), 5.15 (s, 2H), 7.20 (m, 1H), 7.24–7.30 (m, 4H), 7.37 (s, 1H); ^13^C-NMR (DMSO-*d*_6_) δ 23.8, 25.1, 25.7, 31.2, 42.4, 45.2, 50.5, 124.2, 126.1, 128.4, 128.5, 139.7, 145.6, 163.9; LC-MS (ESI) *m/z* 285 ([M + 1]^+^).

*2-(4-Phenyl-1H-1,2,3-triazol-1-yl)-1-(pyrrolidin-1-yl)ethanone* (**2caa**). Light yellow solid; Yield: 94%. ^1^H-NMR (CDCl_3_) δ 1.92 (tt, *J* = 6.7, 7.0 Hz, 2H), 2.05 (tt, *J* = 6.8, 7.0 Hz, 2H), 3.54 (t, *J* = 7.0 Hz, 2H), 3.58 (t, *J* = 6.9 Hz, 2H), 5.19 (s, 2H), 7.33 (m, 1H), 7.41–7.44 (m, 2H), 7.84–7.86 (m, 2H), 8.06 (s, 1H); ^13^C-NMR (DMSO-*d*_6_) δ 23.7, 25.6, 45.1, 45.8, 51.4, 123.0, 125.1, 127.8, 128.9, 130.8, 146.0, 163.7; LC-MS (ESI) *m/z* 257 ([M + 1]^+^).

*2-[4-(4-Methoxyphenyl)-1H-1,2,3-triazol-1-yl]-1-(pyrrolidin-1-yl)ethanone* (**2cab**). White solid; Yield: 89%. ^1^H-NMR (CDCl_3_) δ 1.91 (tt, *J* = 6.8, 7.1 Hz, 2H), 2.03 (tt, *J* = 6.8, 7.0 Hz, 2H), 3.53 (t, *J* = 7.0 Hz, 2H), 3.58 (t, *J* = 6.9 Hz, 2H), 3.84 (s, 3H), 5.17 (s, 2H), 6.96 (d, *J* = 8.9 Hz, 2H), 7.77 (d, *J* = 8.9 Hz, 2H), 7.96 (s, 1H); ^13^C-NMR (DMSO-*d*_6_) δ 23.7, 25.6, 45.1, 45.8, 51.4, 55.1, 114.3, 122.0, 1234, 126.4, 146.0, 158.9, 163.7; LC-MS (ESI) *m/z* 287 ([M + 1]^+^).

*4-{1-[2-Oxo-2-(pyrrolidin-1-yl)ethyl]-1H-1,2,3-triazol-4-yl}benzonitrile* (**2cac**). Yellow solid; Yield: 94%. ^1^H-NMR (CDCl_3_) δ 1.93 (tt, *J* = 6.9, 7.1 Hz, 2H), 2.07 (tt, *J* = 6.8, 6.9 Hz, 2H), 3.54 (t, *J* = 7.0 Hz, 2H), 3.60 (t, *J* = 6.9 Hz, 2H), 5.21 (s, 2H), 7.71 (d, *J* = 8.7 Hz, 2H), 7.96 (d, *J* = 8.7 Hz, 2H), 8.17 (s, 1H); ^13^C- NMR (DMSO-*d*_6_) δ 23.7, 25.6, 45.1, 45.9, 51.5, 110.0, 118.8, 124.7, 125.7, 133.0, 135.3, 144.5, 163.5; LC-MS (ESI) *m/z* 282 ([M + 1]^+^).

*1-(Pyrrolidin-1-yl)-2-[4-(thiophen-3-yl)-1H-1,2,3-triazol-1-yl]ethanone* (**2cad**). Light green solid; Yield: 88%. ^1^H-NMR (CDCl_3_) δ 1.91 (tt, *J* = 6.7, 6.9 Hz, 2H), 2.05 (tt, *J* = 6.8, 6.9 Hz, 2H), 3.53 (t, *J* = 7.0 Hz, 2H), 3.58 (t, *J* = 6.9 Hz, 2H), 5.18 (s, 2H), 7.38 (dd, *J* = 3.0, 5.0 Hz, 1H), 7.47 (dd, *J* = 1.2, 5.0 Hz, 1H), 7.69 (dd, *J* = 1.3, 3.0 Hz, 1H), 7.96 (s, 1H); ^13^C-NMR (DMSO-*d*_6_) δ 23.7, 25.6, 45.1, 45.8, 51.3, 120.6, 122.7, 125.8, 127.1, 132.2, 142.6, 163.7; LC-MS (ESI) *m/z* 263 ([M + 1]^+^).

*2-[4-(Pyridin-2-yl)-1H-1,2,3-triazol-1-yl]-1-(pyrrolidin-1-yl)ethanone* (**2cae**). Light yellow solid; Yield: 87%. ^1^H-NMR (CDCl_3_) δ 1.89 (tt, *J* = 6.9, 7.0 Hz, 2H), 2.02 (tt, *J* = 6.8, 6.9 Hz, 2H), 3.52 (t, *J* = 6.9 Hz, 2H), 3.54 (t, *J* = 6.8 Hz, 2H), 5.20 (s, 2H), 7.21 (ddd, *J* = 1.2, 4.9, 7.5 Hz, 1H), 7.76 (dt, *J* = 1.8, 7.7 Hz, 1H), 8.14 (td, *J* = 1.0, 7.9 Hz, 1H), 8.35 (s, 1H), 8.58 (ddd, *J* = 0.9, 1.7, 4.9 Hz, 1H); ^13^C-NMR (DMSO-*d*_6_) δ 23.7, 25.6, 45.2, 45.9, 51.5, 119.4, 122.9, 124.8, 137.2, 146.9, 149.6, 150.1, 163.6; LC-MS (ESI) *m/z* 258 ([M + 1]^+^).

*2-[4-(3-Methoxyphenyl)-1H-1,2,3-triazol-1-yl]-1-(pyrrolidin-1-yl)ethanone* (**2cag**). White solid; Yield: 93%. ^1^H-NMR (CDCl_3_) δ 1.92 (tt, *J* = 6.8, 6.8 Hz, 2H), 2.05 (tt, *J* = 6.8, 6.9 Hz, 2H), 3.54 (t, *J* = 7.0 Hz, 2H), 3.58 (t, *J* = 6.9 Hz, 2H), 3.87 (s, 3H), 5.19 (s, 2H), 6.88 (ddd, *J* = 1.0, 2.6, 8.2 Hz, 1H), 7.32 (t, *J* = 7.9 Hz, 1H), 7.39 (td, *J* = 1.3, 7.6 Hz, 1H), 7.46 (dd, *J* = 1.6, 2.5 Hz, 1H), 8.05 (s, 1H); ^13^C-NMR (DMSO-*d*_6_) δ 23.7, 25.6, 45.2, 45.8, 51.4, 55.1, 110.3, 113.5, 117.5, 123.2, 130.1, 132.2, 146.0, 159.7, 163.7; LC-MS (ESI) *m/z* 287 ([M + 1]^+^).

*2-[4-(2-Methoxyphenyl)-1H-1,2,3-triazol-1-yl]-1-(pyrrolidin-1-yl)ethanone* (**2cah**). White solid; Yield: 89%. ^1^H-NMR (CDCl_3_) δ 1.90 (tt, *J* = 6.8, 6.9 Hz, 2H), 2.02 (tt, *J* = 6.8, 6.8 Hz, 2H), 3.53 (t, *J* = 7.0 Hz, 2H), 3.56 (t, *J* = 6.9 Hz, 2H), 3.93 (s, 3H), 5.19 (s, 2H), 6.72 (dd, *J* = 0.7, 8.3 Hz, 1H), 7.07 (dt, *J* = 1.0, 7.5 Hz, 1H), 7.31 (ddd, *J* = 1.7, 7.4, 8.3 Hz, 1H), 8.29 (s, 1H), 8.34 (dd, *J* = 1.7, 7.7 Hz, 1H); ^13^C- NMR (DMSO-*d*_6_) δ 23.7, 25.6, 45.1, 45.8, 51.2, 55.5, 111.5, 119.2, 120.7, 125.5, 126.4, 128.8, 141.5, 155.3, 163.9; LC-MS (ESI) *m/z* 287 ([M + 1]^+^).

*1-(Pyrrolidin-1-yl)-2-(4-p-tolyl-1H-1,2,3-triazol-1-yl)ethanone* (**2cai**). White solid; Yield: 96%. ^1^H-NMR (CDCl_3_) δ 1.91 (tt, *J* = 6.9, 6.9 Hz, 2H), 2.04 (tt, *J* = 6.8, 6.8 Hz, 2H), 2.38 (s, 3H), 3.53 (t, *J* = 7.0 Hz, 2H), 3.57 (t, *J* = 6.9 Hz, 2H), 5.18 (s, 2H), 7.23 (d, *J* = 8.0 Hz, 2H), 7.73 (d, *J* = 8.1 Hz, 2H), 8.01 (s, 1H); ^13^C-NMR (DMSO-*d*_6_) δ 20.8, 23.7, 25.6, 45.2, 45.8 51.4, 122.5, 125.0, 128.1, 129.5, 137.1, 146.1, 163.7; LC-MS (ESI) *m/z* 271 ([M + 1]^+^).

*1-(Pyrrolidin-1-yl)-2-(4-m-tolyl-1H-1,2,3-triazol-1-yl)ethanone* (**2caj**). White solid; Yield: 96%. ^1^H-NMR (CDCl_3_) δ 1.91 (tt, *J* = 6.8, 6.8 Hz, 2H), 2.03 (tt, *J* = 6.8, 6.8 Hz, 2H), 2.40 (s, 3H), 3.53 (t, *J* = 7.0 Hz, 2H), 3.57 (t, *J* = 6.8 Hz, 2H), 5.18 (s, 2H), 7.14 (d, *J* = 7.6 Hz, 1H), 7.30 (t, *J* = 7.7 Hz, 1H), 7.61 (d, *J* = 7.7 Hz, 1H), 7.70 (s, 1H), 8.03 (s, 1H); ^13^C-NMR (DMSO-*d*_6_) δ 21.1, 23.7, 25.6, 45.1, 45.8, 51.4, 122.3, 122.9, 125.6, 128.4, 128.8, 130.7, 138.0, 146.1, 163.7; LC-MS (ESI) *m/z* 271 ([M + 1]^+^).

*1-(Pyrrolidin-1-yl)-2-(4-o-tolyl-1H-1,2,3-triazol-1-yl)ethanone* (**2cak**). White solid; Yield: 91%. ^1^H-NMR (CDCl_3_) δ 1.92 (tt, *J* = 6.8, 6.9 Hz, 2H), 2.05 (tt, *J* = 6.8, 6.8 Hz, 2H), 2.49 (s, 3H), 3.54 (t, *J* = 7.0 Hz, 2H), 3.59 (t, *J* = 6.9 Hz, 2H), 5.21 (s, 2H), 7.26–7.28 (m, 3H), 7.79 (m, 1H), 7.94 (s, 1H); ^13^C-NMR (DMSO-*d*_6_) δ 21.1, 23.7, 25.6, 45.1, 45.8, 51.3, 124.8, 126.0, 127.7, 128.1, 130.1, 130.9, 134.8, 145.2, 163.7; LC-MS (ESI) *m/z* 271 ([M + 1]^+^).

*2-{4-[4-(Dimethylamino)phenyl]-1H-1,2,3-triazol-1-yl}-1-(pyrrolidin-1-yl)ethanone* (**2cal**). Light yellow solid; Yield: 94%. ^1^H-NMR (CDCl_3_) δ 1.90 (tt, *J* = 6.7, 6.9 Hz, 2H), 2.02 (tt, *J* = 6.8, 6.9 Hz, 2H), 2.99 (s, 6H), 3.52 (t, *J* = 7.0 Hz, 2H), 3.56 (t, *J* = 6.9 Hz, 2H), 5.16 (s, 2H), 6.77 (d, *J* = 9.0 Hz, 2H), 7.71 (d, *J* = 8.9 Hz, 2H), 7.90 (s, 1H); ^13^C-NMR (DMSO-*d*_6_) δ 23.7, 25.6, 40.0, 45.1, 45.8, 51.3, 112.4, 118.8, 121.1, 126.0, 146.6, 150.0, 163.8; LC-MS (ESI) *m/z* 300 ([M + 1]^+^).

*2-[4-(4-Phenoxyphenyl)-1H-1,2,3-triazol-1-yl]-1-(pyrrolidin-1-yl)ethanone* (**2cam**). Light yellow solid; Yield: 99%. ^1^H-NMR (CDCl_3_) δ 1.92 (tt, *J* = 6.8, 6.9 Hz, 2H), 2.05 (tt, *J* = 6.8, 6.9 Hz, 2H), 3.53 (t, *J* = 7.0 Hz, 2H), 3.58 (t, *J* = 6.9 Hz, 2H), 5.19 (s, 2H), 7.04–7.07 (m, 4H), 7.12 (m, 1H), 7.34–7.37 (m, 2H), 7.81 (d, *J* = 8.8 Hz, 2H), 8.00 (s, 1H); ^13^C-NMR (DMSO-*d*_6_) δ 23.7, 25.6, 45.1, 45.8, 51.4, 118.8, 118.9, 122.6, 123.6, 126.2, 126.8, 130.1, 145.6, 156.3, 156.4, 163.7; LC-MS (ESI) *m/z* 349 ([M + 1]^+^).

*2-[4-(2,4-Difluorophenyl)-1H-1,2,3-triazol-1-yl]-1-(pyrrolidin-1-yl)ethanone* (**2can**). Light yellow solid; Yield: 99%. ^1^H-NMR (CDCl_3_) δ. 1.92 (tt, *J* = 6.7, 6.9 Hz, 2H), 2.05 (tt, *J* = 6.8, 6.9 Hz, 2H), 3.53 (t, *J* = 7.0 Hz, 2H), 3.58 (t, *J* = 6.9 Hz, 2H), 5.20 (s, 2H), 6.90 (ddd, *J* = 2.4, 8.7, 11.0 Hz, 1H), 6.99 (m, 1H), 8.14 (d, *J* = 3.7 Hz, 1H), 8.27 (dt, *J* = 6.5, 8.6 Hz, 1H); ^13^C-NMR (DMSO-*d*_6_) δ 23.7, 25.6, 45.1, 45.8, 51.4, 104.6 (t, *J*_CF_ = 26.0 Hz), 112.3 (dd, *J*_CF_ = 20.2, 4.8 Hz), 115.3 (dd, *J*_CF_ = 13.4, 3.8 Hz), 125.0 (d, *J*_CF_ = 11.1 Hz), 128.5 (dd, *J*_CF_ = 9.8, 5.3 Hz),138.7, 158.5 (dd, *J*_CF_ = 250.0, 12.6 Hz), 161.7 (dd, *J*_CF_ = 247.4, 12.7 Hz), 163.6; LC-MS (ESI) *m/z* 293 ([M + 1]^+^).

*2-(4-Benzyl-1H-1,2,3-triazol-1-yl)-1-(pyrrolidin-1-yl)ethanone* (**2cao**). White solid; Yield: 94%. ^1^H-NMR (CDCl_3_) δ 1.86 (tt, *J* = 6.8, 7.0 Hz, 2H), 1.99 (tt, *J* = 6.8, 7.0 Hz, 2H), 3.46 (t, *J* = 7.0 Hz, 2H), 3.49 (t, *J* = 6.9 Hz, 2H), 4.08 (s, 2H), 5.05 (s, 2H), 7.19 (m, 1H), 7.24–7.29 (m, 4H), 7.42 (s, 1H); ^13^C-NMR (DMSO-*d*_6_) δ 23.7, 25.6, 31.2, 45.1, 45.7, 51.1, 124.0, 126.1, 128.4, 128.5, 139.7, 145.6, 163.8; LC-MS (ESI) *m/z* 271 ([M + 1]^+^).

*1-(Azepan-1-yl)-2-(4-phenyl-1H-1,2,3-triazol-1-yl)ethanone* (**2daa**). Light yellow solid; Yield: 95%. ^1^H-NMR (CDCl_3_) δ 1.58–1.63 (m, 4H), 1.72-1.77 (m, 2H), 1.78–1.83 (m, 2H), 3.57 (t, *J* = 6.0 Hz, 2H), 3.58 (t, *J* = 6.0 Hz, 2H), 5.27 (s, 2H), 7.33 (m, 1H), 7.41–7.44 (m, 2H), 7.84–7.86 (m, 2H), 8.04 (s, 1H); ^13^C-NMR (DMSO-*d*_6_) δ 26.2, 26.7, 27.1, 28.2, 45.5, 46.4, 50.7, 123.2, 125.1, 277, 128.9, 130.9, 146.0, 165.0; LC-MS (ESI) *m/z* 285 ([M + 1]^+^).

*1-(Azepan-1-yl)-2-[4-(4-methoxyphenyl)-1H-1,2,3-triazol-1-yl]ethanone* (**2dab**). White solid; Yield: 86%. ^1^H-NMR (CDCl_3_) δ 1.59-1.63 (m, 4H), 1.72-1.76 (m, 2H), 1.77–1.82 (m, 2H), 3.53–3.59 (m, 4H), 3.84 (s, 3H), 5.25 (s, 2H), 6.95 (d, *J* = 8.9 Hz, 2H), 7.77 (d, *J* = 8.8 Hz, 2H), 7.95 (s, 1H); ^13^C-NMR (DMSO-*d*_6_) δ 26.2, 26.7, 27.1, 28.2, 45.5, 46.4, 50.7, 55.1, 114.3, 122.2, 123.5, 126.4, 145.9, 158.9, 165.0; LC-MS (ESI) *m/z* 315 ([M + 1]^+^).

*4-{1-[2-(Azepan-1-yl)-2-oxoethyl]-1H-1,2,3-triazol-4-yl}benzonitrile* (**2dac**). Yellow solid; Yield: 97%. ^1^H-NMR (CDCl_3_) δ 1.57–1.66 (m, 4H), 1.75 (tt, *J* = 5.9, 6.0 Hz, 2H), 1.84 (tt, *J* = 5.9, 6.0 Hz, 2H), 3.56–3.60 (m, 4H), 5.29 (s, 2H), 7.71 (d, *J* = 6.7 Hz, 2H), 7.96 (d, *J* = 6.7 Hz, 2H), 8.15 (s, 1H); ^13^C-NMR (DMSO-*d*_6_) δ 26.2, 26.4, 27.0, 28.1, 45.5, 46.4, 50.9, 110.0, 118.8, 124.9, 125.6, 133.0, 133.3, 144.5, 164.8; LC-MS (ESI) *m/z* 310 ([M + 1]^+^).

*1-(Azepan-1-yl)-2-[4-(thiophen-3-yl)-1H-1,2,3-triazol-1-yl]ethanone* (**2dad**). Light brown solid; Yield: 98%. ^1^H-NMR (CDCl_3_) δ 1.58–1.61 (m, 4H), 1.75 (tt, *J* = 5.8, 5.8 Hz, 2H), 1.80 (tt, *J* = 5.8, 5.9 Hz, 2H), 3.57 (t, *J* = 6.0 Hz, 2H), 3.58 (t, *J* = 5.9 Hz, 2H), 5.26 (s, 2H), 7.37 (dd, *J* = 3.0, 5.0 Hz, 1H), 7.47 (dd, *J* = 1.3, 5.0 Hz, 1H), 7.69 (dd, *J* = 1.2, 3.0 Hz, 1H), 7.94 (s, 1H); ^13^C-NMR (DMSO-*d*_6_) δ 26.2, 26.7, 27.1, 28.2, 45.5, 46.4, 50.7, 120.6, 122.9, 125.8, 127.0, 132.2, 142.6, 165.0; LC-MS (ESI) *m/z* 291 ([M + 1]^+^).

*1-(Azepan-1-yl)-2-[4-(pyridin-2-yl)-1H-1,2,3-triazol-1-yl]ethanone* (**2dae**). Light brown solid; Yield: 86%. ^1^H-NMR (CDCl_3_) δ 1.59–1.64 (m, 4H), 1.74 (tt, *J* = 5.9, 6.0 Hz, 2H), 1.80 (tt, *J* = 5.8, 6.0 Hz, 2H), 3.55 (t, *J* = 6.1 Hz, 2H), 3.57 (t, *J* = 6.0 Hz, 2H), 5.28 (s, 2H), 7.22 (ddd, *J* = 1.1, 4.9, 7.5 Hz, 1H), 7.76 (dt, *J* = 1.8, 7.7 Hz, 1H), 8.15 (d, *J* = 7.9 Hz, 1H), 8.34 (s, 1H), 8.59 (d, *J* = 4.3 Hz, 1H); ^13^C-NMR (DMSO-*d_6_*) δ 26.2, 26.7, 27.0, 28.2, 45.5, 46.4, 50.8, 119.3, 122.9, 125.1, 137.2, 146.9, 149.6, 150.1, 164.9; LC-MS (ESI) *m/z* 286 ([M + 1]^+^).

*1-(Azepan-1-yl)-2-[4-(3-methoxyphenyl)-1H-1,2,3-triazol-1-yl]ethanone* (**2dag**). Light yellow solid; Yield: 92%. ^1^H-NMR (CDCl_3_) δ 1.58–1.61 (m, 4H), 1.75 (m, 2H), 1.80 (m, 2H), 3.56–3.59 (m, 4H), 3.87 (s, 3H), 5.27 (s, 2H), 6.88 (ddd, *J* = 1.0, 2.6, 8.2 Hz, 1H), 7.32 (t, *J* = 7.9 Hz, 1H), 7.39 (td, *J* = 1.2, 7.6 Hz, 1H), 7.46 (dd, *J* = 1.5, 2.5 Hz, 1H), 8.03 (s, 1H); ^13^C-NMR (DMSO-*d*_6_) δ 26.2, 26.7, 27.1, 28.2, 45.5, 46.4, 50.8, 55.1, 110.2, 113.5, 117.4, 123.4, 130.0, 132.2, 145.9, 159.7, 165.0; LC-MS (ESI) *m/z* 315 ([M + 1]^+^).

*1-(Azepan-1-yl)-2-[4-(2-methoxyphenyl)-1H-1,2,3-triazol-1-yl]ethanone* (**2dah**). White solid; Yield: 88%. ^1^H-NMR (CDCl_3_) δ 1.55–1.61 (m, 4H), 1.72–1.78 (m, 4H), 3.56–3.59 (m, 4H), 3.93 (s, 3H), 5.27 (s, 2H), 6.97 (dd, *J* = 0.7, 8.3 Hz, 1H), 7.07 (dt, *J* = 1.0, 7.6 Hz, 1H), 7.31 (ddd, *J* = 1.8, 7.4, 8.3 Hz, 1H), 8.27 (s, 1H), 8.34 (dd, *J* = 1.7, 7.7 Hz, 1H); ^13^C-NMR (DMSO-*d*_6_) δ 26.2, 26.7, 27.1, 28.1, 45.4, 46.4, 50.5, 55.5, 111.5, 119.2, 120.6, 125.7, 126.4, 128.7, 141.4, 155.3, 165.2; LC-MS (ESI) *m/z* 315 ([M + 1]^+^).

*1-(Azepan-1-yl)-2-(4-p-tolyl-1H-1,2,3-triazol-1-yl)ethanone* (**2dai**). White solid; Yield: 97%. ^1^H-NMR (CDCl_3_) δ 1.55–1.62 (m, 4H), 1.74 (m, 2H), 1.79 (m, 2H), 2.38 (s, 3H), 3.55–3.59 (m, 4H), 5.26 (s, 2H), 7.23 (d, *J* = 7.9 Hz, 2H), 7.73 (d, *J* = 8.1 Hz, 2H), 7.99 (s, 1H); ^13^C-NMR (DMSO-*d*_6_) δ 20.8, 26.2, 26.7, 27.1, 28.2, 45.5, 46.5, 50.7, 122.7, 125.0, 128.1, 129.4, 137.0, 146.1, 165.0; LC-MS (ESI) *m/z* 299 ([M + 1]^+^).

*1-(Azepan-1-yl)-2-(4-m-tolyl-1H-1,2,3-triazol-1-yl)ethanone* (**2daj**). White solid; Yield: 91%. ^1^H-NMR (CDCl_3_) δ 1.56–1.62 (m, 4H), 1.74 (m, 2H), 1.79 (m, 2H), 2.40 (s, 3H), 3.55–3.59 (m, 4H), 5.26 (s, 2H), 7.15 (dd, *J* = 0.6, 7.6 Hz, 1H), 7.30 (t, *J* = 7.7 Hz, 1H), 7.62 (d, *J* = 7.8 Hz, 1H), 7.71 (s, 1H), 8.02 (s, 1H); ^13^C-NMR (DMSO-*d*_6_) δ 21.1, 26.2, 26.7, 27.0, 28.2, 45.5, 46.5, 50.7, 122.2, 123.1, 125.6, 128.4, 128.8, 130.8, 138.0, 146.1, 165.0; LC-MS (ESI) *m/z* 299 ([M + 1]^+^).

*1-(Azepan-1-yl)-2-(4-o-tolyl-1H-1,2,3-triazol-1-yl)ethanone* (**2dak**). Light yellow solid; Yield: 86%. ^1^H-NMR (CDCl_3_) δ 1.58–1.63 (m, 4H), 1.75 (m, 2H), 1.80 (m, 2H), 2.49 (s, 3H), 3.57–3.60 (m, 4H), 5.29 (s, 2H), 7.26–7.28 (m, 3H), 7.78 (m, 1H), 7.92 (s, 1H); ^13^C-NMR (DMSO-*d*_6_) δ 21.1, 26.2, 26.7, 27.1, 28.2, 45.5, 46.4, 50.7, 125.1, 126.0, 127.7, 128.1, 130.1, 130.9, 134.8, 145.1, 165.0; LC-MS (ESI) *m/z* 299 ([M + 1]^+^).

*1-(Azepan-1-yl)-2-{4-[4-(dimethylamino)phenyl]-1H-1,2,3-triazol-1-yl}ethanone* (**2dal**). Light brown solid; Yield: 89%. ^1^H-NMR (CDCl_3_) δ 1.57–1.58 (m, 4H), 1.73–1.77 (m, 4H), 2.99 (s, 6H), 3.56 (t, *J* = 6.0 Hz, 2H), 3.57 (t, *J* = 6.0 Hz, 2H), 5.23 (s, 2H), 6.77 (d, *J* = 8.9 Hz, 2H), 7.71 (d, *J* = 8.9 Hz, 2H), 7.89 (s, 1H); ^13^C-NMR (DMSO-*d*_6_) δ 26.2, 26.7, 27.1, 28.2, 40.0, 45.5, 46.5, 50.6, 112.4, 118.8, 121.3, 126.0, 146.6, 150.0, 165.1; LC-MS (ESI) *m/z* 328 ([M + 1]^+^).

*1-(Azepan-1-yl)-2-[4-(4-phenoxyphenyl)-1H-1,2,3-triazol-1-yl]ethanone* (**2dam**). Light yellow solid; Yield: 99%. ^1^H-NMR (CDCl_3_) δ 1.59–1.66 (m, 4H), 1.73–1.77 (m, 2H), 1.7–1.83 (m, 2H), 3.57 (t, *J* = 6.0 Hz, 2H), 3.58 (t, *J* = 6.0 Hz, 2H), 5.27 (s, 2H), 7.04–7.06 (m, 4H), 7.12 (m, 1H), 7.34–7.37 (m, 2H), 7.81 (d, *J* = 8.9 Hz, 2H), 7.99 (s, 1H); ^13^C-NMR (DMSO-*d*_6_) δ 26.2, 26.7, 27.1, 28.2, 45.5, 46.5, 50.7, 118.8, 118.9 ,122.8, 123.6, 126.2, 126.8, 130.1, 145.6, 456.3, 156.4, 165.0; LC-MS (ESI) *m/z* 377 ([M + 1]^+^).

*1-(Azepan-1-yl)-2-[4-(2,4-difluorophenyl)-1H-1,2,3-triazol-1-yl]ethanone* (**2dan**). Yellow solid; Yield: 98%. ^1^H-NMR (CDCl_3_) δ 1.57–1.65 (m, 4H), 1.75 (tt, *J* = 5.8, 5.9 Hz, 2H), 1.82 (tt, *J* = 5.8, 5.9 Hz, 2H), 3.56 (t, *J* = 6.1 Hz, 2H), 3.58 (t, *J* = 6.1 Hz, 2H), 5.28 (s, 2H), 6.90 (ddd, *J* = 2.4, 8.7, 11.0 Hz, 1H), 6.99 (dddd, *J* = 1.0, 2.5, 8.0, 10.4 Hz, 1H), 8.13 (d, *J* = 3.7 Hz, 1H), 8.27 (dt, *J* = 6.5, 8.6 Hz, 1H); ^13^C-NMR (DMSO-*d*_6_) δ 26.2, 26.7, 27.1, 28.1, 45.5, 46.4, 50.8, 104.6 (t, *J*_CF_ = 26.0 Hz), 112.3 (dd, *J*_CF_ = 20.2, 4.8 Hz), 115.3 (dd, *J*_CF_ = 13.2, 3.7 Hz), 125.3 (d, *J*_CF_ = 10.9 Hz), 128.5 (dd, *J*_CF_ = 9.7, 5.4 Hz), 138.7 (d, *J*_CF_ = 2.4 Hz), 158.5 (dd, *J*_CF_ = 249.9, 12.5 Hz), 161.7 (dd, *J*_CF_ = 247.4, 12.7 Hz), 165.0; LC-MS (ESI) *m/z* 321 ([M + 1]^+^).

*1-(Azepan-1-yl)-2-(4-benzyl-1H-1,2,3-triazol-1-yl)ethanone* (**2dao**). White solid; Yield: 94%. ^1^H-NMR (CDCl_3_) δ 1.55–1.57 (m, 4H), 1.68–1.75 (m, 4H), 3.50 (t, *J* = 6.1 Hz, 2H), 3.52 (t, *J* = 6.1 Hz, 2H), 4.10 (s, 2H), 5.15 (s, 2H), 7.21 (m, 1H), 7.26–7.30 (m, 4H), 7.41 (s, 1H); ^13^C-NMR (DMSO-*d*_6_) δ 26.2, 26.7, 27.1, 28.1, 31.3, 45.5, 46.5, 50.5, 124.3, 126.1, 128.4, 128.6, 139.7, 145.7, 165.1; LC-MS (ESI) *m/z* 299 ([M+1]^+^).

*N-Methyl-N-phenyl-2-(4-phenyl-1H-1,2,3-triazol-1-yl)acetamide* (**2eaa**). White solid; Yield: 99%. ^1^H-NMR (CDCl_3_) δ 3.35 (s, 3H), 4.97 (s, 2H), 7.31–7.34 (m, 3H), 7.40–7.43 (m, 2H), 7.45 (m, 1H), 7.51–7.54 (m, 2H), 7.82–7.84 (m, 2H), 7.99 (s, 1H); ^13^C-NMR (DMSO-*d_6_*) δ 37.3, 51.1, 123.0, 125.1, 127.5, 127.8, 128.4, 129.0, 130.1, 130.8, 141.9, 146.0, 164.9; LC-MS (ESI) *m/z* 293 ([M + 1]^+^).

*2-[4-(4-Methoxyphenyl)-1H-1,2,3-triazol-1-yl]-N-methyl-N-phenylacetamide* (**2eab**). Yellow solid; Yield: 99%. ^1^H-NMR (CDCl_3_) δ 3.35 (s, 3H), 3.84 (s, 3H), 4.95 (s, 2H), 6.95 (d, *J* = 8.9 Hz, 2H), 7.30–7.32 (m, 2H), 7.45 (m, 1H), 7.50-7.53 (m, 2H), 7.75 (d, *J* = 8.9 Hz, 2H), 7.89 (s, 1H); ^13^C-NMR (DMSO-*d*_6_) δ 37.3, 51.0, 55.2, 114.3, 122.0, 123.4, 126.5, 127.5, 128.4, 130.1, 141.9, 146.0, 159.0, 164.9; LC-MS (ESI) *m/z* 323 ([M + 1]^+^).

*2-[4-(4-Cyanophenyl)-1H-1,2,3-triazol-1-yl]-N-methyl-N-phenylacetamide* (**2eac**). White solid; Yield: 99%. ^1^H-NMR (CDCl_3_) δ 3.35 (s, 3H), 4.98 (s, 2H), 7.30–7.34 (m, 2H) ,7.47 (m, 1H), 7.56–7.51 (m, 2H), 7.70 (d, *J* = 8.6 Hz, 2H), 7.94 (d, *J* = 8.6 Hz, 2H), 8.10 (s, 1H); ^13^C-NMR (CDCl_3_) δ 37.9, 51.4, 111.4, 118.9, 122.9, 126.2, 127.3, 129.2, 130.6, 132.7, 135.1, 141.7, 146.0, 164.7; LC-MS (ESI) *m/z* 318 ([M + 1]^+^).

*N-Methyl-N-phenyl-2-[4-(thiophen-3-yl)-1H-1,2,3-triazol-1-yl]acetamide* (**2ead**). White solid; Yield: 89%. ^1^H-NMR (CDCl_3_) δ 3.34 (s, 3H), 4.97 (s, 2H), 7.21 (ddd, *J* = 1.1, 4.9, 7.5 Hz, 1H), 7.32 (d, *J* = 7.3 Hz, 2H), 7.45 (t, *J* = 7.4 Hz, 1H), 7.51 (t, *J* = 7.6 Hz, 2H), 7.75 (dt, *J* = 1.8, 7.7 Hz, 1H), 8.13 (d, *J* = 7.9 Hz, 1H), 8.29 (s, 1H), 8.58 (ddd, *J* = 4.9, 1.6, 0.9 Hz, 1H; ^13^C-NMR (CDCl_3_) δ 37.9, 51.3, 121.1, 121.4, 126.0, 126.3, 127.4, 129.1, 130.6, 132.0, 141.9, 144.1, 164.9; LC-MS (ESI) *m/z* 294 ([M + 1]^+^).

*N-Methyl-N-phenyl-2-[4-(pyridin-2-yl)-1H-1,2,3-triazol-1-yl]acetamide* (**2eae**). White solid; Yield: 99%. ^1^H-NMR (CDCl_3_) δ 3.34 (s, 3H), 4.94 (s, 2H), 7.30 (d, *J* = 7.4 Hz, 2H), 7.36 (ddd, *J* = 5.0, 3.0, 0.6 Hz, 1H), 7.42–7.48 (m, 2H), 7.48–7.54 (m, 2H), 7.67 (m, 1H), 7.88 (s, 1H); ^13^C-NMR (CDCl_3_) δ 37.9, 51.4, 120.3, 122.8, 124.0, 127.4, 129.1, 130.6, 136.8, 142.0, 148.6, 149.5, 150.4, 164.7; LC-MS (ESI) *m/z* 299 ([M + 1]^+^).

*2-[4-(3-Methoxyphenyl)-1H-1,2,3-triazol-1-yl)-N-methyl-N-phenylacetamide* (**2eag**). White solid; Yield: 93%. ^1^H-NMR (CDCl_3_) δ 3.35 (s, 3H), 3.86 (s, 3H), 4.96 (s, 2H), 6.88 (ddd, *J* = 1.1, 2.6, 8.1 Hz, 1H), 7.30–7.33 (m, 3H), 7.37 (td, *J* = 1.3, 7.6 Hz, 1H), 7.44 (dd, *J* = 1.5, 2.6 Hz, 1H), 7.45 (m, 1H), 7.50–7.53 (m, 2H), 7.98 (s, 1H); ^13^C-NMR (DMSO-*d*_6_) δ 37.3, 51.1, 55.1, 110.3, 113.6, 117.5, 123.2, 127.5, 128.4, 130.1, 132.1 (2C), 141.9, 146.0, 159.7, 164.9; LC-MS (ESI) *m/z* 315 ([M + 1]^+^).

*2-[4-(2-Methoxyphenyl)-1H-1,2,3-triazol-1-yl)-N-methyl-N-phenylacetamide* (**2eah**). Light yellow solid; Yield: 95%. ^1^H-NMR (CDCl_3_) δ 3.35 (s, 3H), 3.93 (s, 3H), 4.97 (s, 2H), 6.97 (d, *J* = 8.3 Hz, 1H), 7.06 (dt, *J* = 0.9, 7.5 Hz, 1H), 7.28–7.33 (m, 3H), 7.45 (m, 1H), 7.50–7.53 (m, 2H), 8.22 (s, 1H), 8.33 (dd, *J* = 1.7, 7.7 Hz, 1H); ^13^C-NMR (DMSO-*d*_6_) δ 37.5, 51.2, 55.7, 111.8, 119.2, 120.9, 125.9, 126.7, 127.7, 128.7, 129.2, 130.3, 141.7, 142.1, 155.5, 165.3; LC-MS (ESI) *m/z* 315 ([M + 1]^+^).

*N-Methyl-N-phenyl-2-(4-p-tolyl-1H-1,2,3-triazol-1-yl)acetamide* (**2eai**). White solid; Yield: 99%. ^1^H-NMR (CDCl_3_) δ 2.37 (s, 3H), 3.35 (s, 3H), 4.95 (s, 2H), 7.22 (d, *J* = 7.9 Hz, 2H), 7.30–7.32 (m, 2H), 7.45 (m, 1H), 7.50–7.53 (m, 2H), 7.72 (d, *J* = 8.2 Hz, 2H), 7.94 (s, 1H); ^13^C-NMR (DMSO-*d*_6_) δ 20.8, 37.2, 51.0, 122.5, 125.0, 127.4, 128.0, 128.4, 129.4, 130.0, 137.1, 141.9, 146.0, 164.9; LC-MS (ESI) *m/z* 299 ([M + 1]^+^).

*N-Methyl-N-phenyl-2-(4-m-tolyl-1H-1,2,3-triazol-1-yl)acetamide* (**2eaj**). White solid; Yield: 87%. ^1^H-NMR (CDCl_3_) δ 2.39 (s, 3H), 3.33 (s, 3H), 4.95 (s, 2H), 7.13 (d, *J* = 7.6 Hz, 1H), 7.27–7.31 (m, 3H), 7.44 (m, 1H), 7.49–7.52 (m, 2H), 7.59 (d, *J* = 7.8 Hz, 1H), 7.69 (s, 1H), 7.96 (s, 1H); ^13^C-NMR (DMSO-*d*_6_) δ 21.0, 37.3, 51.0, 122.3, 122.9, 125.6, 127.4, 128.4, 128.8, 130.0, 130.7, 138.0, 141.9, 146.1, 164.9; LC-MS (ESI) *m/z* 299 ([M + 1]^+^).

*N-Methyl-N-phenyl-2-(4-o-tolyl-1H-1,2,3-triazol-1-yl)acetamide* (**2eak**). Colorless oil; Yield: 98. ^1^H-NMR (CDCl_3_) δ 2.45 (s, 3H), 3.32 (s, 3H), 4.97 (s, 2H), 7.22–7.24 (m, 3H), 7.29–7.31 (m, 2H), 7.42 (m, 1H), 7.47–7.50 (m, 2H), 7.76 (m, 1H), 7.86 (s, 1H); ^13^C-NMR (DMSO-*d*_6_) δ 21.1, 37.3, 51.0, 124.9, 126.0, 127.5, 127.7, 128.0, 128.4, 130.0, 130.9, 134.7, 141.9, 145.1, 164.9; LC-MS (ESI) *m/z* 299 ([M + 1]^+^).

*1-Morpholino-3-(4-phenyl-1H-1,2,3-triazol-1-yl)propan-1-one* (**2aca**). Colorless oil; Yield: 94%. ^1^H-NMR (CDCl_3_) δ 2.96 (t, *J* = 6.2 Hz, 2H), 3.38 (t, *J* = 4.9 Hz, 2H), 3.56–3.62 (m, 6H), 4.74 (t, *J* = 6.2 Hz, 2H), 7.30 (m, 1H), 7.38–7.41 (m, 2H), 7.78–7.81 (m, 2H), 7.93 (s, 1H); ^13^C-NMR (DMSO-*d*_6_) δ 32.4, 41.5, 45.2, 45.8, 65.97, 65.98, 121.8, 125.1, 127.8, 128.9, 130.8, 146.0, 168.0; LC-MS (ESI) *m/z* 287 ([M + 1]^+^).

*3-[4-(4-Methoxyphenyl)-1H-1,2,3-triazol-1-yl]-1-morpholinopropan-1-one* (**2acb**). Colorless oil; Yield: 90%. ^1^H-NMR (CDCl_3_) δ 2.96 (t, *J* = 6.2 Hz, 2H), 3.38 (t, *J* = 4.9 Hz, 2H), 3.56–3.62 (m, 6H), 3.81 (s, 3H), 4.72 (t, *J* = 6.2 Hz, 2H), 6.92 (d, *J* = 8.8 Hz, 2H), 7.72 (d, *J* = 8.8 Hz, 2H), 7.83 (s, 1H); ^13^C-NMR (DMSO-*d*_6_) δ 32.5, 41.5, 45.2, 45.7, 55.1, 65.97, 65.99, 114.3, 120.8, 123.4, 126.4, 146.0, 158.9, 168.1; LC-MS (ESI) *m/z* 317 ([M + 1]^+^).

*4-[1-(3-Morpholino-3-oxopropyl)-1H-1,2,3-triazol-4-yl]benzonitrile* (**2acc**). White solid; Yield: 90%. ^1^H-NMR (500 MHz, CDCl_3_) δ 3.01 (t, *J* = 5.9 Hz, 2H), 3.42 (t, *J* = 4.9 Hz, 2H), 3.59-3.65 (m, 6H), 4.80 (t, *J* = 5.9 Hz, 2H), 7.94 (d, *J* = 8.5 Hz, 2H), 7.71 (d, *J* = 8.5 Hz, 2H), 8.08 (s, 1H); ^13^C NMR (125 MHz, DMSO-*d*_6_) δ 32.3, 41.5, 45.1, 46.0, 65.95, 65.97, 110.0, 118.8, 123.6, 125.6, 133.0, 135.3, 144.4, 168.0; LC-MS (ESI) *m/z* 312 ([M + 1]^+^).

*1-Morpholino-3-[4-(thiophen-3-yl)-1H-1,2,3-triazol-1-yl]propan-1-one* (**2acd**). Yellow oil; Yield: 98%. ^1^H-NMR (CDCl_3_) δ 2.95 (t, *J* = 6.2 Hz, 2H), 3.37 (t, *J* = 4.9 Hz, 2H), 3.55–3.61 (m, 6H), 4.72 (t, *J* = 6.1 Hz, 2H), 7.34 (dd, *J* = 3.0, 5.0 Hz, 1H), 7.42 (dd, *J* = 1.3, 5.0 Hz, 1H), 7.63 (dd, *J* = 1.2, 3.0 Hz, 1H), 7.83 (s, 1H); ^13^C-NMR (DMSO-*d*_6_) δ 32.5, 41.5, 45.2, 45.7, 65.96, 65.98, 120.6, 121.6, 125.7, 127.1, 132.1, 142.6, 168.0; LC-MS (ESI) *m/z* 293 ([M + 1]^+^).

*1-Morpholino-3-[4-(pyridin-2-yl)-1H-1,2,3-triazol-1-yl]propan-1-one* (**2ace**). Light yellow oil; Yield: 89%. ^1^H-NMR (CDCl_3_) δ 2.99 (t, *J* = 6.5 Hz, 2H), 3.39 (t, *J* = 4.8 Hz, 2H), 3.57–3.63 (m, 6H), 4.77 (t, *J* = 6.5 Hz, 2H), 7.20 (ddd, *J* = 1.0, 4.9, 7.5 Hz, 1H), 7.74 (dt, *J* = 1.7, 7.7 Hz, 1H), 8.11 (d, *J* = 8.0 Hz, 1H), 8.25 (s, 1H), 8.56 (d, *J* = 4.9 Hz, 1H); ^13^C-NMR (DMSO-*d*_6_) δ 32.4, 41.5, 45.1, 45.8, 65.97, 65.99, 119.3, 122.9, 123.6, 137.2, 147.0, 149.6, 150.0, 168.1; LC-MS (ESI) *m/z* 288 ([M + 1]^+^).

*3-[4-(3-Methoxyphenyl)-1H-1,2,3-triazol-1-yl]-1-morpholinopropan-1-one* (**2acg**). Colorless oil; Yield: 98%. ^1^H-NMR (CDCl_3_) δ 2.96 (t, *J* = 6.2 Hz, 2H), 3.37 (t, *J* = 4.9 Hz, 2H), 3.56–3.62 (m, 6H), 3.83 (s, 3H), 4.73 (t, *J* = 6.2 Hz, 2H), 6.85 (ddd, *J* = 1.2, 2.6, 8.0 Hz, 1H), 7.29 (t, *J* = 7.8 Hz, 1H), 7.33 (td, *J* = 1.3, 7.6 Hz, 1H), 7.40 (dd, *J* = 1.5, 2.5 Hz, 1H), 7.92 (s, 1H); ^13^C-NMR (DMSO-*d*_6_) δ 32.4, 41.5, 45.2, 45.8, 55.1, 65.98, 66.00, 110.3, 13.5, 117.5, 122.1, 130.1, 132.2, 146.0, 459.7, 168.0; LC-MS (ESI) *m/z* 317 ([M + 1]^+^).

*3-[4-(2-Methoxyphenyl)-1H-1,2,3-triazol-1-yl]-1-morpholinopropan-1-one* (**2ach**). Light yellow oil; Yield: 99%. ^1^H-NMR (CDCl_3_) δ 3.00 (t, *J* = 6.5 Hz, 2H), 3.37 (t, *J* = 4.9 Hz, 2H), 3.56–3.61 (m, 6H), 3.91 (s, 3H), 4.74 (t, *J* = 6.5 Hz, 2H), 6.95 (dd, *J* = 0.7, 8.3 Hz, 1H), 7.05 (dt, *J* = 1.0, 7.5 Hz, 1H), 7.29 (ddd, *J* = 1.7, 7.3, 8.3 Hz, 1H), 8.11 (s, 1H), 8.28 (dd, *J* = 1.7, 7.7 Hz, 1H); ^13^C-NMR (DMSO-*d*_6_) δ 32.6, 41.5, 45.2, 45.6, 55.4, 66.0 (2C), 111.5, 119.2, 120.6, 124.3, 126.5, 128.8, 141.6, 155.3, 168.1; LC-MS (ESI) *m/z* 317 ([M + 1]^+^).

*1-Morpholino-3-(4-p-tolyl-1H-1,2,3-triazol-1-yl)propan-1-one* (**2aci**). Colorless oil; Yield: 93%. ^1^H-NMR (CDCl_3_) δ 2.35 (s, 3H), 2.96 (t, *J* = 6.2 Hz, 2H), 3.37 (t, *J* = 4.9 Hz, 2H), 3.55–3.61 (m, 6H), 4.72 (t, *J* = 6.2 Hz, 2H), 7.20 (d, *J* = 7.9 Hz, 2H), 7.68 (d, *J* = 8.1 Hz, 2H), 7.87 (s, 1H); ^13^C-NMR (DMSO-*d*_6_) δ 20.8, 32.4, 41.5, 45.2, 45.8, 65.96, 65.98, 121.4, 125.0, 128.1, 129.4, 137.0, 146.1, 168.0; LC-MS (ESI) *m/z* 301 ([M + 1]^+^).

*1-Morpholino-3-(4-m-tolyl-1H-1,2,3-triazol-1-yl)propan-1-one* (**2acj**). Colorless oil; Yield: 98%. ^1^H-NMR (CDCl_3_) δ 2.37 (s, 3H), 2.96 (t, *J* = 6.2 Hz, 2H), 3.38 (t, *J* = 4.9 Hz, 2H), 3.56–3.62 (m, 6H), 4.74 (t, *J* = 6.2 Hz, 2H), 7.12 (d, *J* = 7.8 Hz, 1H), 7.28 (t, *J* = 7.7 Hz, 1H), 7.57 (d, *J* = 7.8 Hz, 1H), 7.65 (s, 1H), 7.91 (s, 1H); ^13^C-NMR (DMSO-*d*_6_) δ 21.1, 32.4, 41.5, 45.2, 45.8, 65.98, 66.00, 121.7, 122.3, 125.6, 128.4, 128.8, 130.7, 138.0, 146.2, 168.1; LC-MS (ESI) *m/z* 301 ([M + 1]^+^).

*1-Morpholino-3-(4-o-tolyl-1H-1,2,3-triazol-1-yl)propan-1-one* (**2ack**). Light yellow oil; Yield: 90%. ^1^H-NMR (CDCl_3_) δ 2.45 (s, 3H), 3.00 (t, *J* = 6.3 Hz, 2H), 3.40 (t, *J* = 4.9 Hz, 2H), 3.57–3.62 (m, 6H), 4.77 (t, *J* = 6.3 Hz, 2H), 7.21–7.27 (m, 3H), 7.73 (m, 1H), 7.82 (s, 1H); ^13^C-NMR (DMSO-*d*_6_) δ 21.1, 32.5, 41.5, 45.2, 45.8, 66.0, 66.02, 123.6, 126.0, 127.7, 128.1, 130.1, 130.9, 134.8, 145.4, 168.1; LC-MS (ESI) *m/z* 301 ([M + 1]^+^).

*3-{4-[4-(Dimethylamino)phenyl]-1H-1,2,3-triazol-1-yl}-1-morpholinopropan-1-one* (**2acl**). Light yellow solid; Yield: 99%. ^1^H-NMR (CDCl_3_) δ 2.96 (t, *J* = 6.3 Hz, 2H), 2.97 (s, 6H), 3.38 (t, *J* = 4.9 Hz, 2H), 3.57–3.62 (m, 6H), 4.72 (t, *J* = 6.3 Hz, 2H), 6.75 (d, *J* = 9.0 Hz, 2H), 7.67 (d, *J* = 9.0 Hz, 2H), 7.77 (s, 1H); ^13^C-NMR (DMSO-*d*_6_) δ 32.5, 40.0, 41.5, 45.2, 45.7, 65.97, 65.99, 112.4, 119.9, 126.0, 130.2, 146.7, 149.9, 168.1; LC-MS (ESI) *m/z* 330 ([M + 1]^+^).

*1-Morpholino-3-[4-(4-phenoxyphenyl)-1H-1,2,3-triazol-1-yl]propan-1-one* (**2acm**). Light yellow oil; Yield: 99%. ^1^H-NMR (CDCl_3_) δ 2.98 (t, *J* = 6.1 Hz, 2H), 3.39 (t, *J* = 4.8 Hz, 2H), 3.58–3.63 (m, 6H), 4.75 (t, *J* = 6.1 Hz, 2H), 7.02–7.05 (m, 4H), 7.12 (m, 1H), 7.32–7.35 (m, 2H), 7.77 (d, *J* = 8.7 Hz, 2H), 7.89 (s, 1H); ^13^C-NMR (DMSO-*d*_6_) δ 32.44, 41.5, 45.2, 45.8, 65.96, 65.98, 118.8, 118.9, 123.6, 124.2, 126.2, 126.8, 130.1, 145.6, 156.3, 156.4, 168.0; LC-MS (ESI) *m/z* 379 ([M + 1]^+^).

*3-[4-(2,4-Difluorophenyl)-1H-1,2,3-triazol-1-yl]-1-morpholinopropan-1-one* (**2acn**). Light yellow solid; Yield: 92. ^1^H-NMR (CDCl_3_) δ 3.02 (t, *J* = 6.3 Hz, 2H), 3.41 (t, *J* = 4.9 Hz, 2H), 3.60–3.65 (m, 6H), 4.79 (t, *J* = 6.3 Hz, 2H), 6.90 (ddd, *J* = 2.4, 8.7, 11.0 Hz, 1H), 6.99 (m, 1H), 8.03 (d, *J* = 3.8 Hz, 1H), 8.24 (dt, *J* = 6.5, 8.6 Hz, 1H); ^13^C-NMR (DMSO-*d*_6_) δ 32.5, 41.5, 45.2, 45.9, 66.0 (2C), 104.5 (t, *J*_CF_ = 26.0 Hz), 112.3 (dd, *J*_CF_ = 21.1, 3.7 Hz), 115.3 (dd, *J*_CF_ = 11.8, 5.4 Hz), 123.9 (d, *J*_CF_ = 10.4 Hz), 128.5 (dd, *J*_CF_ = 9.6, 5.5 Hz), 138.8 (d, *J*_CF_ = 2.5 Hz), 158.4 (dd, *J*_CF_ = 250.1, 12.6 Hz), 161.7 (dd, *J*_CF_ = 247.5, 12.6 Hz), 168.1; LC-MS (ESI) *m/z* 323 ([M + 1]^+^).

*3-(4-Benzyl-1H-1,2,3-triazol-1-yl)-1-morpholinopropan-1-one* (**2aco**). Colorless oil; Yield: 98%. ^1^H-NMR (CDCl_3_) δ 2.92 (t, *J* = 6.4 Hz, 2H), 3.35 (t, *J* = 4.9 Hz, 2H), 3.53–3.59 (m, 6H), 4.04 (s, 2H), 4.63 (t, *J* = 6.4 Hz, 2H), 7.19 (m, 1H), 7.21–7.29 (m, 4H), 7.32 (s, 1H); ^13^C-NMR (DMSO-*d*_6_) δ 31.3, 32.5, 41.5, 45.2, 45.5, 66.0 (2C), 122.8, 126.1, 128.4, 128.5, 139.6, 145.8, 168.1; LC-MS (ESI) *m/z* 301 ([M + 1]^+^).

## 4. Conclusions

In summary, the yields for secondary α-1,2,3-triazoloamides (80 examples) produced by solid-phase synthetic route ranged from 75 to 94% for six linear steps starting with Merrifield resin (the average yield for each step was over 95%). The parallel solution-phase synthesis generated the target tertiary 1,2,3-triazoloamides (80 examples) with 97%–73% yields for three linear steps from the reaction of amines and chloro-acid chlorides. In addition, the target 1,2,3-triazoloamides were obtained in high purities (>95%) as judged from LC-MS and ^1^H-NMR analyses. This investigation, has led to the development of the solid- and solution-phase route for the synthesis of various 1,2,3-triazoloamides that contain three diversity sites that were introduced in reactions involving amines (R^1^ and R^2^), chloro-acid chlorides (**A**), and terminal acetylenes (R^3^). The strategy allows for a ready access to a large library and is potentially applicable to the preparation of other 1,2,3-triazole derivatives.
